# Clinical Effect of Early vs Late Amyloid Positron Emission Tomography in Memory Clinic Patients: The AMYPAD-DPMS Randomized Clinical Trial

**DOI:** 10.1001/jamaneurol.2023.0997

**Published:** 2023-05-08

**Authors:** Daniele Altomare, Frederik Barkhof, Camilla Caprioglio, Lyduine E. Collij, Philip Scheltens, Isadora Lopes Alves, Femke Bouwman, Johannes Berkhof, Ingrid S. van Maurik, Valentina Garibotto, Christian Moro, Julien Delrieu, Pierre Payoux, Laure Saint-Aubert, Anne Hitzel, José Luis Molinuevo, Oriol Grau-Rivera, Juan Domingo Gispert, Alexander Drzezga, Frank Jessen, Philip Zeyen, Agneta Nordberg, Irina Savitcheva, Vesna Jelic, Zuzana Walker, Paul Edison, Jean-François Demonet, Rossella Gismondi, Gill Farrar, Andrew W. Stephens, Giovanni B. Frisoni

**Affiliations:** 1 Laboratory of Neuroimaging of Aging (LANVIE), University of Geneva, Geneva, Switzerland; 2 Geneva Memory Center, Geneva University Hospitals, Geneva, Switzerland; 3 Neurology Unit, Department of Clinical and Experimental Sciences, University of Brescia, Brescia, Italy; 4 Department of Radiology and Nuclear Medicine, Amsterdam University Medical Centers (UMC)–Location VUmc, Amsterdam, the Netherlands; 5 Institute of Neurology, Institute of Healthcare Engineering, University College London, London, United Kingdom; 6 Alzheimer Center, Department of Neurology, Amsterdam University Medical Centers–Location VUmc, Amsterdam, the Netherlands; 7 Department of Epidemiology and Data Science, Amsterdam University Medical Centers–Location VUmc, Amsterdam, the Netherlands; 8 Laboratory of Neuroimaging and Innovative Molecular Tracers (NIMTlab), Geneva University Neurocenter and Faculty of Medicine, University of Geneva, Geneva, Switzerland; 9 Division of Nuclear Medicine and Molecular Imaging, Geneva University Hospitals, Geneva, Switzerland; 10 Gérontopôle, Department of Geriatrics, Toulouse University Hospital, Toulouse, France; 11 Maintain Aging Research Team, CERPOP, Inserm, Université Paul Sabatier, Toulouse, France; 12 Department of Nuclear Medicine, Toulouse University Hospital, Toulouse, France; 13 Toulouse NeuroImaging Center (ToNIC), UMR1214 Inserm, Université de Toulouse III, Toulouse, France; 14 Barcelonaβeta Brain Research Center (BBRC), Pasqual Maragall Foundation, Barcelona, Spain; 15 H. Lundbeck, Copenhagen, Denmark; 16 Hospital del Mar Medical Research Institute (IMIM), Barcelona, Spain; 17 Centro de Investigación Biomédica en Red de Fragilidad y Envejecimiento Saludable (CIBERFES), Madrid, Spain; 18 Centro de Investigación Biomédica en Red Bioingeniería, Biomateriales y Nanomedicina (CIBER-BBN), Barcelona, Spain; 19 Department of Nuclear Medicine, Faculty of Medicine and University Hospital Cologne, University of Cologne, Cologne, Germany; 20 German Center for Neurodegenerative Diseases (DZNE), Bonn-Cologne, Germany; 21 Institute of Neuroscience and Medicine (INM-2), Molecular Organization of the Brain, Forschungszentrum Jülich, Germany; 22 Department of Psychiatry, Faculty of Medicine and University Hospital Cologne, University of Cologne, Cologne, Germany; 23 Excellence Cluster Cellular Stress Responses in Aging-Related Diseases (CECAD), Medical Faculty, University of Cologne, Cologne, Germany; 24 Department of Neurobiology, Care Sciences and Society, Center of Alzheimer Research, Karolinska Institutet, Stockholm, Sweden; 25 Theme Aging, Karolinska University Hospital, Stockholm, Sweden; 26 Medical Radiation Physics and Nuclear Medicine, Section for Nuclear Medicine, Karolinska University Hospital, Stockholm, Sweden; 27 Cognitive Disorders Clinic, Theme Inflammation and Aging, Karolinska University Hospital–Huddinge, Stockholm, Sweden; 28 Division of Psychiatry, University College London, London, United Kingdom; 29 St Margaret’s Hospital, Essex Partnership University NHS Foundation Trust, Essex, United Kingdom; 30 Division of Neurology, Department of Brain Sciences, Imperial College London, London, United Kingdom; 31 Leenaards Memory Center, Lausanne University Hospital (CHUV), Lausanne, Switzerland; 32 Life Molecular Imaging, Berlin, Germany; 33 GE Healthcare, Amersham, United Kingdom

## Abstract

**Importance:**

Amyloid positron emission tomography (PET) allows the direct assessment of amyloid deposition, one of the main hallmarks of Alzheimer disease. However, this technique is currently not widely reimbursed because of the lack of appropriately designed studies demonstrating its clinical effect.

**Objective:**

To assess the clinical effect of amyloid PET in memory clinic patients.

**Design, Setting, and Participants:**

The AMYPAD-DPMS is a prospective randomized clinical trial in 8 European memory clinics. Participants were allocated (using a minimization method) to 3 study groups based on the performance of amyloid PET: arm 1, early in the diagnostic workup (within 1 month); arm 2, late in the diagnostic workup (after a mean [SD] 8 [2] months); or arm 3, if and when the managing physician chose. Participants were patients with subjective cognitive decline plus (SCD+; SCD plus clinical features increasing the likelihood of preclinical Alzheimer disease), mild cognitive impairment (MCI), or dementia; they were assessed at baseline and after 3 months. Recruitment took place between April 16, 2018, and October 30, 2020. Data analysis was performed from July 2022 to January 2023.

**Intervention:**

Amyloid PET.

**Main Outcome and Measure:**

The main outcome was the difference between arm 1 and arm 2 in the proportion of participants receiving an etiological diagnosis with a very high confidence (ie, ≥90% on a 50%-100% visual numeric scale) after 3 months.

**Results:**

A total of 844 participants were screened, and 840 were enrolled (291 in arm 1, 271 in arm 2, 278 in arm 3). Baseline and 3-month visit data were available for 272 participants in arm 1 and 260 in arm 2 (median [IQR] age: 71 [65-77] and 71 [65-77] years; 150/272 male [55%] and 135/260 male [52%]; 122/272 female [45%] and 125/260 female [48%]; median [IQR] education: 12 [10-15] and 13 [10-16] years, respectively). After 3 months, 109 of 272 participants (40%) in arm 1 had a diagnosis with very high confidence vs 30 of 260 (11%) in arm 2 (*P* < .001). This was consistent across cognitive stages (SCD+: 25/84 [30%] vs 5/78 [6%]; *P* < .001; MCI: 45/108 [42%] vs 9/102 [9%]; *P* < .001; dementia: 39/80 [49%] vs 16/80 [20%]; *P* < .001).

**Conclusion and Relevance:**

In this study, early amyloid PET allowed memory clinic patients to receive an etiological diagnosis with very high confidence after only 3 months compared with patients who had not undergone amyloid PET. These findings support the implementation of amyloid PET early in the diagnostic workup of memory clinic patients.

**Trial Registration:**

EudraCT Number: 2017-002527-21

## Key Points

**Question:**

Is early amyloid positron emission tomography (PET) clinically useful in memory clinic patients?

**Findings:**

In this randomized clinical trial, we demonstrated that performing amyloid PET early in the diagnostic workup (within 1 month) allowed 40% of memory clinic patients to receive an etiological diagnosis with very high diagnostic confidence after only 3 months, 3.5 times more frequently than patients who had not undergone amyloid PET (11%).

**Meaning:**

This study adds evidence to previous studies showing that amyloid PET has a relevant clinical effect in memory clinic patients.

## Teaser

This randomized clinical trial compares data for the timing of amyloid positron emission tomography for patients with concerns about cognitive decline to determine its clinical effect early in the diagnostic workup (within 1 month).

## Introduction

Amyloid deposition in the brain is one of the main hallmarks of Alzheimer disease (AD) and is considered one of the strongest risk factors of dementia.^1^ The development of Aβ-amyloid positron emission tomography (amyloid PET) tracers^2^ has allowed the direct assessment of amyloid deposition in vivo. Despite the increasing use of amyloid PET in clinical practice, real-world evidence about its clinical utility and cost-effectiveness is still limited.^3^ Indeed, although several studies have been published so far,^4,5,6,7,8,9,10^ they lack key features (eg, a control group) to provide conclusive evidence.

In the United States, the IDEAS study provided strong evidence on the association of amyloid PET with subsequent changes in patients’ diagnosis and management in a large (n = 11 409) but selected cohort consisting of Medicare beneficiaries 65 years or older^9^ meeting the appropriate use criteria for amyloid PET.^11^ However, IDEAS was a nonrandomized and noncontrolled study, and its cohort cannot be considered wholly representative of a general memory clinic population. Indeed, IDEAS patients were recruited by dementia specialists from their clinical practices, where clinical assessment is more heterogeneous and may be less specific than in memory clinics. Moreover, the appropriate use criteria for amyloid PET result from expert recommendations, but their ability to select patients who can actually benefit from amyloid PET has been questioned,^12,13^ and the full potential of amyloid PET might not have been examined. In particular, the appropriate use criteria did not include participants with subjective cognitive decline (SCD), a group of individuals accounting for 21% to 29% of the memory clinic population,^14,15^ who were consequently not included in IDEAS.

The Amyloid Imaging to Prevent Alzheimer’s Disease Diagnostic and Patient Management Study (AMYPAD-DPMS) was designed as a prospective, multicenter, randomized clinical trial^16^ and is the largest European study assessing the clinical effect of amyloid PET in memory clinic patients. It aims to fill the current evidence gap by providing strong evidence on the clinical utility and cost-effectiveness of amyloid PET.

In the present work, we investigated whether participants allocated to undergo amyloid PET early in their diagnostic workup received an etiological diagnosis with very high diagnostic confidence after 3 months more frequently than those who had not undergone amyloid PET yet. Moreover, we also assessed whether early amyloid PET is associated with more frequent changes in diagnosis, diagnostic confidence, and treatment plan. Finally, we examined the real-world use and clinical effect of unrestricted amyloid PET imaging in a free-choice group.

## Methods

### Study Design

AMYPAD-DPMS is a prospective, multicenter, randomized clinical trial. The ethics committees of all recruiting memory clinics approved the study. All participants gave written informed consent. The trial was registered with EudraCT (2017-002527-21), and the trial protocol appears in Supplement 2.

**Figure 1.**
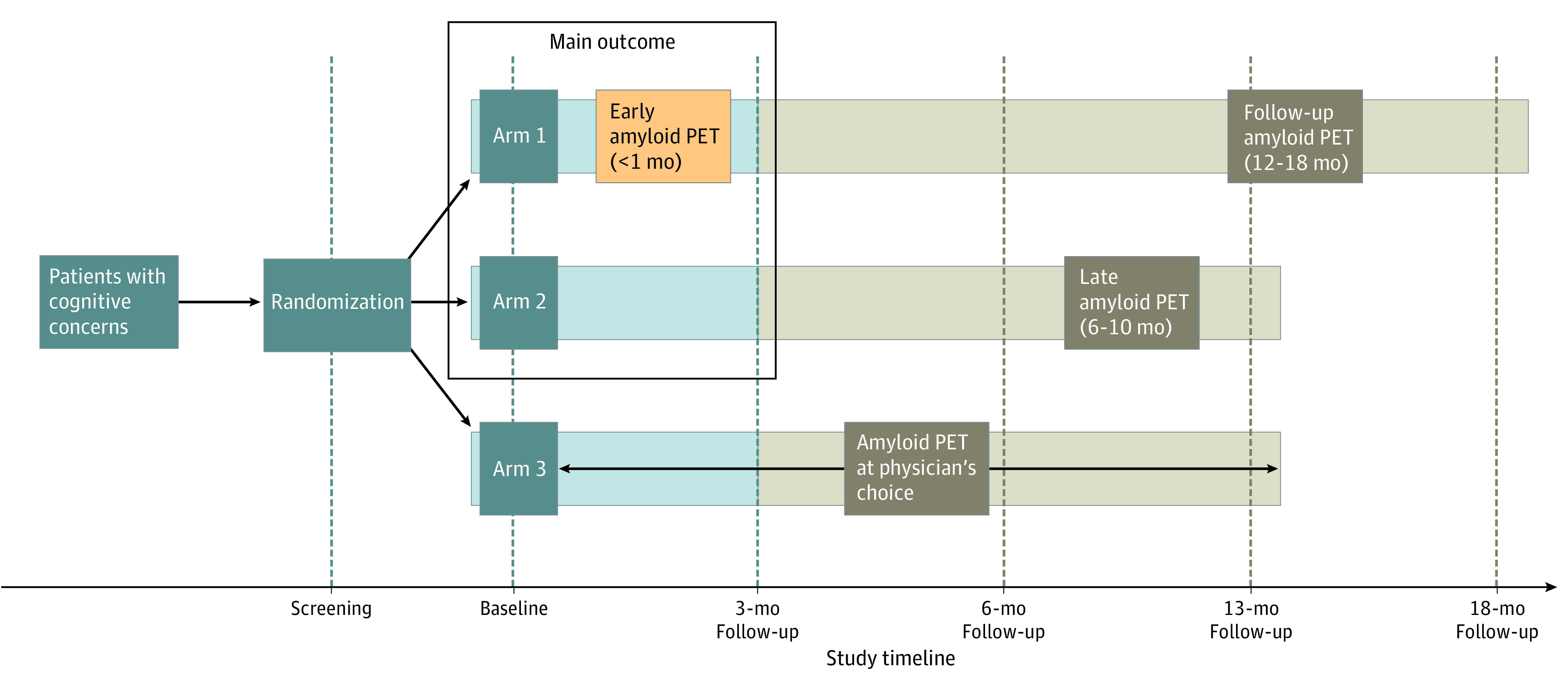
Study Design for Early vs Late Positron Emission Tomography (PET) Tan boxes indicate features of the study design not presented in the current work. The 13-month visit, the follow-up amyloid PET (to test the hypothesis that amyloid load is stable over 12-18 months), and the corresponding 18-month visit for arm 1 were made optional for logistic reasons. These measures were put in place to keep the end of the study timeline despite a prolonged recruitment (originally planned to be completed by June 30, 2020), which was granted to the recruiting memory clinics when in spring 2020 the COVID-19 pandemic brought recruitment to a complete halt.

Figure 1 illustrates the AMYPAD-DPMS study design, which has been described in a previous article.^16^ Briefly, participants were allocated (using a minimization method^17^) into 3 study groups: for arm 1, amyloid PET was performed early in the diagnostic workup (ie, within 1 month from baseline); arm 2, amyloid PET was performed late in the diagnostic workup (ie, after a mean [SD] 8 [2] months from baseline); and arm 3, amyloid PET was performed when the managing physician chose to request it (the free-choice arm). Arm 1 and arm 2 allowed us to assess the main outcome of the study, while the scientific rationale of arm 3 was to describe the real-world use and clinical effect of unrestricted amyloid PET imaging in memory clinic patients. More information about the allocation procedure and allocation ratio is reported in the eMethods in Supplement 1.

All AMYPAD-DPMS participants underwent up to 5 clinical visits: screening and baseline, and after 3, 6, 13, and 18 months (only for arm 1, not mandatory). During these visits, sociodemographic and clinical variables were collected, including cognitive stage, etiological diagnosis, diagnostic confidence, and treatment plan. The managing physicians indicated cognitive stage (ie, SCD plus clinical features increasing the likelihood of preclinical AD [SCD+], mild cognitive impairment [MCI], dementia) and etiological diagnoses (eg, AD, cerebrovascular disease, frontotemporal lobar degeneration, dementia with Lewy bodies), rated diagnostic confidence (by ticking the percentage corresponding to their appraisal of the diagnostic confidence on a visual numeric scale made of percentages organized spatially from 50% to 100% with incremental 5% intervals), and defined the treatment plan for each participant at each visit. In the current work, we focus only on the baseline and 3-month follow-up as related to the assessment of the primary outcome.

Participants underwent amyloid PET with either [18F]flutemetamol (Vizamyl, GE Healthcare) or [18F]florbetaben (Neuraceq, Life Molecular Imaging) as amyloid PET tracers. Because AMYPAD-DPMS focuses on routine clinical practice, amyloid PET images were visually assessed by trained local nuclear medicine physicians using agency-approved reading methods for the 2 tracers. Assessment was performed using local software and workstations and in combination with structural imaging, if available.

### Participants

Participants were memory clinic patients with variable baseline cognitive stage, ranging from SCD+ to MCI and dementia, enrolled from 8 European memory clinics. Enrollment strategies have been described in a previous article.^18^

Participants were enrolled from 8 European academic memory clinics: University and University Hospital of Geneva (Geneva, Switzerland), Amsterdam University Medical Center, Location VUmc (Amsterdam UMC; Amsterdam, the Netherlands), Centre Hospitalier Universitaire de Toulouse (CHUT; Toulouse, France), Barcelonaβeta Brain Research Center (Barcelona, Spain), University of Cologne (Cologne, Germany), University College London (UCL; London, United Kingdom), Karolinska Institutet (Stockholm, Sweden), and Centre Hospitalier Universitaire Vaudois (Lausanne, Switzerland). Three academic memory clinics (Amsterdam UMC, CHUT, and UCL) extended the recruitment to external nonacademic partnering memory clinics.

Ethnicity data were collected by members of the AMYPAD-DPMS study team at the local recruiting memory clinics, who chose among options on an electronic case report form. The options were African, Asian or Asian British, Black or Black British, British, Caribbean, Chinese, Indian, Irish, multiracial (listed as “mixed”), Pakistani, White, White and Black African, White and Black Caribbean, any other Asian background, any other multiracial background, any other White background, and not stated. Subsequently, numbers of participants identified as British, Irish, White, and any other White background were grouped into a White category for reporting baseline characteristics because of the small numbers in each ethnic group.

Major inclusion and exclusion criteria are reported in detail in the eMethods in Supplement 1. Briefly, main inclusion criteria were as follows: the patient must have had a cognitive concern considered by the managing physician to be possibly due to AD; the patient must have been undergoing a diagnostic workup, including a recent (not >12 months) magnetic resonance imaging (MRI) scan and/or computed tomography (CT) scan; and the managing physician must have felt that knowledge of the patient’s brain amyloid status could increase diagnostic confidence and alter their diagnosis and/or management. The main exclusion criterion was previous amyloid PET and/or other AD biomarker workup before screening.

### Outcomes

The main outcome of the study was the proportion of participants receiving an etiological diagnosis with very high diagnostic confidence (ie, ≥90%) after 3 months (the period when the diagnostic process is generally completed) in arm 1 vs arm 2. The choice of this main outcome was based on previous studies showing that the etiological diagnosis of patients with a diagnostic confidence greater than 90% does not change following amyloid PET^19^ and that the maximal mean diagnostic confidence post amyloid PET is 86% to 93%,^5,6,7,10^ suggesting that this level of diagnostic confidence is a strong, achievable, and replicable reference standard.

Secondary outcomes were change in etiological diagnosis, change in diagnostic confidence, and change in treatment plan. First, etiological diagnoses were grouped into 3 main categories: AD, including all the diagnoses involving AD (AD and mixed AD [ie, AD in comorbidity with other conditions]); non-AD, including all the remaining diagnoses/conditions without AD in comorbidity; and undetermined, when the managing physician is not confident in making any etiological diagnosis. Changes in etiological diagnosis were considered as consistent with the amyloid PET result when (1) participants with a baseline diagnosis of AD were reclassified as non-AD or undetermined after negative amyloid PET; (2) participants with a baseline diagnosis of non-AD were reclassified as AD or undetermined after positive amyloid PET; (3) participants with an undetermined diagnosis at baseline were reclassified as AD after positive amyloid PET or as non-AD after negative amyloid PET.

Second, diagnostic confidence was rated only for participants who received an etiological diagnosis. Specifically, we assessed changes in diagnostic confidence only for participants with a baseline etiological diagnosis of AD or non-AD that was confirmed after 3 months.

Third, to assess change in treatment plan, we determined the proportion of cognition-specific medications (eg, acetylcholinesterase inhibitors, memantine, supplements such as ginkgo or Souvenaid) that were not prescribed, introduced, discontinued, and maintained after 3 months. The introduction or discontinuation of any of these medications was considered a change in treatment plan.

The analyses of the primary and secondary outcomes were performed for participants in arm 1, arm 2, and arm 3, both on the whole sample and according to cognitive stage (SCD+, MCI, and dementia). For arm 3, we assessed the proportion of participants who underwent an amyloid PET during the study, median time from baseline to performance of the scan, and reasons the scan was requested.

### Statistical Analyses

Information on sample size determination is reported in the eMethods in Supplement 1. To assess the main outcome of the study, we performed an intention-to-treat analysis, focusing on all participants for whom the main outcome could be assessed. For the secondary outcomes, we performed per-protocol analyses, removing data for participants who did not adhere to the study protocol. We used cases with complete outcome information for the assessment of the main and secondary outcomes (information on cognitive stage, etiological diagnosis, diagnostic confidence, and treatment plan was available for all participants).

Continuous variables are described as median and IQR and categorical variables as numbers and percentages. The main outcome of the study was assessed in the whole group and for the 3 individual cognitive stages (SCD+, MCI, dementia) using tests for equality of proportions. The 3 tests on the individual cognitive stages were adjusted using Bonferroni corrections. Subsequently, we assessed the main outcome also including arm 3, and post hoc pairwise comparisons (ie, arm 1 vs arm 2, arm 1 vs arm 3, and arm 2 vs arm 3) were adjusted using Bonferroni corrections. For the analyses of the secondary end points, differences were assessed using a Kruskal-Wallis rank sum test for continuous variables or test for equality of proportions for categorical variables. In case the number of groups in the comparison was larger than 2, post hoc pairwise comparisons (Dunn all-pairs rank comparison test for continuous variables or pairwise comparisons for proportions) were adjusted using Bonferroni correction. Significance was set at *P* < .05.

Data analysis was performed from July 2022 to January 2023. The statistical analysis of the main outcome was performed with SPSS version 27. The statistical analyses of the secondary outcomes were performed with R version 4.1.2 (R Foundation for Statistical Computing).

## Results

### Participants

**Figure 2.**
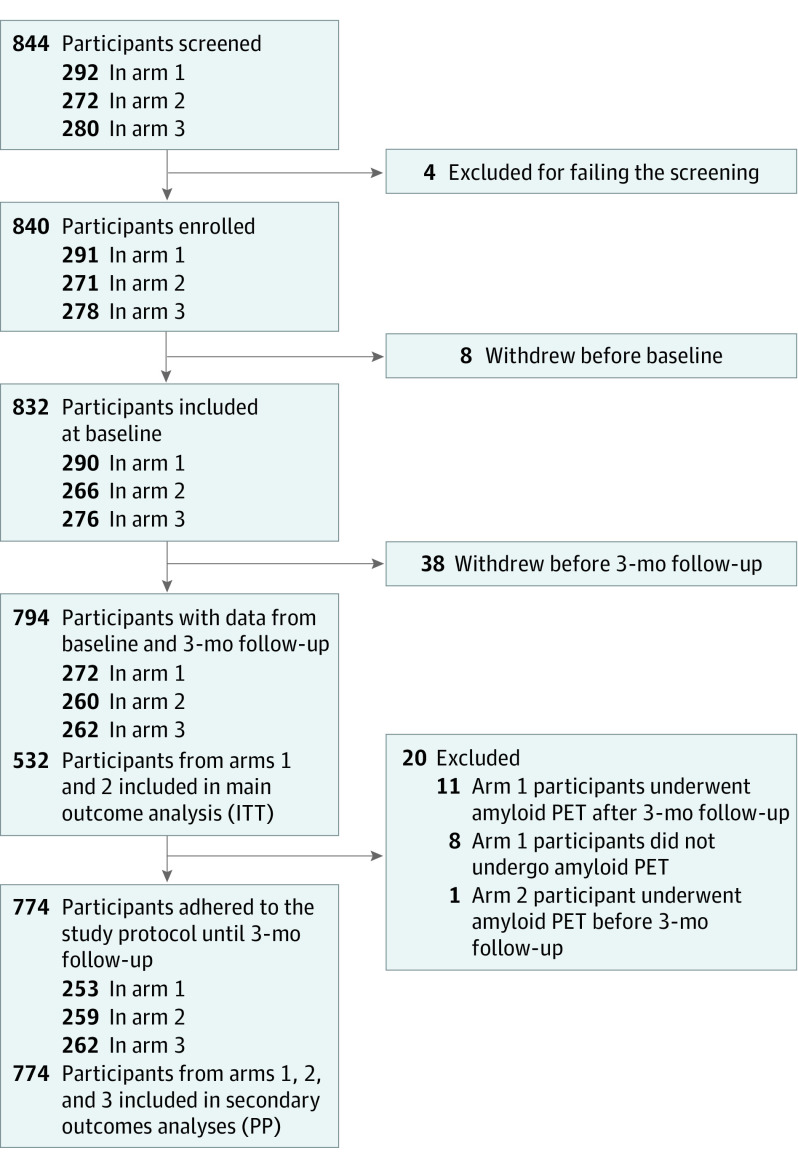
Flowchart for the Amyloid Imaging to Prevent Alzheimer’s Disease Diagnostic and Patient Management Study (AMYPAD-DPMS) Reasons for not undergoing the baseline or 3-month visit included the following: incident comorbidity (n = 6), concerns about radiation exposure (n = 5), emigration (n = 4), COVID-19 lockdown (n = 3), insufficient cost/benefit ratio (n = 3), managing physician’s advice (n = 3), death (n = 2), inability to understand the study design (n = 1), and participation in a competing clinical trial (n = 1), while the remaining participants (n = 18) did not give any explanation. ITT indicates intention to treat; PET, positron emission tomography; PP, per protocol.

Figure 2 shows the study flow. Participants were recruited from April 16, 2018, to October 30, 2020. From 844 screened patients, 840 were enrolled. The baseline features of the AMYPAD-DPMS participants have been exhaustively described in a previous article.^18^ Among them, 794 participants also underwent the 3-month visit and were therefore considered for the main outcome analysis. After removing data for participants who did not adhere to the study protocol, 774 participants were considered for the secondary outcome analyses.

At baseline, 785 of 794 participants (99%) underwent structural imaging (ie, MRI or CT), and 788 of 794 (99%) underwent cognitive screening (ie, Mini-Mental State Examination), with no difference among arms. The Table illustrates the baseline features of the AMYPAD-DPMS participants included in the main outcome analysis, in the whole sample and by study arm. eTable 1 in Supplement 1 illustrates the baseline features of the AMYPAD-DPMS participants by baseline cognitive stage, and eTable 2 in Supplement 1 illustrates the exact baseline etiological diagnoses. Some participants underwent diagnostic examinations (eg, cerebrospinal fluid, fluorodeoxyglucose PET) within 3 months, but no differences among arms were observed (eTable 3 in Supplement 1).

**Table.** Baseline Characteristics of AMYPAD-DPMS Participants^a^

Characteristic	No. (%) [No. with data missing]			
	Whole sample (N = 794)	Arm 1 (n = 272)	Arm 2 (n = 260)	Arm 3 (n = 262)
**Sociodemographic**				
Age, median (IQR), y	71 (65-77)	71 (65-77)	71 (65-77)	72 (66-77)
Gender				
Male	435 (55)	150 (55)	135 (52)	150 (57)
Female	359 (45)	122 (45)	125 (48)	112 (43)
Education, median (IQR), y	12 (10-15)	12 (10-15)	13 (10-16)	12 (10-15)
White ethnicity^b^	701 (97) [74]	242 (97) [22]	230 (98) [25]	229 (97) [27]
**Mental status**				
MMSE score, median (IQR)	27 (23-29) [6]	27 (23-29) [2]	26 (23-29) [2]	26 (23-29) [2]
History of anxiety	161 (20)	46 (17)	50 (19)	65 (25)
HADS anxiety score, median (IQR)	6 (3-9) [15]	5 (3-8) [4]	6 (4-10) [8]	6 (3-9) [3]
History of depression	233 (29)	84 (31)	74 (28)	75 (29)
Depression in last 5 y	159 (27) [203]	57 (28) [67]	51 (27) [68]	51 (26) [68]
HADS depression score, median (IQR)	4 (2-7) [15]	4 (2-7) [4]	4 (2-7) [8]	4 (2-6) [3]
**Dementia risk factors**				
Hypertension	302 (49) [178]	97 (46) [61]	92 (47) [65]	113 (54) [52]
Body mass index, median (IQR)^c^	26 (23-29) [21]	26 (24-29) [7]	26 (23-29) [7]	26 (23-28) [7]
Reported cardiovascular events	308 (39)	99 (36)	96 (37)	113 (43)
Reported head injury	103 (13)	42 (15)	33 (13)	28 (11)
Smoking	90 (11)	32 (12)	32 (12)	26 (10)
Alcohol abuse	34 (4)	13 (5)	8 (3)	13 (5)
Vitamin deficiency	100 (13)	32 (12)	41 (16)	27 (10)
Self-sufficiency				
Disabilities	59 (7)	20 (7)	21 (8)	18 (7)
Living in institution	5 (0)	0	4 (2)	1 (0)
Still working	112 (14)	31 (11)	43 (17)	38 (15)
**Drugs and patient management**				
≥1 Cognition-specific medications	90 (11)	31 (11)	31 (12)	28 (11)
Other medications, median (IQR)	3 (1-5)	3 (1-5)	4 (1-6)	3 (2-5)
Nonpharmacological interventions	119 (15)	36 (13)	41 (16)	42 (16)
Cognitive stage at baseline				
SCD+	239 (30)	84 (31)	78 (30)	77 (29)
MCI	318 (40)	108 (40)	102 (39)	108 (41)
Dementia	237 (30)	80 (29)	80 (31)	77 (29)
Etiological diagnosis at baseline				
AD	319 (40)	95 (35)	110 (42)	114 (44)
Non-AD	132 (17)	45 (17)	44 (17)	43 (16)
Undetermined	343 (43)	132 (49)	106 (41)	105 (40)
Diagnostic confidence at baseline, median (IQR), %				
In AD etiological diagnoses	75 (60-80)	75 (68-80)	75 (61-80)	75 (60-80)
In non-AD etiological diagnoses	70 (60-80)	70 (60-80)	70 (60-80)	70 (55-80)

Abbreviations: AD, Alzheimer disease; HADS, Hospital Anxiety and Depression Scale; MCI, mild cognitive impairment; MMSE, Mini-Mental State Examination; SCD+, subjective cognitive decline plus clinical features increasing the likelihood of preclinical AD.

^a^ The table illustrates the main sociodemographic and clinical features of the AMYPAD-DPMS participants included in the main outcome analysis (intention-to-treat analysis). At baseline, cognitive stages and etiological diagnoses were based on clinical and cognitive assessment and MRI or computed tomography.

^b^ Ethnicity data were collected by members of the AMYPAD-DPMS study team at the local recruiting memory clinics, who chose among options on an electronic case report form. White ethnicity included those reported as British, Irish, White, and any other White background; the other groups were African, Asian or Asian British, Black or Black British, Caribbean, Chinese, Indian, multiracial (listed as “mixed”), Pakistani, White and Black African, White and Black Caribbean, any other Asian background, any other multiracial background, and not stated.

^c^ Calculated as weight in kilograms divided by height in meters squared.

Information on the managing physicians involved in the clinical assessment of the AMYPAD-DPMS participants is reported in the eResults in Supplement 1. Information on adverse events is reported in eTable 4 in Supplement 1.

### Prevalence of Amyloid PET Positivity

eFigure 1 in Supplement 1 illustrates the prevalence of amyloid PET positivity across cognitive stages and etiological diagnoses. A total of 736 participants underwent amyloid PET during the study course (384 with [18F]flutemetamol and 352 with [18F]florbetaben, without considering the repeated amyloid PET scans of arm 1 participants). They were positive for amyloid in 369 of 736 cases (50%). The prevalence of amyloid positivity increased with the severity of cognitive stage (*P* < .001): 67 of 222 participants (30%) with SCD+, 146 of 297 (49%) with MCI, and 156 of 217 participants (72%) with dementia. The prevalence of amyloid positivity increased in participants with a baseline diagnosis of AD (201/293, 69%) vs participants with non-AD (44/122, 36%; *P* < .001) or undetermined diagnosis (124/321, 39%; *P* < .001) at baseline.

### Request for Amyloid PET in Arm 3

The proportion of enrolled arm 3 participants who underwent amyloid PET during the study was 243 of 278 participants (87%). This proportion is not different from that of arm 1 and arm 2 participants who underwent amyloid PET during the study, 268 of 291 (92%) and 234 of 271 (86%), respectively (*P* = .07). In arm 3, we observed that the time from baseline to performing an amyloid PET was a median (IQR) 46 (26-84) days, and 191 of 278 participants (69%) underwent amyloid PET before the 3-month follow-up visit.

We observed that the main reasons for performing an amyloid PET were related to diagnostic uncertainty (unclear diagnosis in 147/243 cases [60%] and need to prove or exclude AD in 105/243 cases [43%] and 78/243 cases [32%], respectively), followed by participant preference (patient wanted an amyloid PET scan in 26/243 cases [11%] and refused lumbar puncture in 13/243 cases [5%]) (eTable 5 in Supplement 1).

### Participants With Very High Diagnostic Confidence After 3 Months (Main Outcome)

**Figure 3.**
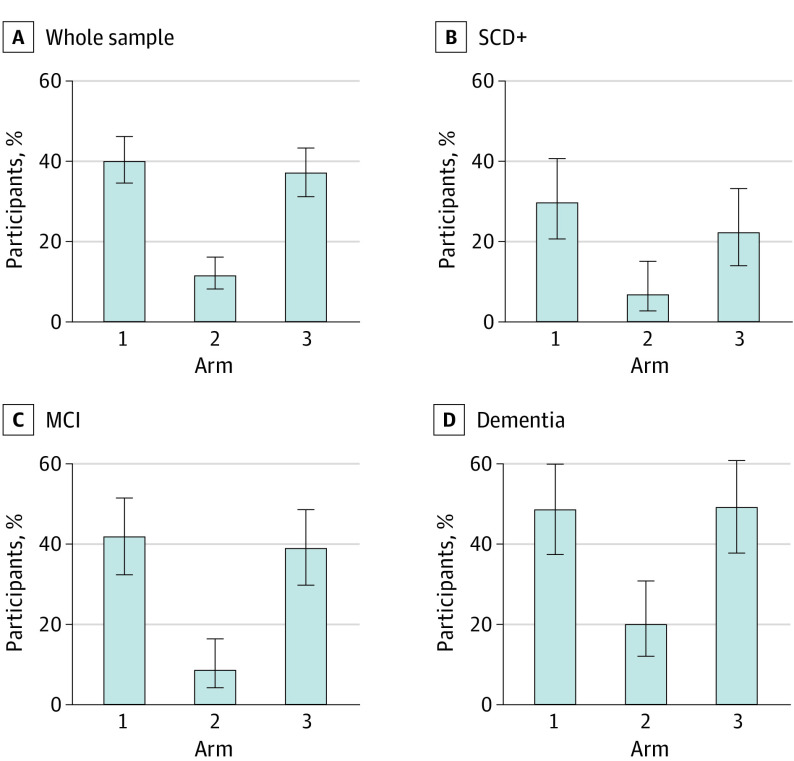
Proportions of Participants Receiving an Etiological Diagnosis With Very High Diagnostic Confidence (≥90%) After 3 Months MCI indicates mild cognitive impairment; SCD+, subjective cognitive decline plus clinical features increasing the likelihood of preclinical Alzheimer disease.

The proportion of participants with very high diagnostic confidence (≥90%) after 3 months was higher in arm 1 (109/272, 40%; 95% CI, 34%-46%; *P* < .001) than in arm 2 participants (30/260, 11%; 95% CI, 8%-16%) (Figure 3). The proportion was also higher in arm 3 (97/262, 37%; 95% CI, 31%-43%; *P* < .001) than in arm 2. Significant differences between arm 1 and arm 3 vs arm 2 were consistent across participants with SCD+ (arm 1: 25/84, 30%; 95% CI, 21%-41%; *P* < .001; arm 3: 17/77, 22%; 95% CI, 14%-33%; *P* = .03; vs arm 2: 5/78, 6%; 95% CI, 2%-15%), MCI (arm 1: 45/108, 42%; 95% CI, 32%-52%; *P* < .001; arm 3: 42/108, 39%; 95% CI, 30%-49%; *P* < .001; vs arm 2: 9/102, 9%; 95% CI, 4%-17%), or dementia (arm 1: 39/80, 49%; 95% CI, 38%-60%; *P* < .001; arm 3: 38/77, 49%; 95% CI, 38%-61%; *P* < .001; vs arm 2: 16/80, 20%; 95% CI, 12%-31%) (Figure 3).

### Change in Etiological Diagnosis After 3 Months

**Figure 4.**
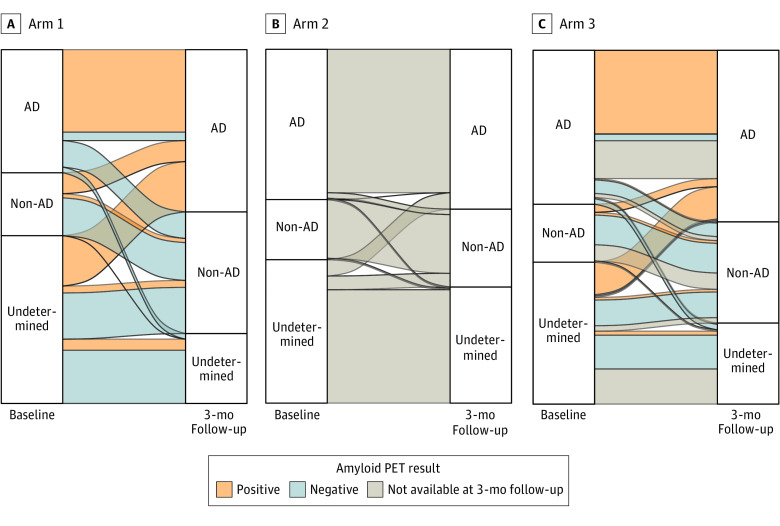
Change in Etiological Diagnosis After 3 Months in the Whole Sample AD indicates Alzheimer disease; PET, positron emission tomography.

Figure 4 and eFigures 2 and 3 in Supplement 1 illustrate how the baseline etiological diagnosis changed after 3 months. After 3 months, the proportion of participants changing etiological diagnosis was higher in arm 1 (112/253, 44%) than in arm 3 (77/262, 29%; *P* = .002) and arm 2 (28/259, 11%; *P* < .001) and in arm 3 than in arm 2 (*P* < .001). Participants with a baseline diagnosis of AD were reclassified more frequently in arm 1 (23/88, 26%; *P* < .001; always after negative amyloid PET) and arm 3 (19/114, 17%; *P* = .02) than in arm 2 (5/110, 5%). Participants with a baseline diagnosis of non-AD were reclassified more frequently in arm 1 (15/45, 33%; always after positive amyloid PET) than in arm 2 (1/44, 2%; *P* = .001). Finally, participants with an undetermined baseline diagnosis were reclassified more frequently in arm 1 (74/120, 62%; *P* < .001; after either positive or negative amyloid PET) and arm 3 (51/105, 49%; *P* < .001) than in arm 2 (22/105, 21%). Disaggregating by baseline cognitive stage (ie, SCD+, MCI, and dementia), we observed a trend consistent with that of the whole sample (eFigures 2 and 3 in Supplement 1). In arm 1 and arm 3, changes in the etiological diagnosis were far more consistent than inconsistent with the amyloid PET result (arm 1: 107/112, 96%, vs 5/112, 4%; *P* < .001; arm 3: 63/68, 93%, vs 5/68, 7%; *P* < .001) (Figure 4 and eFigure 3 in Supplement 1).

### Change in Diagnostic Confidence After 3 Months

In participants for whom a baseline etiological diagnosis of AD was confirmed after 3 months, diagnostic confidence increased more in arm 1 (+14%, from 71% to 85%; *P* < .001) and arm 3 (+11%, from 73% to 84%; *P* < .001) than in arm 2 (+1%, from 73% to 74%) (eFigure 4 in Supplement 1). In participants for whom a baseline etiological diagnosis of non-AD was confirmed after 3 months, diagnostic confidence increased more in arm 1 (+12%, from 72% to 85%; *P* < .001) and arm 3 (+10%, from 70% to 80%; *P* = .004) than in arm 2 (+1%, from 71% to 72%) (eFigure 4 in Supplement 1). Disaggregating by baseline cognitive stage (ie, SCD+, MCI, and dementia), we observed a trend consistent with that of the whole sample (eFigure 5 in Supplement 1).

We also assessed change in diagnostic confidence after 3 months in participants for whom the baseline etiological diagnosis changed and observed no differences across arms (eFigure 6 in Supplement 1); we reported the distribution of diagnostic confidence both at baseline and after 3 months (eFigure 7 in Supplement 1).

### Change in Cognition-Specific Medications After 3 Months

Change in cognition-specific medications was similar in the 3 study arms: 37 of 253 participants (15%) in arm 1 changed medications, 36 of 259 (14%) in arm 2, and 38 of 262 (15%) in arm 3 (*P* = .97) (eFigure 8 in Supplement 1). In analysis by baseline cognitive stage, no statistically significant differences among study arms were observed (eFigures 9 and 10 in Supplement 1).

In arm 1 and arm 3, change in cognition-specific medications occurred more frequently after positive rather than negative amyloid PET (arm 1: 31/37, 84%, vs 6/37, 16%; *P* < .001; arm 3: 24/27, 89%, vs 3/27, 11%; *P* < .001).

## Discussion

In this randomized clinical trial, we demonstrated that performing amyloid PET early in the diagnostic workup allowed 40% of memory clinic patients to receive an etiological diagnosis with very high confidence after only 3 months, 3.5 times more frequently than patients who had not yet undergone amyloid PET (11%). The major clinical impact of an early amyloid PET has been further confirmed by secondary analyses on changes in etiological diagnosis and diagnostic confidence. Interestingly, a significant clinical effect of amyloid PET was consistently observed across the 3 cognitive stages, suggesting that amyloid PET is clinically useful not only in MCI (ie, the population considered to benefit the most from biomarker assessment^20^), but also in patients at an early (SCD+) or advanced (dementia) cognitive stage. However, we did not observe a significant effect of amyloid PET on the prescription of cognition-specific medications, which were not initiated most of the time (their use is off-label in patients without AD dementia). Moreover, arm 3 (the free-choice arm) allowed us to assess the unrestricted use of amyloid PET in memory clinic patients. We observed that, in arm 3, amyloid PET was requested quite early in the diagnostic workup, mainly due to diagnostic uncertainty, a median 1.5 months from baseline, denoting a positive attitude among clinicians toward the technique and knowledge of its etiological use. Indeed, the clinical impact of amyloid PET in arm 3 is overall similar to that of arm 1 (early amyloid PET) and considerably higher than that of arm 2 (late amyloid PET).

A timely high-confidence diagnosis is critical to the efficacy of disease-modifying therapies, especially anti-amyloid drugs, whose efficacy might decrease with advancing disease progression. With the advent of disease-modifying therapies, amyloid PET might be used as a mere gateway to treatment, with the consequence that diagnostic outcomes will be no longer relevant. However, it has been estimated that the proportion of the real-world memory clinic population for whom aducanumab will be indicated (if the label mirrors eligibility criteria of phase 3 clinical trials) ranges between 1% and 12%,^21,22^ suggesting that the value of amyloid PET will stay purely diagnostic in the vast majority of patients.

Consistent with the IDEAS study,^9^ we observed a significant clinical effect of amyloid PET in terms of changes in diagnosis and diagnostic confidence. However, AMYPAD-DPMS and IDEAS showed inconsistent results in respect to changes in use of cognition-specific medications after amyloid PET. This discrepancy may be due to the fact that acetylcholinesterase inhibitors are not widely prescribed for MCI in Europe whereas they are in the United States.

One of the unique features of AMYPAD-DPMS is the assessment of the clinical impact of amyloid PET in individuals with SCD+ in a randomized controlled fashion. We observed that 30% of arm 1 participants with SCD+ were amyloid positive. This prevalence is slightly higher than a recent estimate of amyloid positivity in individuals with SCD of similar age (27%).^23^ This difference might be due to the features defining SCD+ that increase the likelihood of preclinical AD in individuals with SCD.^24^ Even though, according to the International Working Group clinical diagnostic criteria of AD published in 2021, a specific AD phenotype is necessary to make a clinical diagnosis of AD, 18 of 239 AMYPAD-DPMS participants with SCD+ (8%) received a baseline etiological diagnosis of AD. Moreover, participants with SCD+ did feature changes in etiological diagnosis and diagnostic confidence due to amyloid PET, suggesting that amyloid PET might be useful also in individuals without cognitive impairment and that future evidence-based appropriate use criteria might recommend the use of amyloid PET in this population, all the more so since it has been demonstrated that the disclosure of a positive amyloid PET result to patients with SCD+ was associated with a bigger psychological change, although such change did not reach the threshold for clinical concern.^25^ These results further underline the relevance of developing standardized assessment protocols for this relatively novel population, which is currently discharged by most memory clinics with generic recommendations and reassurance but without meaningful and actionable answers.^1,26^

It is worth noting that cerebrospinal fluid analysis might provide information similar to that provided by amyloid PET at a remarkably lower cost and can provide additional information on other biomarkers (eg, phosphorylated tau or neurofilament light). Nevertheless, amyloid PET is less invasive and better accepted by patients (indeed, in arm 3, 11% of participants explicitly wanted to undergo an amyloid PET, and 5% underwent amyloid PET because they refused lumbar puncture), and it is the biomarker of choice when lumbar puncture is contraindicated. Moreover, recent evidence shows that a dual-phase acquisition of amyloid PET imaging can also offer information on cortical perfusion (a proxy of metabolism and thus a measure of neurodegeneration) when additional early-phase images are acquired after tracer injection.^27^

### Limitations

The main limitation of the present study is the lack of health-related outcomes (eg, preventing death and disability, restoring or maintaining health and well-being, improving quality of life because of amyloid PET). Such outcomes are difficult to operationalize given the complexity and duration of AD and other neurodegenerative diseases and often require long-term follow-up, making the assessments expensive and logistically complicated.^28^ Because of these limitations, most studies focus only on proxies, ie, variables that are easier to assess (eg, changes in diagnosis, diagnostic confidence, and treatment plan) and possibly related to proper health-related outcomes.^28^ Exceptions are IDEAS and AMYPAD-DPMS. Indeed, IDEAS assessed hospitalizations and emergency-department visits as health-related outcomes, but their results have not yet been published. In AMYPAD-DPMS, we also collected health-related outcomes, and future studies will assess whether amyloid PET had an effect on them.

We acknowledge that the AMYPAD-DPMS sample mostly represents an academic memory clinic population, thus limiting the generalizability of our findings to nonacademic settings. Moreover, as 97% of our sample consisted of White patients, our study results might not be generalizable to different memory clinic populations with diverse race or ethnicity. Finally, in order to assess the study end points, we performed several statistical tests (in which pairwise comparisons were adjusted using Bonferroni correction, when applicable), amplifying the probability of false-positive findings.

## Conclusion

In this study, early amyloid PET allowed memory clinic patients to receive an etiological diagnosis with very high confidence after only 3 months compared with patients who had not undergone amyloid PET. This evidence from AMYPAD-DPMS of the clinical effect of amyloid PET in a European memory clinic population suggests that widespread implementation of this imaging technique may improve the timely diagnostic workup of patients under evaluation for cognitive decline.

## Notes

Supplement 1.
eMethods
**eTable 1**. Baseline features of the AMYPAD-DPMS participants disaggregating by baseline cognitive stage **eTable 2**. Etiological diagnoses at baseline and 3-month follow-up of the 794 AMYPAD-DPMS participants included in the main outcome analysis, disaggregating by baseline cognitive stage **eTable 3**. Diagnostic exams performed within 3 months, disaggregating by study arm **eResults**. Information on the managing physicians involved in the clinical assessment of the AMYPAD-DPMS participants **eTable 4**. Adverse events throughout the AMYPAD-DPMS study **eTable 5**. Reasons for requesting an amyloid-PET in ARM3 participants who underwent amyloid-PET **eFigure 1**. Prevalence of amyloid-PET positivity across cognitive stages and etiological diagnoses **eFigure 2**. Change in etiological diagnosis after 3 months, disaggregating by baseline cognitive stage **eFigure 3**. Change in etiological diagnosis after 3 months in participants with a baseline diagnosis of AD, non-AD, or undetermined, in the whole sample and disaggregating by baseline cognitive stage: comparison among arms **eFigure 4**. Change in diagnostic confidence after 3 months in participants with confirmed etiological diagnosis (whole sample) **eFigure 5**. Change in diagnostic confidence after 3 months in participants with confirmed etiological diagnosis, disaggregating by baseline cognitive stage **eFigure 6**. Change in diagnostic confidence after 3 months in participants with disconfirmed etiological diagnosis (whole sample) **eFigure 7**. Distribution of diagnostic confidence both at baseline and after 3 months **eFigure 8**. Change in cognition-specific medications after 3 months in the whole sample **eFigure 9**. Change in cognition-specific medications after 3 months, disaggregating by baseline cognitive stage **eFigure 10**. Change in cognition-specific medications after 3 months, in the whole sample and disaggregating by baseline cognitive stage: comparison among arms
eReferences

Supplement 2. Trial protocol

Supplement 3. Members of the AMYPAD Consortium

Supplement 4. Data sharing statement
