# Supplementary material for: Clinical Effect of Early vs Late Amyloid Positron Emission Tomography in Memory Clinic Patients: The AMYPAD-DPMS Randomized Clinical Trial
Source: JAMA Neurol. 2023 May 8;80(6):548–57. doi: 10.1001/jamaneurol.2023.0997 (PMC10167601; doi:10.1001/jamaneurol.2023.0997)
Supplement: Supplement 1. — eMethods eTable 1. Baseline features of the AMYPAD-DPMS participants disaggregating by baseline cognitive stage eTable 2. Etiological diagnoses at baseline and 3-month follow-up of the 794 AMYPAD-DPMS participants included in the main outcome analysis, disaggregating by baseline cognitive stage eTable 3. Diagnostic exams performed within 3 months, disaggregating by study arm eResults. Information on the managing physicians involved in the clinical assessment of the AMYPAD-DPMS participants eTable 4. Adverse events throughout the AMYPAD-DPMS study eTable 5. Reasons for requesting an amyloid-PET in ARM3 participants who underwent amyloid-PET eFigure 1. Prevalence of amyloid-PET positivity across cognitive stages and etiological diagnoses eFigure 2. Change in etiological diagnosis after 3 months, disaggregating by baseline cognitive stage eFigure 3. Change in etiological diagnosis after 3 months in participants with a baseline diagnosis of AD, non-AD, or undetermined, in the whole sample and disaggregating by baseline cognitive stage: comparison among arms eFigure 4. Change in diagnostic confidence after 3 months in participants with confirmed etiological diagnosis (whole sample) eFigure 5. Change in diagnostic confidence after 3 months in participants with confirmed etiological diagnosis, disaggregating by baseline cognitive stage eFigure 6. Change in diagnostic confidence after 3 months in participants with disconfirmed etiological diagnosis (whole sample) eFigure 7. Distribution of diagnostic confidence both at baseline and after 3 months eFigure 8. Change in cognition-specific medications after 3 months in the whole sample eFigure 9. Change in cognition-specific medications after 3 months, disaggregating by baseline cognitive stage eFigure 10. Change in cognition-specific medications after 3 months, in the whole sample and disaggregating by baseline cognitive stage: comparison among arms eReferences [file jamaneurol-e230997-s001.pdf]

Supplemental Online Content

Altomare D, Barkhof F, Caprioglio C, et al; for the AMYPAD Consortium. Clinical effect of early vs late amyloid positron emission tomography in memory clinic patients: the AMYPAD-DPMS randomized clinical trial. *JAMA Neurol*. Published online May 8, 2023.
doi:10.1001/jamaneurol.2023.0997

eMethods

eTable 1. Baseline features of the AMYPAD-DPMS participants disaggregating by baseline cognitive stage

eTable 2. Etiological diagnoses at baseline and 3-month follow-up of the 794 AMYPAD-DPMS participants included in the main outcome analysis, disaggregating by baseline cognitive stage

eTable 3. Diagnostic exams performed within 3 months, disaggregating by study arm

eResults. Information on the managing physicians involved in the clinical assessment of the AMYPAD-DPMS participants

eTable 4. Adverse events throughout the AMYPAD-DPMS study

eTable 5. Reasons for requesting an amyloid-PET in ARM3 participants who underwent amyloid-PET

eFigure 1. Prevalence of amyloid-PET positivity across cognitive stages and etiological diagnoses

eFigure 2. Change in etiological diagnosis after 3 months, disaggregating by baseline cognitive stage

eFigure 3. Change in etiological diagnosis after 3 months in participants with a baseline diagnosis of AD, non-AD, or undetermined, in the whole sample and disaggregating by baseline cognitive stage: comparison among arms

© 2023 Altomare D et al. *JAMA Neurology.*

eFigure 4. Change in diagnostic confidence after 3 months in participants with confirmed etiological diagnosis (whole sample).

eFigure 5. Change in diagnostic confidence after 3 months in participants with confirmed etiological diagnosis, disaggregating by baseline cognitive stage

eFigure 6. Change in diagnostic confidence after 3 months in participants with disconfirmed etiological diagnosis (whole sample)

eFigure 7. Distribution of diagnostic confidence both at baseline and after 3 months

eFigure 8. Change in cognition-specific medications after 3 months in the whole sample

eFigure 9. Change in cognition-specific medications after 3 months, disaggregating by baseline cognitive stage

eFigure 10. Change in cognition-specific medications after 3 months, in the whole sample and disaggregating by baseline cognitive stage: comparison among arms

eReferences

This supplemental material has been provided by the authors to give readers additional information about their work.

eMethods

1. Information on the allocation procedure and allocation ratio.

For each cognitive stage group (i.e. SCD+, MCI, dementia; based on the screening cognitive stage), the AMYPAD-DPMS participants were allocated to arms using a Minimization method[1], where a random component was added to give higher allocation probabilities to arms selected in favor of reducing total imbalance, taking into account the following relevant covariate: site, age at screening, and education.

The participant allocation was implemented with a Python wrapper plug-in function to the IXICO TrialTracker data management system. This executes the necessary sub-processes to assign an allocation group to each new participant and triggers an email notification to the participant's clinical site with the allocation information.

The plugin performs allocation with the Minimization method,[1] where a random component was added to give higher allocation probabilities to arms selected in favor of reducing total imbalance. This is a stratified sampling approach which minimizes the imbalance in the participant groups with relation to a set of pre-defined factors. Allocation is performed by calculating the imbalance caused by assigning a new participant to a given group for each factor; the selected allocation group is one that minimizes these imbalances. This method also allows for participant allocation without prior knowledge of the number of participants to be enrolled.

The allocation procedure was implemented for each cognitive stage group (i.e. SCD+, MCI, dementia) individually. The allocation plug-in assigns each new participant that enters the study to an allocation group using the information provided at the participant's screening assessment. Site, age, and level of education were considered as relevant covariates and therefore taken into account in the allocation process, as well as the allocation status of previous participants who entered the study to achieve maximum possible balance among the allocation arms.

© 2023 Altomare D et al. *JAMA Neurology.*

2. Major inclusion and exclusion criteria.

Inclusion criteria: to be enrolled in the study, patients must have met all the following criteria.

- The patient could be of any sex, gender, race, or ethnicity.
- The patient must have had a complaint (reported by the patient or by the caregiver) of cognitive problems that are considered by the managing physician to be possibly due to AD.
    - The patient must have been entering a diagnostic workup for the cognitive complaint.
    - The managing physician must have felt that knowledge of the patient's brain amyloid status may increase diagnostic confidence and alter diagnosis and/or management.
    - In some centers, the patient may have received diagnostic workup before being screened for this study. The patient could be enrolled in the study; however, if assigned to ARM1 (i.e. early amyloid-PET), the results of that workup must not have been made available to the managing physician before the managing physician reviewed the result of the amyloid-PET scan.
- The patient must have satisfied the diagnostic criteria specific to SCD+ or MCI or dementia (see section 2.2 in the main text).
- The patient had undergone a dementia blood workup before amyloid-PET.
- The patient had undergone an MRI and/or CT scan (not older than 12 months) before amyloid-PET.
- The patient could complete all clinical visits according to the protocol.
- The patient could tolerate a 20-minute amyloid-PET scan.
- The patient (or a legal representative) had provided informed consent for study participation and data source verification. In case the patient is randomized to ARM1 (i.e. early amyloid-PET), a new informed consent should be signed before the second imaging session.
- If the patient had dementia, a study partner was available for the duration of the protocol.
- The patient wanted to know the amyloid-PET result.

Exclusion criteria: patients must have been excluded from participating in this study if they met any of the following criteria.

- The patient had another confirmed condition that can fully account for the cognitive impairment (neuroinflammatory, neuroinfective, or neurodegenerative disease; multiple sclerosis; genetic disorders; HIV; brain injuries; neurosurgery after-effects; major depressive episode; schizoaffective disorder; delusional disorder; delirium).
- The patient came to observation for reasons other than diagnosis (disability assessment for social aids, cognitive assessment for driving license, etc.).
- The patient had a previous amyloid-PET and/or had other AD biomarker workup (e.g. FDG-PET or CSF analysis) before screening. In some centers, the patient may have received diagnostic workup before being screened for this study. The patient could be enrolled in the study; however, if assigned to ARM1 (i.e. early amyloid-PET), the results of that workup must not have been made available to the managing physician before the managing physician reviewed the result of the amyloid-PET scan.
- The patient had a life-threatening unstable medical disease or psychiatric condition that could lead to difficulty in complying with the protocol.
- The patient was receiving an investigational pharmaceutical product or had participated in a clinical trial with an investigational pharmaceutical product within 30 days before screening and/or was administered a radiopharmaceutical within 10 radioactive half-lives before study drug administration in this study.
- The patient was a woman who was pregnant, planning to become pregnant, or lactating.

While other inclusion/exclusion criteria of MCI and dementia were based on their respective clinical diagnostic criteria,[2,3] those of the SCD+ were based on a modified version of the SCD-I Working group criteria,[4] of which the most relevant features are: age between 60 and 85 years, perceived decline in memory over time, SCD onset within the previous 5 years and duration >6 months, Mini-Mental State Examination (MMSE) between 27 to 30, exclusion of MCI, explicit concerns (worries) about the cognitive symptoms, and active seeking of consultation; as described in a previous paper.[5]

© 2023 Altomare D et al. *JAMA Neurology.*

3. Sample size determination.

For the main outcome analysis, a sample size of 300 per arm was estimated to yield more than 99% power to detect a difference in the proportion of etiological diagnoses with very high diagnostic confidence between ARM1 and ARM2, assuming a difference in proportion of 25% (i.e. the targeted effect size, expected proportions were set to 62.5% in ARM1 and 37.5% in ARM2) and 10% withdrawal of participants. For each cognitive stage stratum (SCD+, MCI, and dementia), a sample size of 100 per arm was estimated to yield 80% power to detect a difference in proportion between ARM1 and ARM2, assuming a difference in proportion of 25%, 10% withdrawal of participants, and Bonferroni multiple testing correction. A smaller but still statistically significant difference would have been interpreted as "statistically significant but with lower clinical relevance". ARM3 has the same sample size as ARM1 and ARM2 in order to ensure comparability among arms. These values were computed with the sampsi procedure in STATA 14SE.

The total target sample size (n=900) was not achieved mostly due to the impact the coronavirus disease 2019 (COVID-19) pandemic.

© 2023 Altomare D et al. *JAMA Neurology.*

eTable 1. Baseline features of the AMYPAD-DPMS participants disaggregating by baseline cognitive stage.

Features	By cognitive stage		
	SCD+ n=239	MCI n=318	Dementia n=237
**Sociodemographic**			
Age	69 (9)	72 (11)	74 (11)
Gender, males	57% (137)	56% (179)	50% (119)
Education, years	14 (6)	12 (5)	12 (6)
Ethnicity (white)	99% (212) [25]	96% (275) [33]	97% (214) [16]
**Mental status**			
MMSE	29 (2) [1]	26 (4) [3]	22 (6) [2]
History of anxiety	22% (52)	22% (70)	16% (39)
HADS Anxiety	6 (5) [4]	6 (6) [4]	6 (6) [7]
History of depression	31% (73)	29% (93)	28% (67)
Depression in the last 5 years	25% (45) [57]	29% (66) [89]	27% (48) [57]
HADS Depression	3 (4) [4]	4 (5) [4]	5 (5) [7]
**Dementia risk factors**			
Hypertension	41% (82) [38]	48% (118) [74]	60% (102) [66]
Body mass index	26 (5) [5]	26 (5) [3]	25 (6) [13]
Reported cardiovascular events	41% (99)	35% (110)	42% (99)
Reported head injury	17% (40)	9% (30)	14% (33)
Smoking	12% (29)	12% (37)	10% (24)
Alcohol abuse	5% (13)	3% (11)	4% (10)
Vitamin deficiency	7% (17)	16% (52)	13% (31)
**Self-sufficiency**			
Disabilities	6% (15)	8% (24)	8% (20)
Living in institution	0% (0)	0% (0)	2% (5)
Still working	21% (49)	14% (44)	8% (19)
**Drugs and patient management**			
Cognition-specific medications, ≥1	5% (11)	6% (20)	25% (59)
Other medications, n	3 (5)	3 (3)	3 (4)
Non-pharmacological interventions, n	12% (28)	13% (40)	22% (51)
**Etiological diagnosis at baseline**			
AD	8% (18)	45% (142)	67% (159)
Non-AD	26% (63)	16% (50)	8% (19)
Undetermined	66% (158)	40% (126)	25% (59)
**Diagnostic confidence at baseline, %**			
In AD etiological diagnoses	60 (10)	70 (20)	80 (12)
In non-AD etiological diagnoses	75 (15)	65 (28)	70 (10)

© 2023 Altomare D et al. *JAMA Neurology.*

The table illustrates the main sociodemographic and clinical features of the AMYPAD-DPMS participants included in the main outcome analysis (intention-to-treat analysis).

MMSE: Mini Mental State Examination. HADS: Hospital Anxiety and Depression Scale. SCD+: subjective cognitive decline plus. MCI: mild cognitive impairment. AD: Alzheimer's disease.

Values are medians (interquartile ranges) for continuous variables, or percentages (raw numbers) for categorical variables.

At baseline, cognitive stages and etiological diagnoses were based on clinical and cognitive assessment and MRI or CT.

[number in square brackets]: number of missing data.

© 2023 Altomare D et al. *JAMA Neurology.*

eTable 2. Etiological diagnoses at baseline and 3-month follow-up of the 794 AMYPAD-DPMS participants included in the main outcome analysis, disaggregating by baseline cognitive stage.

Etiological diagnoses by T00 cognitive stage group		T00				T03			
		SCD+ n=239	MCI n=318	Dementia n=237	*p*	SCD+ n=239	MCI n=318	Dementia n=237	*p*
**AD (total)**		8% (18)[c]	45% (142)[b]	67% (159)[a]	**<0.001**	15% (35)[c]	48% (154)[b]	75% (178)[a]	**<0.001**
	AD	6% (14)	39% (124)	53% (126)		11% (27)	42% (133)	61% (145)	
	AD mixed	2% (4)	6% (18)	14% (33)		3% (8)	7% (21)	14% (33)	
**Non-AD (total)**		26% (63)[a]	16% (50)[b]	8% (19)[c]	**<0.001**	38% (90)[a]	31% (100)[a]	12% (29)[b]	**<0.001**
	CVD	2% (4)	6% (18)	3% (8)		3% (8)	7% (22)	3% (8)	
	DLB	0% (0)	1% (2)	2% (5)		0% (0)	0% (0)	1% (3)	
	FTLD	1% (2)	1% (2)	0% (1)		0% (1)	1% (4)	3% (7)	
	Psychiatric disease	9% (21)	3% (10)	0% (1)		12% (29)	10% (33)	0% (1)	
	Aging	8% (20)	0% (0)	0% (0)		13% (30)	2% (7)	0% (0)	
	Other	7% (16)	6% (18)	2% (4)		9% (22)	11% (34)	4% (10)	
**Undetermined**		66% (158)[a]	40% (126)[b]	25% (59)[c]	**<0.001**	48% (114)[a]	20% (64)[b]	13% (30)[b]	**<0.001**

T00: baseline. T03: 3-month follow-up. AD: Alzheimer's disease. CVD: cerebrovascular disease. DLB: dementia with Lewy bodies. FTLD: frontotemporal lobar degeneration. Psychiatric diseases include e.g. anxiety and depression. Aging indicates that the cause of cognitive complain is due to age-related physiological mechanisms. Other causes include e.g. corticobasal degeneration, alcohol abuse, sleep disorder, normal pressure hydrocephalus, suspected non-Alzheimer pathology.

Values are percentages (raw numbers). Statistical analyses: test for equality of proportions for categorical variables. If significant, post hoc analyses consist of pairwise comparisons for proportions, *p*-values were adjusted for multiple comparisons using Bonferroni correction.

Post hoc comparisons: [a] > [b] > [c].

© 2023 Altomare D et al. *JAMA Neurology.*

eTable 3. Diagnostic exams performed within 3 months, disaggregating by study arm.

Exams	Performed within 3 months			
	ARM1	ARM2	ARM3	*p*-value
CSF	8% (22/272)	10% (27/260)	9% (24/262)	0.66
FDG-PET	4% (11/272)	2% (5/260)	6% (16/262)	0.05
Other exams (e.g. DaTscan or EEG)	1% (2/272)	1% (3/260)	1% (3/262)	0.86

CSF: cerebrospinal fluid. FDG-PET: [18F] fluorodeoxyglucose positron emission tomography.

© 2023 Altomare D et al. *JAMA Neurology.*

eResults. Information on the managing physicians involved in the clinical assessment of the AMYPAD-DPMS participants.

A total of 78 managing physicians were involved in the clinical assessment of the AMYPAD-DPMS participants (5 affiliated with University and University Hospital of Geneva, 17 with Amsterdam University Medical Center, location VUmc, or external non-academic partnering memory clinics, 15 with Centre Hospitalier Universitaire de Toulouse [CHUT] or external non-academic partnering memory clinics, 6 with Barcelonaβeta Brain Research Center, 11 with University of Cologne, 9 with University College London or external non-academic partnering memory clinics, 7 with Karolinska Institutet, and 8 with Centre Hospitalier Universitaire Vaudois). Among them, 36 were neurologists, 16 geriatricians, 18 psychiatrists, and 8 with double specialties (3 neurology and psychiatry, 2 neurology and geriatrics, 2 geriatrics and internal medicine, 1 neurology and neuropathology). Median (IQR) years of experience of these physicians was 10 (14) (information on years of experience was not available for 1 managing physician from CHUT).

© 2023 Altomare D et al. *JAMA Neurology.*

eTable 4. Adverse events throughout the AMYPAD-DPMS study.

Adverse events	Adverse events severity	Serious adverse events	Adverse events relationship with amyloid-PET
31	25 mild	25 no	14 unrelated: bruising (n=5), increase in the morning tension and headaches (n=1), nausea (n=1), falls (n=1), somnolence (n=1), red rash (n=1), pins and needles sensation (n=1), hematoma (n=1), ear infection (n=1), erythema (allergy) (n=1).
			9 possibly related: dizziness (n=2), bruising (n=1), tightness in chest (n=1), headache (n=1), diarrhea and heat on the scalp (n=1), nausea (n=1), lightheadedness (n=1), diarrhea (n=1).
			2 probably related: asthenia (n=1), diarrhea (n=1).
	2 moderate	1 no	1 possibly related: headache and erythema (face) (n=1).
		1 yes	1 unrelated: angio-coronaropathy (n=1).
	4 severe	4 yes	2 unrelated: viral pneumonia (n=1), hospitalization for back and chest pain (n=1).
			2 possibly related: atrial fibrillation (n=1), suicide attempt by carbon monoxide intoxication (n=1).

During the study, we observed 31 adverse events (21 mild, 2 moderate, and 4 severe), including 5 serious adverse events. Among them, 17 were unrelated, 12 possibly related, and 2 probably related to amyloid-PET. The two observed severe adverse events possibly related to amyloid-PET were atrial fibrillation and suicide attempt by carbon monoxide intoxication.

© 2023 Altomare D et al. *JAMA Neurology.*

eTable 5. Reasons for requesting an amyloid-PET in ARM3 participants who underwent amyloid-PET.

Reason categories	Reason	% n=243
Diagnostic uncertainty	Unclear diagnosis	60% (147)
	To prove AD	43% (105)
	To exclude AD	32% (78)
Participant's preferences	Patient wanted an amyloid-PET scan	11% (26)
	Patient refused lumbar puncture	5% (13)
Other	Lumbar puncture was contraindicated, not possible, or not indicated in SCD	6% (15)
	Participant's willingness to participate in scientific research	9% (22)
	Lumbar puncture failed	2% (5)
	Unclear external CSF results, patient did not want 2nd lumbar puncture	0% (1)

Reasons for requesting an amyloid-PET were collected for all ARM3 participants who underwent amyloid-PET.

More than one reason for each participant was possible.

AD: Alzheimer's disease. SCD: subjective cognitive decline. CSF: cerebrospinal fluid.

eFigure 1. Prevalence of amyloid-PET positivity across cognitive stages and etiological diagnoses.

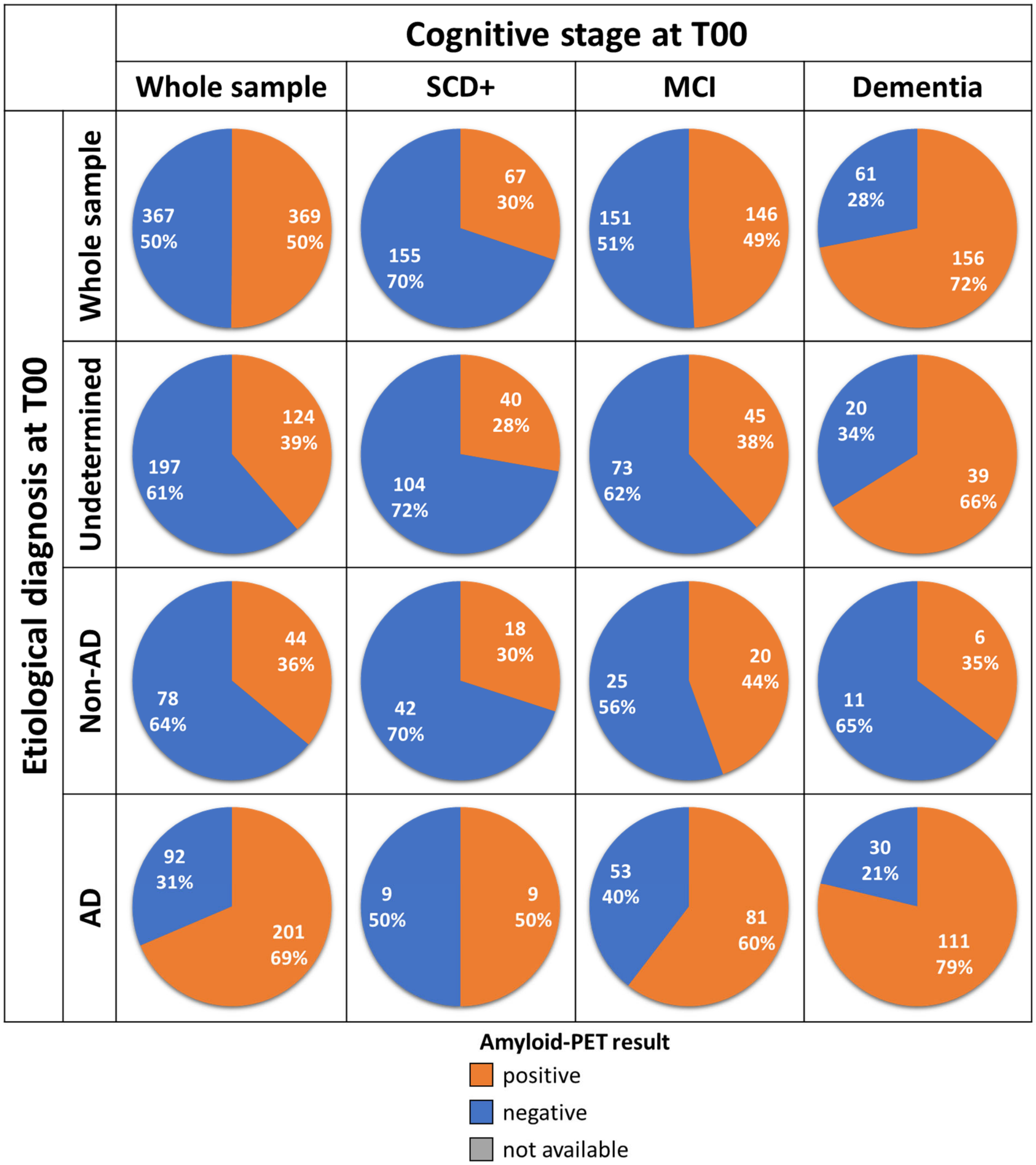

SCD+: subjective cognitive decline plus. MCI: mild cognitive impairment. AD: Alzheimer's disease. T00: baseline visit.

A total of 736 participants underwent amyloid-PET during the study course (384 with [18F]flutemetamol, and 352 with [18F]florbetaben, without considering the repeated amyloid-PET scans of ARM1 participants). They were amyloid positive in 50% (369/736) of cases. The prevalence of amyloid positivity increased with the severity of cognitive stage (p<0.001): 30% (67/222) in SCD+, 49% (146/297) in MCI, and 72% (156/217) in dementia; and in participants with a baseline diagnosis of AD (69%, 201/293) as compared to participants with non-AD (36%, 44/122, p<0.001) or undetermined diagnosis (39%, 124/321, p<0.001) at baseline.

© 2023 Altomare D et al. *JAMA Neurology.*

eFigure 2. Change in etiological diagnosis after 3 months, disaggregating by baseline cognitive stage.

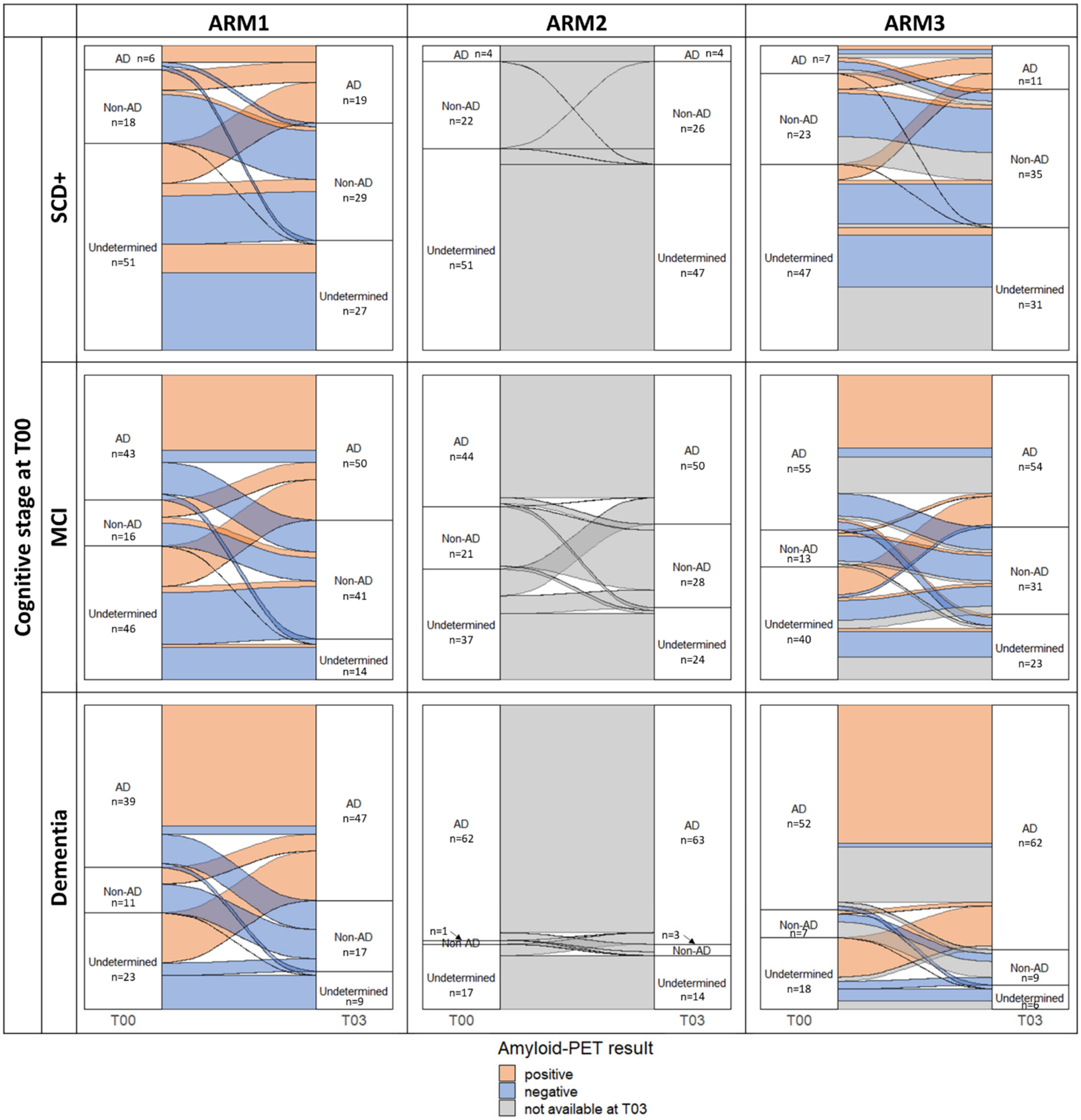

SCD+: subjective cognitive decline plus. MCI: mild cognitive impairment. AD: Alzheimer's disease. T00: baseline visit. T03: 3-month follow-up visit.

© 2023 Altomare D et al. *JAMA Neurology.*

eFigure 3. Change in etiological diagnosis after 3 months in participants with a baseline diagnosis of AD, non-AD, or undetermined, in the whole sample and disaggregating by baseline cognitive stage: comparison among arms.

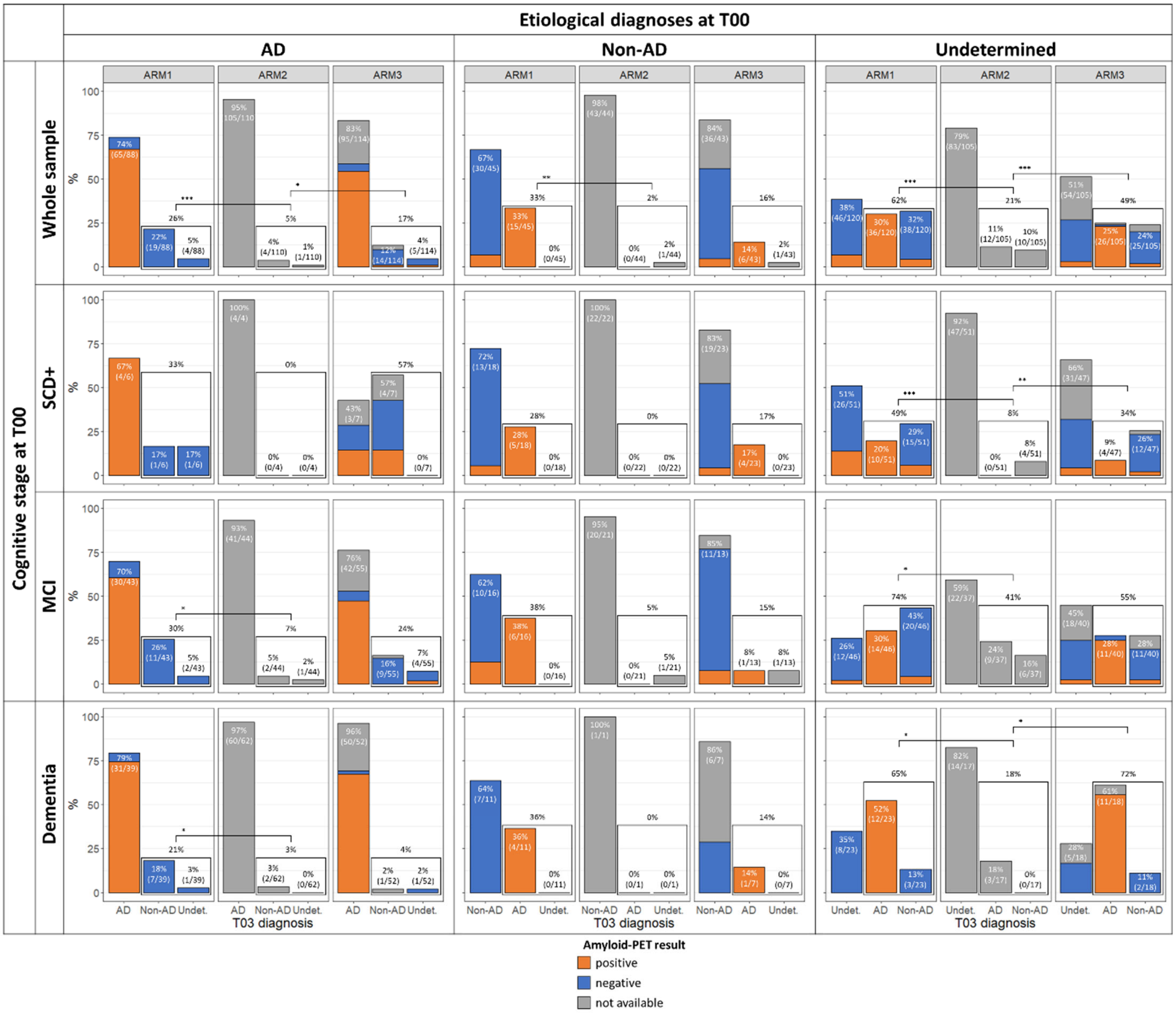

Bars indicate the frequency of etiological diagnoses after 3 months in the AMYPAD-DPMS participants disaggregating by baseline cognitive stage (i.e. SCD+, MCI or dementia) and baseline etiological diagnosis (i.e. AD, non-AD, or undetermined).

T00: baseline visit. T03: 3-month follow-up visit. ***: $p<0.001$. **: $p<0.01$. *: $p<0.05$.

Reading example: in participants with a baseline diagnosis of AD, the etiological diagnosis changed into non-AD or not yet achieved in 26% of cases in ARM1, 5% in ARM2, and 17% in ARM3, and the difference between ARM1 and ARM3 vs ARM2 is statistically significant ($p<0.05$).

© 2023 Altomare D et al. *JAMA Neurology.*

eFigure 4. Change in diagnostic confidence after 3 months in participants with confirmed etiological diagnosis (whole sample).

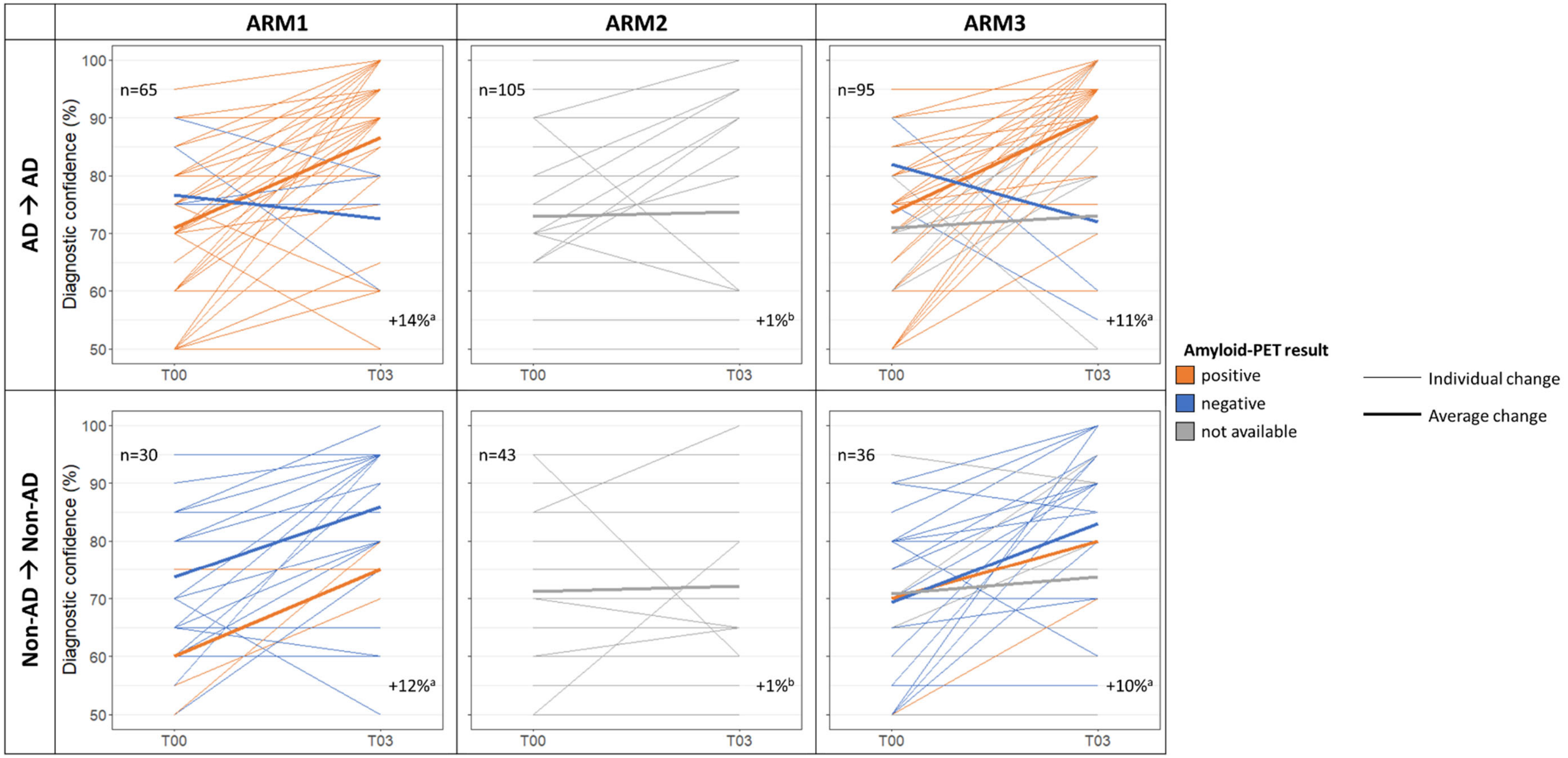

Diagnostic confidence (50-100%) was rated by managing physicians at baseline and after 3 months. Here we assessed changes in diagnostic confidence of participants with a confirmed diagnosis of AD ("AD → AD") or non-AD ("Non-AD → Non-AD").

AD: Alzheimer's disease. T00: baseline visit. T03: 3-month follow-up visit. Post-hoc comparison: [a] > [b].

© 2023 Altomare D et al. *JAMA Neurology.*

eFigure 5. Change in diagnostic confidence after 3 months in participants with confirmed etiological diagnosis, disaggregating by baseline cognitive stage.

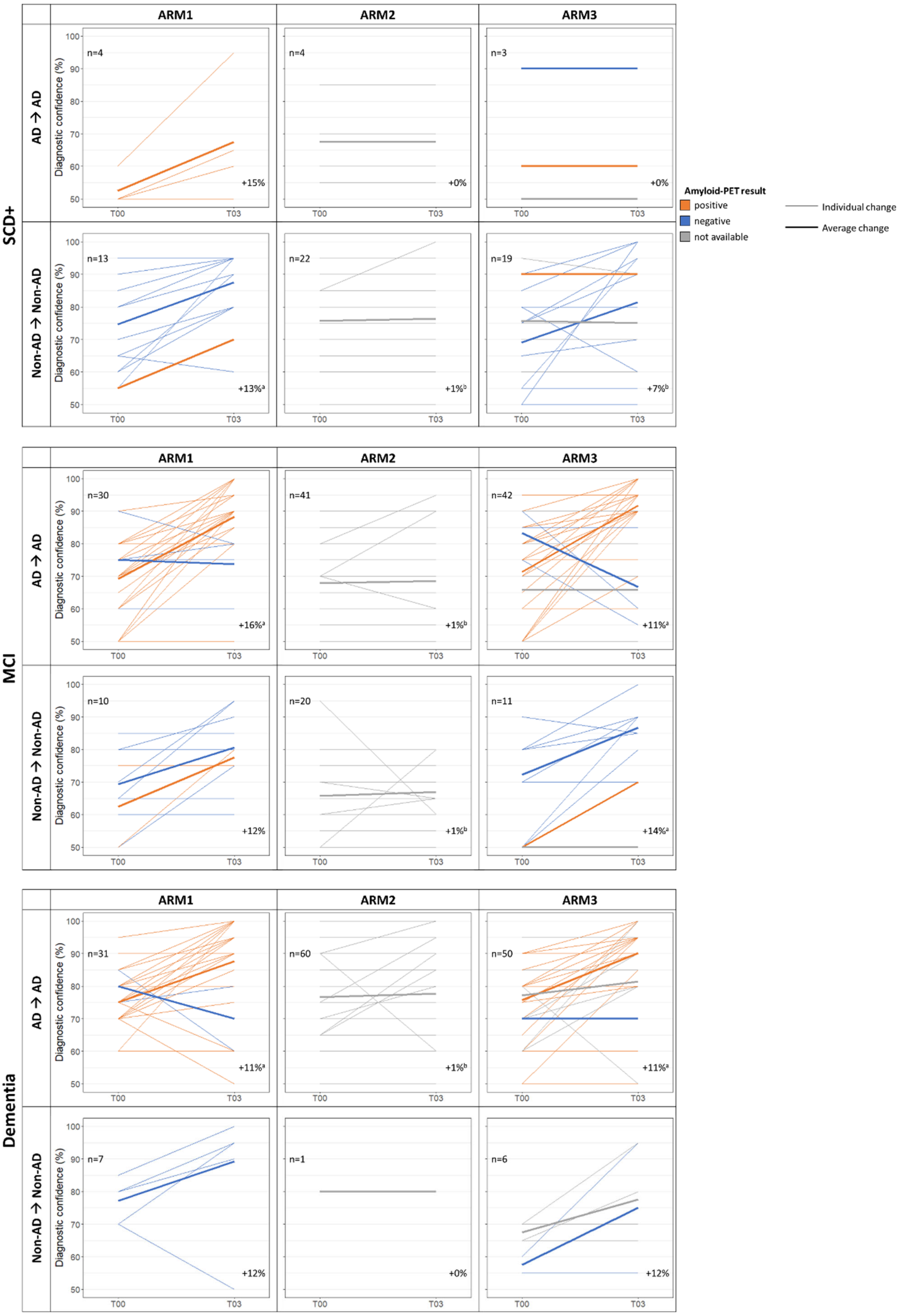

© 2023 Altomare D et al. *JAMA Neurology.*

Diagnostic confidence (50-100%) was rated by managing physicians at baseline and after 3 months. Here we assessed changes in diagnostic confidence of participants with a confirmed diagnosis of AD ("AD → AD") or non-AD ("Non-AD → Non-AD").

AD: Alzheimer's disease. T00: baseline visit. T03: 3-month follow-up visit.

Post-hoc comparison: [a] > [b].

© 2023 Altomare D et al. *JAMA Neurology.*

eFigure 6. Change in diagnostic confidence after 3 months in participants with disconfirmed etiological diagnosis (whole sample).

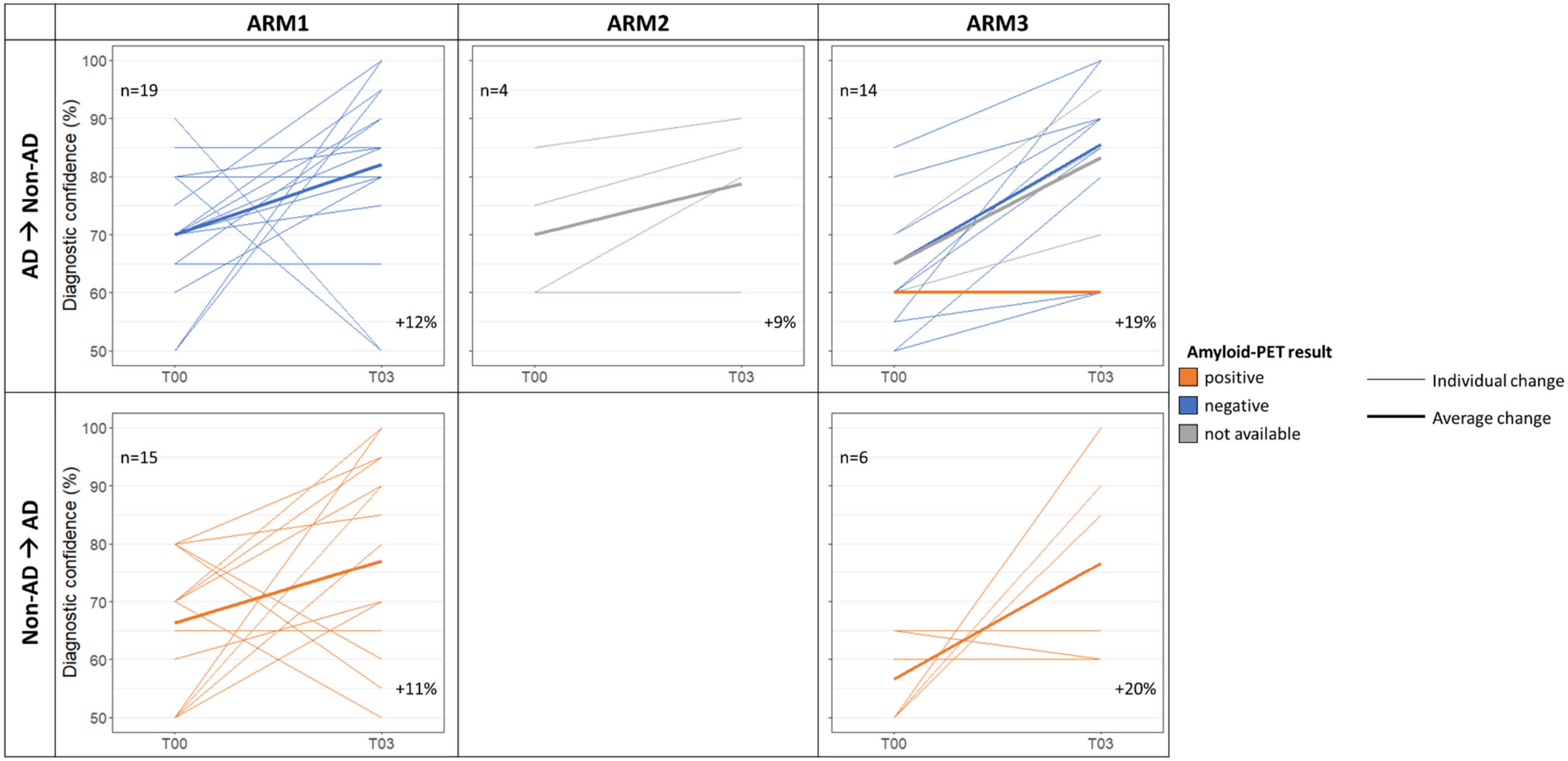

Diagnostic confidence (50-100%) was rated by managing physicians at baseline and after 3 months. Here we assessed changes in diagnostic confidence of participants with a changed diagnosis ("AD → Non-AD" or "Non-AD → AD").

AD: Alzheimer's disease. T00: baseline visit. T03: 3-month follow-up visit.

© 2023 Altomare D et al. *JAMA Neurology.*

eFigure 7. Distribution of diagnostic confidence both at baseline and after 3 months.

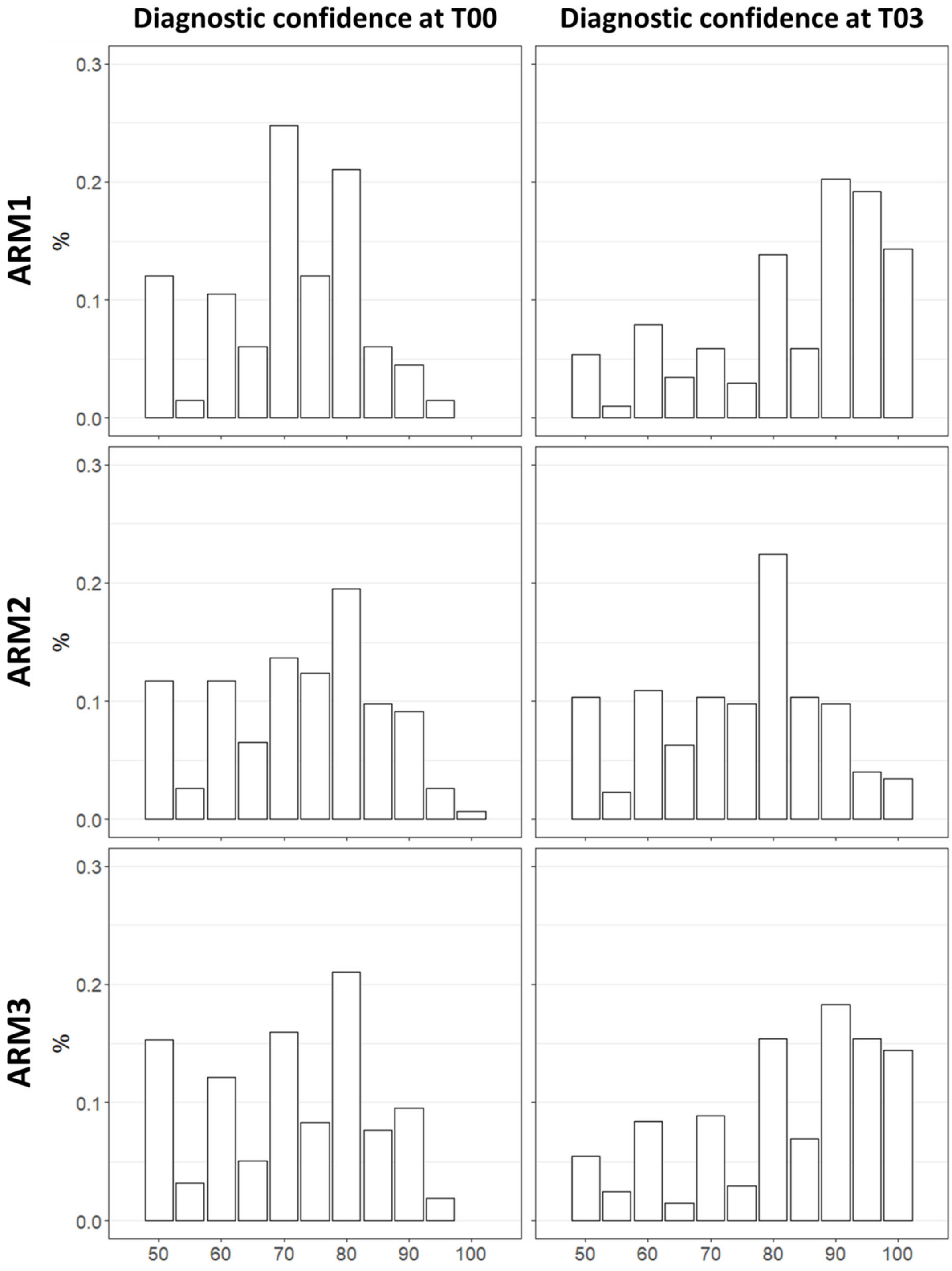

Diagnostic confidence (50-100%) was rated by managing physicians at baseline and after 3 months.

T00: baseline visit. T03: 3-month follow-up visit.

© 2023 Altomare D et al. *JAMA Neurology.*

eFigure 8. Change in cognition-specific medications after 3 months in the whole sample.

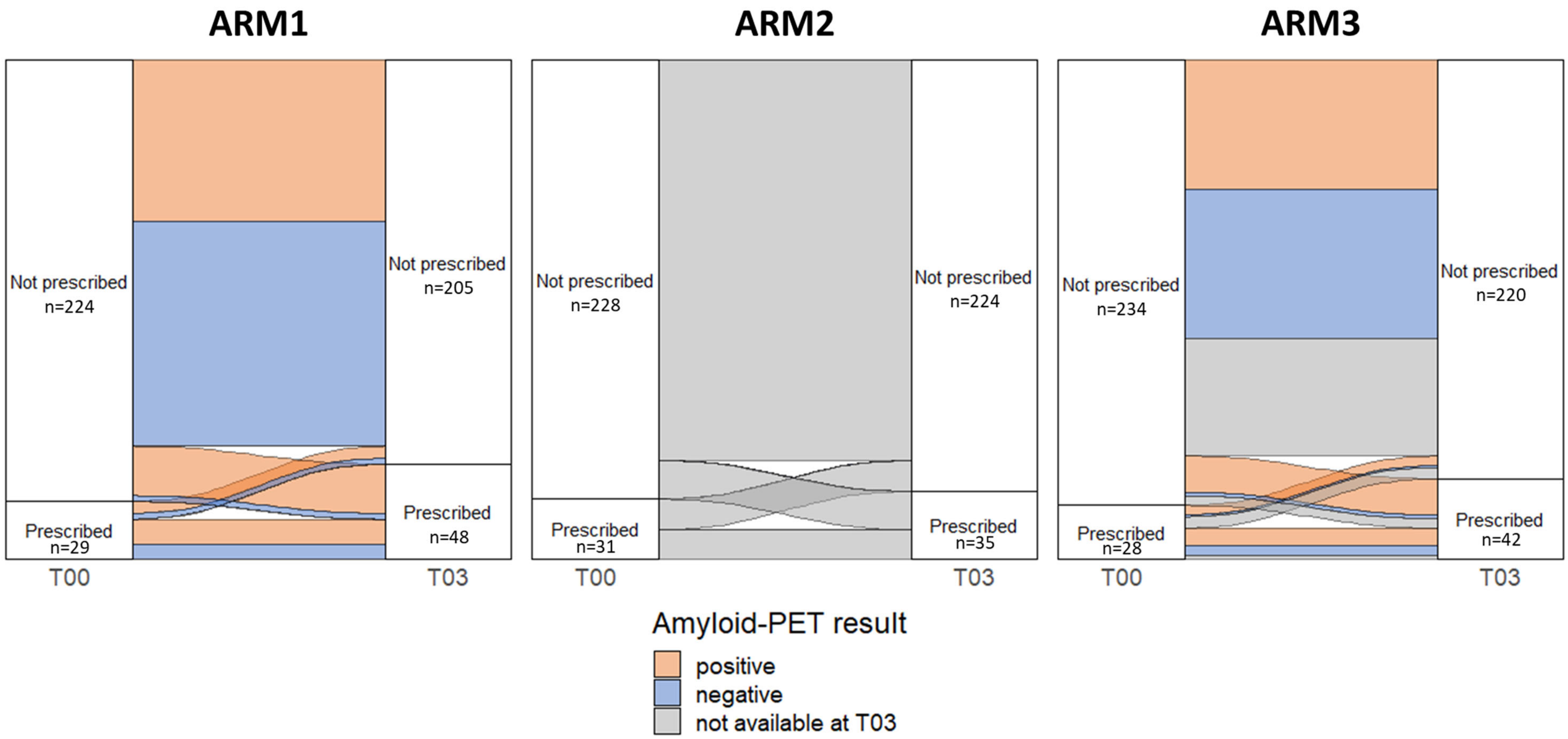

Cognition-specific medications include acetylcholinesterase inhibitors, memantine, Ginkgo, Souvenaid.

T00: baseline visit. T03: 3-month follow-up visit.

© 2023 Altomare D et al. *JAMA Neurology.*

eFigure 9. Change in cognition-specific medications after 3 months, disaggregating by baseline cognitive stage.

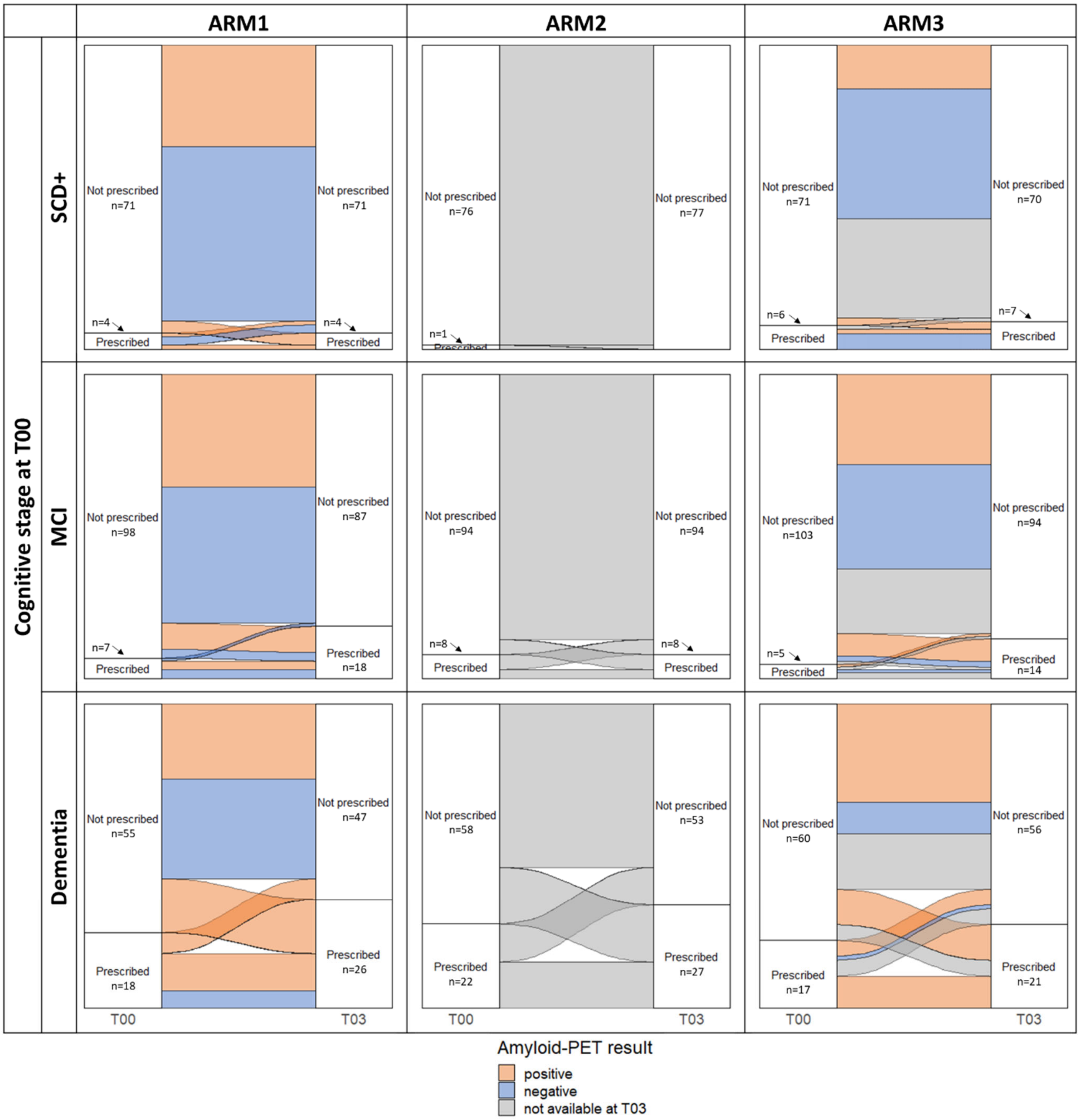

Cognition-specific medications include acetylcholinesterase inhibitors, memantine, Ginkgo, Souvenaid.

SCD+: subjective cognitive decline plus. MCI: mild cognitive impairment. T00: baseline visit. T03: 3-month follow-up visit.

© 2023 Altomare D et al. *JAMA Neurology.*

eFigure 10. Change in cognition-specific medications after 3 months, in the whole sample and disaggregating by baseline cognitive stage: comparison among arms.

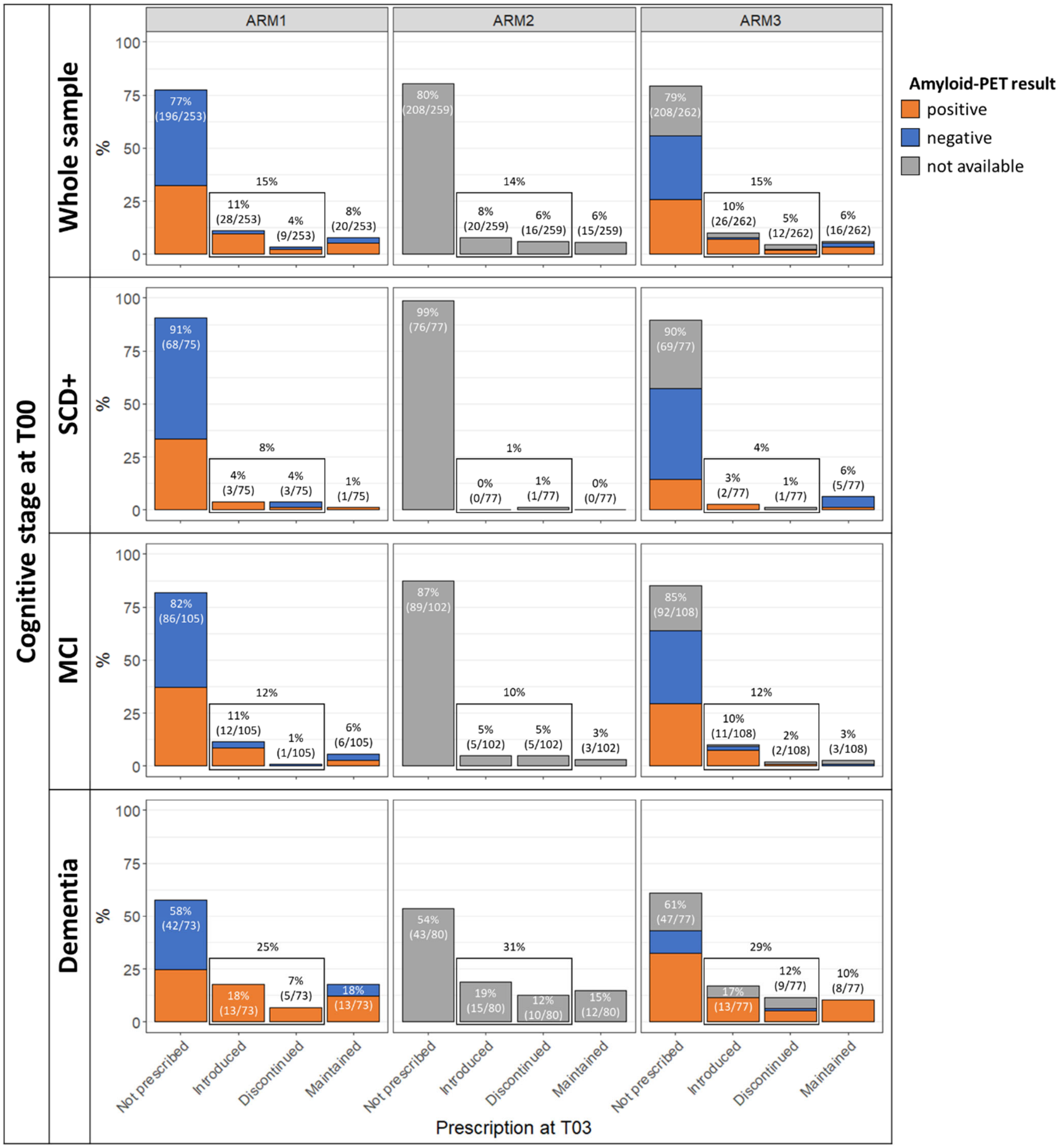

Cognition-specific medications include acetylcholinesterase inhibitors, memantine, Ginkgo, Souvenaid.

© 2023 Altomare D et al. *JAMA Neurology.*

Bars indicate the frequency of not prescribed, introduced, discontinued or maintained cognition-specific medications after 3 months in the AMYPAD-DPMS participants in the whole sample and disaggregating by baseline cognitive stage (i.e. SCD+, MCI or dementia).

SCD+: subjective cognitive decline plus. MCI: mild cognitive impairment. T00: baseline visit. T03: 3-month follow-up visit.

Reading example: in the whole sample, change in the prescription of cognition-specific medications occurred in 15% of ARM1, 14% of ARM2, and 15% of ARM3 participants, and the difference among arms is not statistically significant ($p>0.05$).

© 2023 Altomare D et al. *JAMA Neurology.*

eReferences

1. Saghaei, M. & Saghaei, S. Implementation of an open-source customizable minimization program for allocation of patients to parallel groups in clinical trials. *J. Biomed. Sci. Eng.* **4**, 734–739 (2011).

2. Albert, M. S. *et al.* The diagnosis of mild cognitive impairment due to Alzheimer's disease: Recommendations from the National Institute on Aging-Alzheimer's Association workgroups on diagnostic guidelines for Alzheimer's disease. *Alzheimer's Dement.* **7**, 270–279 (2011).

3. McKhann, G. M. *et al.* The diagnosis of dementia due to Alzheimer's disease: Recommendations from the National Institute on Aging-Alzheimer's Association workgroups on diagnostic guidelines for Alzheimer's disease. *Alzheimer's Dement.* **7**, 263–269 (2011).

4. Jessen, F. *et al.* A conceptual framework for research on subjective cognitive decline in preclinical Alzheimer's disease. *Alzheimer's Dement.* **10**, 844–852 (2014).

5. Frisoni, G. B. *et al.* AMYPAD Diagnostic and Patient Management Study: Rationale and design. *Alzheimer's Dement.* **15**, 388–399 (2019).

© 2023 Altomare D et al. *JAMA Neurology.*
