# Supplementary material for: Clinical Effect of Early vs Late Amyloid Positron Emission Tomography in Memory Clinic Patients: The AMYPAD-DPMS Randomized Clinical Trial
Source: JAMA Neurol. 2023 May 8;80(6):548–57. doi: 10.1001/jamaneurol.2023.0997 (PMC10167601; doi:10.1001/jamaneurol.2023.0997)
Supplement: Supplement 2. — Trial protocol [file jamaneurol-e230997-s002.pdf]

Clinical Study Protocol **University of Geneva**
AMYPAD-01 – Amendment 3

Title: Multicentre, Open-label, Randomised Study to Assess the Diagnostic Value of Amyloid PET Imaging in Patients with Subjective Cognitive Decline Plus, Mild Cognitive Impairment, or Dementia Where Alzheimer's Disease Is in the Differential Diagnosis (Diagnostic and Patient Management Study)

Sponsor

University of Geneva (hereinafter referred to as the "Sponsor") on behalf of AMYPAD Consortium

Rue Michel-Servet 1, 1206 Genève-CH

EudraCT NUMBER: 2017-002527-21

Study Director/Medical Monitor
Prof. Giovanni B. Frisoni, MD, PhD
University of Geneva
Rue Michel-Servet 1, 1206 Genève-CH
giovanni.frisoni@unige.ch

+41 (0)22 305 57 60

Confidentiality Statement

This protocol is provided for conducting a clinical research study. The information contained in this document is confidential and, except to the extent necessary to obtain informed consent or IEC/IRB approval, cannot be disclosed unless required by governmental regulation. Persons to whom any portion of the contents of this document is disclosed must be informed that the information is confidential and may not be further disclosed by them.

Investigator's Signature Page

I have read this protocol and all associated case report forms and agree to conduct this study in full accordance with the stipulations of the protocol described herein.

_______________________________ _______________________________

Signature Date

Giovanni B. Frisoni

____

Print Name

1 SYNOPSIS

**Name of Sponsor**<br>University of Geneva
**Title of Study:** Multicentre, Open-label, Randomised Study to Assess the Diagnostic Value of Amyloid PET Imaging in Patients with Subjective Cognitive Decline Plus, Mild Cognitive Impairment, or Dementia Where Alzheimer's Disease Is in the Differential Diagnosis (Diagnostic and Patient Management Study)
**Protocol Number:** AMYPAD-01
**Investigators and Study Centre(s):** At least 8 memory centres (and other satellite centres) in Europe
**Phase of Development:** Phase 4
**Expected Timeline:**<br>**First subject in:** 4Q2017; **End of study:** 4Q2020; **Final Clinical study report:** 3Q2021
**Purpose of the Study:**<br>This phase 4, multicentre, open-label, randomised study will explore the impact of amyloid PET imaging on diagnostic thinking in the workup of patients with SCD-plus (subjective cognitive decline associated with features that increase the likelihood of preclinical Alzheimer's disease [AD]), mild cognitive impairment (MCI), or dementia where AD is in the differential diagnosis.<br><br>**Primary Objectives:**<br>To test the hypothesis that the proportion of participants for whom the managing physician reaches an etiologic diagnosis with very high confidence (≥90%) at 12 weeks after baseline is higher for participants who underwent amyloid PET imaging shortly after baseline than for participants who have not yet undergone amyloid PET imaging (ie, participants scheduled to undergo amyloid PET imaging at 8 months [±8 weeks] after baseline). (According to [Johnson et al, 2013], the managing physician is a dementia expert trained and board-certified in neurology, psychiatry, or geriatric medicine who devotes a substantial proportion [≥25%] of patient contact time to the evaluation and care of adult-acquired cognitive impairment or dementia.)<br><br>**Secondary Objectives:**<br>***Diagnosis and Diagnostic Confidence***<br>To assess the impact of amyloid PET imaging on diagnosis-related metrics:<br>• Time to communicate to the patient an etiologic diagnosis with very high confidence (≥90%)<br>• Changes in the managing physician's etiologic diagnosis over time.<br>• Changes in the managing physician's diagnostic confidence over time.<br>• The managing physician's estimate of the likelihood that the patient's symptoms are due to AD.<br>• How the placement of amyloid PET imaging in the clinical workup, when the managing physician is given free choice, changes over time.<br><br>***Diagnostic/Therapeutic Management***<br>To assess the impact of amyloid PET imaging on patient management:<br>• Number of patients randomised to disease-modifying drug (DMD) or any other AD clinical trial at 6 months from baseline<br>• Change or early adoption of programs and/or pharmacologic treatments aimed to delay the onset or progression of cognitive impairment<br>• Use of medical resources (including but not limited to diagnostic procedures, tests, programs, visits, and hospitalisations)<br><br>***Health Economics and Patient-Centred Outcomes***<br>To assess the impact of amyloid PET imaging on:<br>• Patient-related outcomes (cognition, anxiety, depression, coping skills, and quality of life)<br>• Cost of diagnostic workup to the etiologic diagnosis with very high confidence (≥90%)<br>• Number of patients who are discharged from the memory centre and the reason for discharge<br><br>***Imaging results assessment***<br>• To test the hypothesis that amyloid load is stable over 12 to 18 months.

- To develop standardised methods of image quantitation across the PET tracers in order to allow pooled analysis across the AMYPAD program.

Study Design:

This is a phase 4 multicentre, open-label study of amyloid PET imaging of participants who have one of the following syndromic diagnoses: SCD-plus, MCI, or dementia where AD is in the differential diagnosis. At baseline, the investigator or a delegate will record the syndromic diagnosis made by the managing physician, the managing physician's confidence in that diagnosis, and the managing physician's estimate of the likelihood that the patient's symptoms are due to AD. (The investigator and the managing physician may be but do not have to be the same person.) Subjects will be stratified by syndromic diagnosis (SCD-plus, MCI, or dementia) and then randomly assigned (1:1:1) to 1 of 3 arms:

- **Early Amyloid PET Arm:** Participants will undergo amyloid PET imaging within 4 weeks after the baseline visit. Amyloid PET will be carried out with high priority over other diagnostic exams except MRI in the early amyloid PET arm. The results of the scan must be communicated to the managing physician as soon as available, and the etiological diagnosis as well as the diagnostic confidence must be collected immediately. Participants from the Early Amyloid PET Arm will undergo an optional second amyloid PET scan within a 12 to 18 months window after the initial scan.
- **Late Amyloid PET Arm:** Participants will undergo amyloid PET imaging at 8 months (±8 weeks) after baseline.
- **Free Choice Arm:** The managing physician will decide whether the participant undergoes amyloid PET imaging. The imaging can be done any time within 12 months after baseline. The reason for the choice will be collected.

In all Arms, between Baseline visit and Week 12 visit, each time a biomarker is available, the result must be communicated to the managing physician and the etiological diagnosis, as well as the diagnostic confidence, must be collected immediately. This will provide a sequential collection of diagnosis and diagnostic confidence up to Week 12 visit.

Amyloid load will be defined on the basis of visual reads at all time points. Only for the secondary aim of the disease modelling, amyloid load will be defined quantitatively. The results of the optional second amyloid PET
scans (early amyloid PET arm) will be provided to the managing physician and may be used for clinical purposes.
The goal will be to enrol approximately 100 participants per stratum in each arm, for a total of 900 participants.

Participation in the study will not prevent participants from having any currently available diagnostic investigation, but rather will give them the opportunity to have access to amyloid PET imaging, a diagnostic test approved by the European Medicines Agency but not yet reimbursed by most national health services.

The study will comprise the following clinical visits and timepoints:

- A baseline clinical visit (Visit 0 [VO] at Time 0 [T0]), within 14 days after screening
- A timepoint 12 weeks from baseline (T1); no clinic visit is required at that time
- A clinical visit at 6 months ±14 days (V1 or T2), and
- An optional clinical visit at 13 months ±4 weeks (V2 or T3)

Amyloid PET imaging (conducted at the local sites and read visually) will not be considered a separate clinical visit.

At T1, the investigator will review the result communicated by the managing physician (if the investigator and the managing physician are not the same person) and record the etiologic diagnosis made by the managing physician, rate the managing physician's diagnostic confidence (0% to 100%, visual analogue scale [VAS]) as well as the managing physician's estimate of the likelihood that the patient's symptoms are due to AD (0% to 100%, VAS), and record the effective date of the managing physician's etiologic diagnosis and management plan. This does not need to be a clinical visit.

At the baseline visit, all participants will receive a diary/questionnaire to collect information on the use of medical resources.

Selection of Participants:
All consecutive patients who come to a participating Memory Clinic with a request for diagnosis and are of the appropriate age will be considered for the study.
Inclusion Criteria:

- The patient can be of any sex, gender, race, or ethnicity.
- SCD-Plus patients must be 60 to 85 years of age
- Patients with MCI or dementia must be 50 to 85 years of age.
- The patient must have a complaint (reported by the patient or by a caregiver) of cognitive problems that are considered by the managing physician to be possibly due to AD.
  - The patient must be entering a diagnostic assessment for the cognitive complaint.
  - The managing physician must feel that knowledge of amyloid status may increase diagnostic confidence or alter diagnosis and management.
  - Patients should not have known amyloid status prior to randomization.
- The patient must satisfy the diagnostic criteria for one of the following:
  - SCD-Plus
  - MCI
  - Dementia, where AD is in the differential diagnosis
- The patient has undergone a dementia blood workup or will have one before amyloid PET.
- The patient has an MRI and/or CT scan (not older than 12 months) or will undergo one before amyloid PET.
- The patient can complete all clinical visits according to the protocol.
- The patient can tolerate a 20-minute amyloid PET scan.
- The patient (or, in case the patient is incapable of giving informed consent, next of kin or a legal representative as per national requirements.) provides informed consent for study participation and data source verification. In case of a change in the capacity to consent, the local regulations will be applied. In case the patient is randomized to the Early Amyloid PET arm, a new informed consent should be signed before the optional second imaging session.
- If the patient has dementia, a study partner is available for the duration of the protocol.

Exclusion Criteria:
Patient must be excluded if they meet any one of the following criteria:

- The patient has another confirmed condition that can fully account for the cognitive impairment, including but not limited to psychiatric disorders (schizophrenia, mood disorders, bipolar disorder and personality disorders); neuroinflammatory, neuroinfective, or neurodegenerative diseases; multiple sclerosis; genetic disorders; HIV; brain injuries; neurosurgery aftereffects, delirium). Patients with long-known, stabilized psychiatric or other brain conditions that cannot fully account for the cognitive impairment may be included in the study.
- The patient comes to observation for reasons other than diagnosis (disability assessment for social aids, cognitive assessment for driving license, etc.)
- The patient had a previous Aβ imaging scan and/or has had other AD biomarker workup (fluorodeoxyglucose [FDG]-PET and/or cerebrospinal fluid [CSF] analysis) prior to screening. In some centres, the patient may receive a diagnostic workup before screening. These patients can be enrolled if the investigator is blind to the results until after randomization.
- The patient has a life-threatening or unstable medical disease, or a psychiatric condition that could lead to difficulty in complying with the protocol.
- The patient is currently receiving an investigational pharmaceutical product or has participated in a clinical trial with an investigational pharmaceutical product within 30 days prior to screening, and/or was administered a radiopharmaceutical within 10 radioactive half-lives prior to study drug administration in this study.
- The patient is a woman who is pregnant, planning to become pregnant, or lactating. Pregnancy status of a woman with childbearing potential will be carried out before the PET scan. A woman is considered of childbearing potential (WOCBP), i.e. fertile, following menarche and until becoming post-menopausal unless permanently sterile (www.hma.eu/ctfg.html, Recommendations related to contraception and pregnancy testing in clinical trials, September 2014).

- The patient is employed at the research department or memory clinic, he/she is directly involved with the study, and it is a family relative from any department personnel (i.e. partner, older child, sibling, biological or legal representative).
- The patient is not affiliated to the Social security and does not receive the rights and corresponding coverage, in accordance with the Code of Public Health.
- In countries where patients unable of giving informed consent cannot be included in the study and an assessment of the patients' capacity to consent is requested, a study physician will assess the capacity to consent in patients with verifiable cognitive impairment prior to enrolment and regularly in the course of the study. If there are any doubts as to the patients' capacity to consent, the capacity to consent will be conclusively assessed by a specialist. Patients who are unable of giving informed consent, are excluded from the study in these countries.
- Any of the contraindications as registered for the study drug used is applicable to the subject. Any of the warnings or precautions as registered for the IMP used is applicable to the subject, unless a risk-benefit assessment is favorable as per the judgement of the sponsor.
-
-

Diagnostic Criteria:
During screening, the following criteria will be used for giving patients a syndromic diagnosis of SCD Plus, MCI, and dementia where AD is in the differential diagnosis.

Subjective Cognitive Decline Plus (SCD-Plus), modified from [Jessen et al, 2014]
The following features refer to SCD-Plus, in which the likelihood preclinical AD is increased:
- The patient is ≥60 years old.
- The patient has perceived a decline in memory over time.
- The onset of the cognitive decline is within the previous 5 years and the duration is >6 months
- The Mini-Mental State Examination® (MMSE®) score is 27 to 30 out of 30.
- The clinical examination and neuropsychological assessment exclude MCI.
- Cognitive decline has been confirmed by an informant.
- The patient (or caregiver) has expressed concerns (worries) about the cognitive symptoms.

Exclusion criteria for SCD-Plus:
- Current or past history of a neurologic disease with known potential impact on cognition.
- MRI lesions that would not be consistent with a diagnosis of AD.
- Current use of medication with known effect on cognition, including sedatives and drugs with anticholinergic effect, if the clinician believes that the use of those drugs is the cause of cognitive impairment.

Mild Cognitive Impairment (MCI) [Albert et al, 2011]
Diagnostic criteria for MCI are based on the National Institute on Aging and Alzheimer's Association (NIA-AA) Core Clinical Criteria:
- Concern regarding a change in cognition, as expressed by the patient, a proxy, or a physician.
- Impairment in one or more cognitive domains, as defined by neuropsychological test scores ≥1.5 SD below the age- and education-specific mean.
- Preservation of independence in functional abilities.
- No dementia.

Dementia where AD is in the differential diagnosis:
Probable AD Dementia
The diagnostic criteria for probable AD dementia are based on the NIA-AA core clinical criteria in addition to the criteria for dementia **[McKhann et al, 2011]**
- Insidious onset
- Clear-cut history of worsening of cognition by report or observation
- The initial and most prominent cognitive deficits are evident on history and examination

- Presentation can be amnestic or non-amnestic (language, visuospatial, executive function, etc)
- The diagnosis of probable AD dementia should not apply when there is evidence of substantial concomitant cerebrovascular disease, dementia with Lewy bodies, behavioural variant of frontotemporal dementia, semantic variant primary progressive aphasia or non-fluent/agrammatic variant primary progressive aphasia, evidence for another concurrent, active neurological disease, or non-neurological medical comorbidity or use of medication that could have a substantial effect on cognition.

Possible AD Dementia

The diagnostic criteria for possible AD dementia are based on the NIA-AA core clinical criteria in addition to the criteria for dementia [**McKhann et al, 2011**]:

- Atypical course (either a sudden onset of cognitive impairment or insufficient historical details or objective cognitive documentation of progressive decline).

Number of Participants Planned: 900 participants in European memory clinics.

Treatment of Participants:

Participants may receive [^{18}F]florbetaben or [^{18}F]flutemetamol for amyloid PET imaging as follows:

- **Early Amyloid PET arm**: Participants will undergo amyloid PET imaging within 4 weeks after baseline. The participants in this arm will have an optional second amyloid PET imaging scan 12 to 18 months (±28 days) after the first amyloid PET scan, with the same PET agent.
- **Late Amyloid PET arm**: Participants will undergo amyloid PET imaging 8 months (±8 weeks) after baseline.
- **Free Choice arm**: The managing physician will choose whether and when participants will have amyloid PET imaging at any time between baseline and 12 months after baseline.

In each imaging session, a single intravenous (IV) dose of amyloid PET tracer ([^{18}F]florbetaben or [^{18}F]flutemetamol) will be used:

- For [^{18}F]florbetaben, the recommended activity is 300 MBq (±20%).
- For [^{18}F]flutemetamol, the recommended activity is 185 MBq (±10%).

Efficacy, Health Economics, and Safety Variables

Primary Endpoint:

The difference, at 12 weeks after baseline, between the Early Amyloid PET arm and the Late Amyloid PET arm in the proportion of patients for whom the managing physician has made an etiologic diagnosis with very high confidence (≥90%). Diagnostic confidence will be measured with a visual analogue scale (VAS) ranging from 0% (no confidence) to 100% (full confidence).

Secondary Endpoints:

The timepoints are defined as follows: T0 = baseline, T1 = 12 weeks after T0, T2 = 6 months (±14 days) after T0, T3 = 13 months (±4 weeks) after T0; T4 = ≤28 days after the optional second scan, which will be 12 to 18 months after T0. (All time points are calculated relative to baseline.)

Diagnosis and Confidence

- The difference between the Early Amyloid PET arm and the Late Amyloid PET arm in the time (from baseline) to communicate a very-high-confidence (≥90%) etiologic diagnosis to the patient.
- Change of etiologic diagnosis and incremental diagnostic confidence between Baseline visit (T0) and T1 in each arm.
- Changes in the managing physician’s etiologic diagnosis at T3 vs T2 vs T1 in each arm.
- Changes in the managing physician’s diagnostic confidence at T3 vs T2 vs T1 vs T0 in the Early Amyloid PET arm vs the Late Amyloid PET arm.
- The managing physician’s estimate of the likelihood that the patient’s symptoms are due to AD at T3 vs T2 vs T1 vs T0 in the Early Amyloid PET arm vs the Late Amyloid PET arm.
- Changes over calendar time in the placement of amyloid PET imaging in the patient workup for participants in the Free Choice arm.

Patient Management

- The difference between arms (Early Amyloid PET arm, the Late Amyloid PET arm, or the Free Choice arm) in the number of patients randomised to disease-modifying drug (DMD) or any other AD clinical trial at T2.
- The difference between the Early Amyloid PET arm and the Late Amyloid PET arm in number of participants with changes in the management plan (changes in or start of a new program or pharmacologic treatment) at T1 vs T2 vs T3.

Health Economics
- The impact on patient-related outcomes (cognition, anxiety, depression, coping skills, and quality of life) at T3 vs T2 vs T0 in each arm
- The difference in the cost of diagnostic workup to the etiologic diagnosis with very high confidence (≥90%) in the Early Amyloid PET arm vs the Late Amyloid PET arm.
- Differences in the use of medical resources (not limited to diagnostic procedures, tests, visits, and hospitalisations) and programs between Early Amyloid PET and Late Amyloid PET arms.
- The number of patients who withdraw from the study.

Imaging Results Assessment
- Descriptive analysis of local visual assessment results
- Mean values of quantitative image assessments (composite cortical standardised uptake value ratios [SUVR] and converted to the centiloid scale) across amyloid PET tracers and by diagnostic subgroup.
- The composite cortical quantitative uptake (SUVR and SUVR converted to the centiloid scale) vs visual reading interpretation
- For each of the amyloid PET tracers, the differences in regional quantitative uptake between diagnostic strata.

For participants in the Early Amyloid PET arm who have a second amyloid PET scan:
- The shift from amyloid positive to amyloid negative, and vice versa.
- The difference in amyloid load, as indicated by quantitative image assessments, between the first and the second amyloid PET scan

Safety: Adverse events (AEs) that occur within 48 hours of study drug administration will be recorded. All serious AEs (SAEs) occurring within 30 days of study drug administration will be recorded.

A SCD-specific sub-study aiming at investigating the impact of PET-amyloid result disclosure will be performed on the SCD participants. This specific protocol is submitted separately as an AMYPAD DPMS sub-study.

Statistical Methods and Planned Analysis:

Populations for analysis:
Safety Analysis Set (SAS): All participants receiving any amount of either florbetaben or flutemetamol are included in the SAS.
Full Analysis Set (FAS): All participants randomized to the Early Amyloid PET, Late Amyloid PET, or Free Choice arm.
Per Protocol Set (PPS): All participants randomized to the Early Amyloid PET, Late Amyloid PET, or Free Choice arm with no major protocol deviations that would make them ineligible for the primary efficacy variable. Participants who are lost to follow-up will be excluded from the PPS.

For SAS, FAS, and PPS, demographic information (age, height, weight, and body mass index) will be summarised with descriptive statistics. Gender and race will be summarised by counts and percentages. Medical histories will be summarised by counts and percentages. Concurrent medications will be recorded and coded by using a standard classification system and grouped by primary and secondary classes, if applicable. The primary efficacy analysis will be based on the PPS population, and the secondary efficacy analysis will be based on the FAS population.

Primary Efficacy Analysis

- The difference between the Early Amyloid PET and Late Amyloid PET arms in the proportion of patients with an etiologic diagnosis in which the managing physician has ≥90% confidence at 12 weeks (the primary endpoint) will be evaluated with a chi-squared test with significance level of .05.
- For each of the three strata (SCD Plus, MCI, and dementia) separately, the difference between the Early Amyloid PET arm and the Late Amyloid PET arm in the proportion of patients with an etiologic diagnosis with a confidence ≥90% at 12 weeks will be evaluated with a chi-squared test with overall significance level of .05. The Bonferroni correction will be applied to control the family-wise type I error rate.
- Heterogeneity with respect to the effect on the primary variable between centres will be evaluated with the Breslow-Day test of homogeneity. If this effect varies across centres, centre-specific effects will be calculated.

Secondary Efficacy Analyses

- A difference between the Early Amyloid PET arm and Late Amyloid PET arm in the time to communicate an etiologic diagnosis with a confidence ≥90% will be tested by the log-rank test. The cumulative incidence of diagnoses will be estimated with the Kaplan-Meier method.
- A cross-sectional difference between arms in a secondary endpoint at T1, T2, T3, or T4 will be tested with a chi-squared test if the endpoint is dichotomous, a 2-sided *t*-test if the endpoint is normal, and a Mann-Whitney test if the endpoint is categorical or continuous and non-normal. The effect of baseline characteristics such as age, gender, MMSE, etc will be studied by a linear or logistic regression model.
- A difference between study arms in the longitudinal change of a secondary endpoint will be tested by means of logistic or linear regression where the first measurement is a covariate (ANCOVA analysis) and the second measurement is the outcome. Differences in longitudinal trends between arms will also be examined by means of the mixed-effects regression model. The effects of baseline characteristics such as age, gender, MMSE, etc will be studied by including them in the regression models.
- The Free Choice arm will be divided into 3 equal-sized groups, according to the date of randomisation. For the Free Choice arm, a change in the placement of amyloid PET imaging over time in the diagnostic workup will be tested by a log-rank test.
- For participants in the Early Amyloid PET arm who had a second amyloid PET scan, the difference in amyloid load between the first and the second amyloid PET scan, as indicated by quantitative image assessments, will be tested with a paired data *t*-test. Data will be log-transformed if non-normal.
- All tests will be 2-sided, and the significance level will be set at 5%. Bonferroni corrections for multiple testing will be applied when appropriate. Point estimates will be presented, together with 95% Wald confidence intervals. A 95% nonparametric confidence interval will be constructed if the estimate is non-normal.

Safety analysis:

- The number and percentage of participants with 1 or more AEs will be summarised for the Safety Analysis Set and separately by gender, age, study arms, and strata (SCD plus, MCI, and dementia) and by IMP ([^{18}F]florbetaben vs [^{18}F]flutemetamol).
- A difference among the 3 study arms and between Early and Late Amyloid PET arms in number of AEs at 48 hours after IMP administration will be tested by means of a chi-squared test with significance level of .05. The analysis will be repeated for the safety end-point SAEs at 30 days after IMP administration.

Sample Size Estimate

For the primary efficacy analysis, a sample size of 300 per arm yields more than 99% power when a difference in proportion of 25% is assumed and 10% of the patients are assumed to withdraw before the end-point is reached. For difference in proportion of values of 20%, 15%, and 10%, the power is 99.6%, 93%, and 61%, respectively. For each of the three strata (SCD Plus, MCI, and dementia), a stratum sample size of 100 per arm yields 80% power when the difference is 25% and 10% of the patients withdraw before the end-point is reached.

2 TABLE OF CONTENTS

2.1 Overall Table of Contents

Page Number

1 SYNOPSIS 3

2 TABLE OF CONTENTS 10

2.1 Overall Table of Contents 10
2.2 List of Tables 13
2.3 List of Figures 14

3 LIST OF ABBREVIATIONS AND DEFINITIONS OF TERMS 15

4 BACKGROUND INFORMATION 18

4.1.1 Utility of amyloid PET imaging in patient diagnosis and management18
4.1.2 Natural history of disease 19
4.1.3 AMYPAD Diagnostic and Patient Management Study –AMYPAD 1 20
4.1.4 Beta amyloid PET imaging 20
4.1.5 The burden of Alzheimer's disease 21

5 STUDY OBJECTIVES AND PURPOSE 22

5.1 Purpose of the Study 22
5.2 Research Hypothesis 22
5.3 Rationale for Conducting the Study 22
5.4 Objectives 22
5.4.1 Primary objectives 22
5.4.2 Secondary objectives 23

6 STUDY DESIGN 24

6.1 Overall Study Design and Plan 24
6.1.1 Study design 24
6.1.2 Follow-up amyloid PET Image visit 26
6.2 Study Rationale 27
6.3 Study Timeframe 27
6.4 Risks and Benefits to Participants 27
6.4.1 Potential risks 27
6.4.2 Potential benefits 28
6.4.3 Risk/benefit analysis 28

7 SELECTION AND WITHDRAWAL OF PARTICIPANTS 29

7.1 Procedures for Enrolment 29
7.2 Eligibility 29
7.3 Inclusion Criteria 29
7.4 Exclusion Criteria 30
7.5 Diagnostic Criteria 31

7.5.1 Subjective cognitive decline plus (SCD-Plus), modified from [Jessen et al, 2014] 31
7.5.2 Mild cognitive impairment (MCI) [Albert et al, 2011] 32
7.5.3 Dementia where AD is in the differential diagnosis: 32
7.6 Withdrawal and Termination Criteria 32
7.6.1 Participant withdrawal 32
7.6.2 Replacement of participants withdrawn from the study 33
7.6.3 Premature termination of study or site 33

8 TREATMENT OF PARTICIPANTS 34

8.1 Investigational Medicinal Products 34
8.1.1 NeuraCeq™ ([^{18}F]florbetaben) 34
8.1.2 Vizamyl™ ([^{18}F]flutemetamol) 35
8.1.3 Packaging and labelling 36
8.1.4 Drug supplies, logistics, and accountability 36
8.1.5 Drug logistics 36
8.2 Selection of Doses and Timing 37
8.2.1 Drug accountability 37
8.2.2 Dosage and administration of study drug 37
8.2.3 Measurement and documentation of the injected radioactivity: 38
8.2.4 Investigational Medicinal Product (IMP) accountability 38
8.2.5 Registration of investigational medicinal product(s) complaints 38
8.3 Method of Numbering Participants and Assigning Participants to Treatment Groups 39
8.3.1 Participant identification numbers (PIDs) 39
8.3.2 Randomisation 39
8.4 Selection of Doses and Timing 39
8.5 Blinding 40
8.6 Prior and Concurrent Therapy 40
8.7 Food Restrictions 40
8.8 Treatment of Study Drug Overdose 40
8.9 Treatment Compliance 40

9 STUDY PROCEDURES 41

9.1 Screening Period 44
9.2 Baseline Visit (V0 at T0) 45
9.3 Amyloid PET Imaging for Participants in Early Amyloid PET Arm (≤4 Weeks After Baseline) 46
9.4 T1, 12 Weeks After Baseline 46
9.5 V1 at T2, 6 Months (±14 days) After Baseline 47
9.6 Amyloid PET for Late Amyloid PET Arm, 8 Months (±8 Weeks After Baseline) 48
9.7 V2 at T3, 13 Months (±4 weeks) After Baseline 49
9.8 Study Completion 49
9.9 T4: Follow-up Visit for Participants in Early Amyloid PET Arm 50

10 EFFICACY, SAFETY, AND OTHER VARIABLES 51

10.1 Efficacy Assessments ... 51
10.1.1 Primary endpoint ... 51
10.1.2 Secondary endpoints ... 51
10.1.3 Image acquisition ... 52
10.1.4 Image storage ... 52
10.1.5 Image interpretation ... 53
10.1.6 PET data reporting ... 53
10.2 Safety Assessments ... 53
10.2.1 Treatment-emergent adverse events ... 53
10.2.2 Serious adverse events ... 54
10.2.3 Adverse event and serious adverse event reporting ... 55
10.2.4 Urgent safety measures ... 56
10.2.5 Pregnancy reporting ... 56
10.3 Other Variables ... 57
10.3.1 Demographic data ... 57
10.3.2 Medical and surgical history ... 57
10.3.3 Pre-treatment events ... 57
10.3.4 Prior and concurrent medication ... 58
10.3.5 Vital signs ... 58
10.3.6 Physical examination ... 58
10.3.7 Cognition/functioning ... 58
10.3.8 MRI and CT results ... 58
10.3.9 Mini-Mental State Examination ... 58
10.3.10 ICECAP-O (ICEpop CAPability measure for Older people) ... 58
10.3.11 Hospital Anxiety and Depression (HAD) ... 59
10.3.12 Patient questionnaire (use of medical resources) ... 59
10.3.13 Syndromic diagnosis ... 59
10.3.14 Etiologic diagnosis ... 59
10.3.15 Management plan ... 59
10.3.16 Diagnostic confidence ... 59
10.3.17 The managing physician’s estimate of the likelihood that the patient’s symptoms are due to AD ... 60
10.3.18 Amyloid PET scan results ... 60
10.3.19 FDG-PET ... 60
10.3.20 SPECT imaging ... 60
10.3.21 Cerebrospinal fluid (CSF) analysis ... 60
10.3.22 Blood tests ... 60
10.3.23 Optional neuropsychological testing ... 60
10.3.24 Other data ... 61
10.4 Appropriateness of Measurements ... 61

11 DATA HANDLING AND QUALITY ASSURANCE ... 62

11.1 Data Recording ... 62
11.2 Electronic Case Report Forms ... 62
11.3 Clinical Data Management ... 62
11.4 Data Processing ... 62
11.5 Archiving ... 62

11.6 Data Anonymization ... 63
12 STATISTICAL METHODS AND PLANNED ANALYSIS ... 64
12.1 General Statistical Considerations ... 64
12.2 Populations for Analysis ... 64
12.3 Participant Demographics/Other Baseline Characteristics ... 65
12.3.1 Physical examination findings ... 65
12.3.2 Vital signs ... 65
12.4 Study Treatments ... 65
12.5 Primary Analysis ... 65
12.5.1 Primary Efficacy Variables ... 65
12.5.2 Statistical hypothesis, model, and method of analysis ... 65
12.5.3 Handling of missing values/censoring/discontinuations ... 66
12.5.4 Supportive analyses ... 66
12.6 Secondary Analyses ... 66
12.6.1 Secondary efficacy variables ... 66
12.6.2 Safety variables and analyses ... 68
12.7 Sample Size Calculation ... 69
12.8 Procedures for Missing, Unused and Spurious Data ... 69
12.9 Interim Analysis ... 69
12.10 Rules for Excluding Participants from Analysis ... 69
12.11 Procedures for Reporting Deviations from Original Statistical Plan ... 69
13 SPECIAL REQUIREMENTS AND PROCEDURES ... 70
13.1 Regulatory, Institutional and Ethical Review ... 70
13.2 Ethical Considerations ... 70
13.3 Investigator’s Responsibilities ... 70
13.3.1 Overall responsibilities ... 70
13.4 Ethical and Legal Aspects ... 71
13.4.1 Ethical and legal conduct of the study ... 71
13.4.2 Participant informed consent ... 71
13.4.3 Direct access to source data/documents ... 72
13.4.4 Confidentiality regarding study participants ... 72
13.5 Protocol Deviations ... 73
13.6 Study Monitoring ... 73
13.7 Audit and Inspection ... 73
13.8 Financial Disclosure ... 74
13.9 Compensation for Health Damage of Participants / Insurance ... 74
13.10 Publication Policy ... 74
14 REFERENCES ... 75

2.2 List of Tables

Table 1. Study Schedule of Events ... 42

2.3 List of Figures

Figure 1 Study Diagram 26

3 LIST OF ABBREVIATIONS AND DEFINITIONS OF TERMS

Abbreviation	Description
Aβ	beta-amyloid
Aβ42	Amyloid-beta 42
AD	Alzheimer's disease
AE	Adverse event
AMYPAD	Amyloid Imaging to Prevent Alzheimer's Disease
ANCOVA	Analysis of covariance
Brief COPE	Brief Coping Orientation to the Problems Experienced
CJD	Creutzfeldt-Jacob Disease
CMP	Clinical Management Plan
CRO	Contract Research Organisation
CSF	Cerebrospinal Fluid
CT	Computerised tomography
CTCAE	Common Terminology Criteria for Adverse Events
DLB	Dementia with Lewy Bodies
DMD	Disease-modifying drug
eCRF	Electronic Case Report Form
EQ 5D 5L	EuroQol 5 dimensions questionnaire
FBB	Florbetaben
FDG-PET	Fluorodeoxyglucose positron emissions tomography
FLU	Flutemetamol
FTD	Frontotemporal dementia

Abbreviation	Description
GCP	Good Clinical Practice
GMP	Good Manufacturing Practice
HAD	Hospital Anxiety and Depression Scale
IB	Investigator’s Brochure
ICECAP-O	ICEpop CAPability measure for Older people
ICF	Informed Consent Form
ICH	International Conference on Harmonisation
ICRP	International Commission on Radiological Protection
IEC	Independent Ethics Committee
IRB	Institutional Review Board
i.v.	Intravenous
LP	Lumbar Puncture
MBq	Megabecquerel(s)
mCi	milliCurie(s)
MCI	Mild Cognitive Impairment
MMSE®	Mini-Mental State Examination
MRI	Magnetic resonance imaging
NIA-AA	National Institute on Aging and Alzheimer's Association
NOAEL	no-observed-adverse-effect-level
PET	Positron emission tomography
pTau	phosphorylated-Tau
SAE	serious adverse event
SAP	Statistical Analysis Plan
SCD	Subjective Cognitive Decline

Abbreviation	Description
PID	Participant Identification Number
SPECT	Single-photon emission computerised tomography
SUVR	Standardised uptake value ratio
tTau	total Tau
TMF	Trial Master File
VAS	Visual Analogue Scale

4 BACKGROUND INFORMATION

Alzheimer's disease (AD) is the most common cause of cognitive impairment and dementia and represents over 60% of all dementia cases [Silverman et al, 2004]. The key neuropathological hallmarks of AD include the presence of extracellular deposits of beta-amyloid (Aβ) peptides, intraneuronal neurofibrillary tangles, and the predominance of neocortical neuronal degeneration [Serrano-Pozo et al, 2011]. However, despite a clinical assessment, AD is often not recognised, particularly in the early stages or if mixed pathology is present.

Alzheimer's disease is a progressive disorder and the processes underlying the disease begin years if not decades before the onset of cognitive defects or dementia symptoms. There is no fixed event to define the onset of AD or to identify the point at which an individual transitions between phases [Albert et al, 2011]. Diagnosis based on clinical criteria alone is difficult. It is estimated that around 50% of diagnoses in persons presenting with objective cognitive impairment are incorrect. Most commonly, the impairment is attributed to Alzheimer disease. The percentage of incorrect diagnoses is still substantial even in more advanced stages of the disease – comparisons to a neuropathological standard of truth reveal that up to one-third of patients are misdiagnosed [Beach et al, 2012].

Beta-amyloid (Aβ) deposition is considered to be a necessary—but not sufficient—step on the path toward development of Alzheimer's disease (AD). Depiction of brain Aβ in vivo [Curtis et al, 2015][Sabri et al, 2015] can therefore improve an early diagnosis of AD, and, when recognised in a pre-symptomatic population, it might provide an opportunity for secondary prevention of dementia. Understanding the value of positron emission tomography (PET) imaging of Aβ provides a unique opportunity to achieve 2 major goals: 1) improve the diagnostic workup of patients suspected to have AD [Beach et al, 2012] and their management; 2) understand the natural history of AD in a pre-symptomatic stage in order to better select patients for trials.

Through engagement with regulators, the AMYPAD consortium will maximise the value of its findings for pharmaceutical companies, healthcare providers, and patients [Frisoni et al 2017].

4.1.1 Utility of amyloid PET imaging in patient diagnosis and management

The value, timing, and appropriateness of amyloid PET imaging in clinical practice need to be established to allow its cost-effective implementation in the diagnosis process [Hornberger et al, 2015] of cognitive decline and dementia. This study (AMYPAD Diagnostic and Patient Management Study 01) will determine in a real-life clinical setting for whom diagnostic amyloid PET imaging is appropriate, when this is best performed, and how the resulting information is influencing diagnostic confidence, patient management, and ultimately decision trees and cost-effectiveness of dementia care.

Specifically, we will study 3 groups of patients presenting to memory clinics with a cognitive impairment due to AD, specifically those with subjective cognitive decline plus (SCD Plus), mild cognitive impairment (MCI), and dementia, to determine when amyloid PET imaging helps to exclude AD aetiology (negative predictive value) or conveys an increased risk of AD

(positive predictive value) vis-à-vis other information and evidence (clinical, structural imaging, genetic, CSF). Ultimately, the AMYPAD Diagnostic and Patient Management Study will deliver an encompassing diagnostic algorithm allowing a cost-efficient implementation of amyloid PET imaging in the clinical practice.

We will study the impact of the information provided by amyloid PET imaging beyond diagnosis by determining the impact on patient management and health resource utilisation, both in the current era of symptomatic treatment and in the context of potentially effective disease-modifying therapies aimed at lowering Aβ in the brain. Furthermore, we will determine if our diagnostic algorithm is cost-effective from a health care perspective through providing the input of economic modelling, in the context of the current regulatory perspective.

4.1.2 Natural history of disease

Ageing is the leading risk factor for development of AD; the prevalence of dementia is 1 in 9 people age ≥65 years, rising to 1 in 3 people age ≥85; and more than half of these people have AD. Genetic, environmental, and comorbid illnesses also confer risk. Indeed, the genetic risk factors in familial, early-onset AD that have provided the strongest insight into the pathophysiology of the disease as they include mutations in the amyloid precursor protein and presenilin proteins. These mutations lead to increased turnover of Aβ and production of cleavage products such as $A\beta_{1\text{-}42}$, which are prone to misfolding and formation of extracellular aggregates.

Identification of fibrillary amyloid plaques in cortex is one of the requirements for the diagnosis of AD at autopsy. While the abundance of fibrillary amyloid plaque does not correlate with degree of cognitive impairment in life, preclinical studies suggest that the soluble oligomeric of Aβ, the intermediates to insoluble plaques, are neurotoxic and may trigger other neurotoxic cascades such as the spreading of phosphorylated tau, proteins that are also subject to misfolding and self-aggregation and form intracellular neurofibrillary tangles. This hypothesis is illustrated by the 'Jack' model of the progression of biomarkers in AD [Jack et al, 2010][Jack et al, 2013].

In the Alzheimer's Disease Neuroimaging Initiative (ADNI) studies from US and other countries and the Australian Imaging, Biomarker & Lifestyle Flagship Study of Ageing (AIBL), all of which evaluate groups of elderly subjects with normal cognition, cognitive impairment, and dementia as a pseudolongitudinal model of disease progression, the frequency of high brain amyloid burden as measured by amyloid PET imaging rises from around 30% in healthy elders to over 80% in AD subjects. Evidence of high brain amyloid burden, either by CSF analysis or by amyloid PET imaging, has been proposed to define the earliest stages of preclinical AD [Sperling et al, 2011].

Cross-sectional and longitudinal imaging studies using amyloid PET tracers in healthy adults have shown that amyloid signal can emerge during the 6th and 7th decade of life [Vlassenko et al, 2011]. Subsequent amyloid accumulation tends to be gradual, and the rate of accumulation plateaus once a subject has reached the AD stage; although small increases may still be observed and possibly decreases in late stage disease with advanced neurodegeneration [Jack et al, 2013].

4.1.3 AMYPAD Diagnostic and Patient Management Study

To identify the optimal window of opportunity for possible intervention in the Aβ pathway, researchers must establish in detail the sequence of events on the path towards AD.

This study will select and follow up a memory clinic population suspected of possible AD, focusing on those with subjective cognitive decline plus (SCD Plus), mild cognitive impairment (MCI), and dementia, to determine the utility of amyloid PET imaging with regards to change in diagnosis, diagnostic confidence, decision trees, and alterations between planned and actual patient management plans.

Through a European network of diagnostic memory clinics and affiliated PET centres, we will determine:

- Impact on diagnostic thinking and patient management
- Impact on diagnostic confidence and decision making
- Impact on patient reported outcomes
- Clinical utility of amyloid PET imaging in preclinical phases of AD (patients with SCD Plus)
- Costs and cost-effectiveness of early adoption vs late adoption of amyloid PET imaging
- Standardised methods of image quantitation across the PET tracers in order to perform pooled analysis across the AMYPAD program

4.1.4 Beta amyloid PET imaging

The development of radioligands to image Aβ plaques in vivo in the human brain is an important and active area of radiopharmaceutical research. When tracers of this kind are used in combination with PET imaging, the ability to image Aβ permits early and accurate differential diagnosis in cases of dementia.

Initially, the most widely used PET Aβ ligand in amyloid imaging research to assess the Aβ burden was ^{11}C Pittsburgh compound B (PiB). The first human studies commenced in 2002 [Rowe and Villemagne, 2013]. Following publication of results from this study in 2004, ^{11}C-PiB imaging spread rapidly to worldwide usage, and it led the way in Aβ imaging. However, a major disadvantage of ^{11}C-PiB was the 20-minute radioactive decay half-life of ^{11}C, which limited its use to centres with an on-site cyclotron and ^{11}C radiochemistry expertise [Villemagne et al, 2011]. It also therefore restricted its use in routine clinical research. To overcome these limitations, a number of novel tracers labelled with fluorine-18 were synthesised and tested. The half-life of fluorine-18 is 110 minutes, which allows centralised production and regional distribution of these tracers, as currently practiced worldwide in the supply of [^{18}F]fluorodeoxyglucose for routine clinical PET imaging in oncology [Ong et al, 2013][Villemagne et al, 2011]

Two novel fluorine-18 tracer compounds, approved in the US and Europe, [^{18}F]florbetaben (trans-4-(N-methyl-amino)-4"(2-(2-(2-[^{18}F] fluoro-ethoxy)ethoxy)-ethoxy)stilbene) by Piramal Imaging, and [^{18}F]flutemetamol (FLU; 2-[3-[^{18}F]fluoro-4-(methylamino)phenyl]-1,3-benzothiazol-6-ol) by GE Healthcare Life Sciences will be utilised within this protocol

4.1.5 The burden of Alzheimer's disease

AD is the most common aetiology of dementia. The typical clinical presentation is progressive loss of memory and other areas of cognitive function together with functional impairment, ultimately leading to dependency and death.

Today, over 46 million people live with dementia worldwide. This number is estimated to increase to 131.5 million by 2050. Dementia also has a huge economic impact. The total estimated worldwide cost of dementia is $818 billion, increasing to a trillion dollars by 2018 [Prince et al, 2015].

5 STUDY OBJECTIVES AND PURPOSE

5.1 Purpose of the Study

This phase 4, multicentre, open-label, randomised study will explore the impact of amyloid PET imaging on diagnostic thinking in the workup of patients with SCD-plus (subjective cognitive decline associated with features that increase the likelihood of preclinical Alzheimer's disease [AD]), mild cognitive impairment (MCI), or dementia where AD is in the differential diagnosis.

5.2 Research Hypothesis

The early adoption of Aβ PET imaging with [^{18}F]florbetaben or [^{18}F]flutemetamol will enable an earlier etiologic diagnosis with high confidence vs the late adoption of Aβ PET imaging with [^{18}F]florbetaben or [^{18}F]flutemetamol when used in patients with a cognitive impairment possibly due to AD, specifically in patients with SCD Plus, MCI, or dementia.

5.3 Rationale for Conducting the Study

Brain amyloidosis is one of the two key pathophysiological events of Alzheimer's disease. Amyloid PET imaging has demonstrated good analytical validity in pathologically confirmed studies, i.e. high sensitivity and specificity towards moderate or frequent amyloid plaques. However, despite effectively representing "in vivo neuropathology", its clinical utility is unclear.

AMYPAD will study in a real-life setting to what extent amyloid PET imaging affects diagnostic confidence, patient management, and ultimately the cost-effectiveness of dementia care. The dementia field has been moving from a syndromic diagnosis (e.g. "dementia") to an etiologic diagnosis (e.g. "dementia due to Alzheimer's pathology", "dementia due to Lewy body pathology", etc.). AMYPAD relies on the assumption that a more accurate etiologic diagnosis is associated with better patient care, treatment, and outcomes. The etiologic diagnosis of AD requires the detection of the two key pathophysiological phenomena of the disease, i.e. deposits of amyloid and hyper-phosphorylated tau proteins in the brain. The ascertainment of brain amyloid can rely on PET with appropriate ligands or CSF assays of Abeta42.

5.4 Objectives

The primary and secondary objectives of the study are as follows:

5.4.1 Primary objectives

To test the hypothesis that the proportion of participants for whom the managing physician reaches an etiologic diagnosis with very high confidence (≥90%) at 12 weeks after baseline is higher for participants who underwent amyloid PET imaging shortly after baseline than for participants who have not yet undergone amyloid PET imaging (ie, participants scheduled to undergo amyloid PET imaging at 8 months [±8 weeks] after baseline).

5.4.2 Secondary objectives

5.4.2.1 Diagnosis and confidence

To assess the impact of amyloid PET imaging on other diagnosis-related metrics:

- Time to communicate to the patient an etiologic diagnosis with very high confidence (≥90%)
- Changes in the managing physician's etiologic diagnosis over time.
- Changes in the managing physician's diagnostic confidence over time.
- The managing physician's estimate of the likelihood that the patient's symptoms are due to AD.
- How the placement of amyloid PET imaging in the clinical workup, when the managing physician is given free choice, changes over time.

5.4.2.2 Diagnostic/therapeutic management

To assess the impact of amyloid PET imaging on patient management, including:

- Number of patients randomised to disease-modifying drug (DMD) or any other AD clinical trial at 6 months from baseline
- Change or early adoption of programs and/or pharmacologic treatments aimed to delay the onset or progression of cognitive impairment
- Use of medical resources (including but not limited to diagnostic procedures, tests, programs, visits, and hospitalisations)

5.4.2.3 Health economics and patient-centred outcomes

To assess the impact of amyloid PET imaging on:

- Patient-related outcomes (cognition, anxiety, depression, coping skills, and quality of life)
- Cost of diagnostic workup to the etiologic diagnosis with very high confidence (≥90%)
- Number of patients who are discharged from the memory centre and the reason for discharge

5.4.2.4 Imaging results assessment

- To test the hypothesis that amyloid load is stable over 12 to 18 months.
- To develop standardised methods of image quantitation across the PET tracers in order to allow pooled analysis across the AMYPAD program.

6 STUDY DESIGN

6.1 Overall Study Design and Plan

6.1.1 Study design

This is a phase 4 multicentre, open-label study of amyloid PET imaging of participants who have one of the following syndromic diagnoses: SCD-plus, MCI, or dementia where AD is in the differential diagnosis. Participants will be randomised to 3 arms. Arms 1 and 2 allow testing the impact of amyloid PET on diagnostic confidence, patient management, and ultimately decision making and the cost-effectiveness of dementia care. Arm 3 allows to investigate the optimal timing of amyloid PET imaging in the diagnostic workup.

The baseline visit must take place within 14 days after the screening visit and can be combined with the screening visit. At baseline, the investigator will record the syndromic diagnosis made by the managing physician, the managing physician's confidence in that diagnosis, and the managing physician's estimate of the likelihood that the patient's symptoms are due to AD. (According to [Johnson et al, 2013], the managing physician is a dementia expert trained and board-certified in neurology, psychiatry, or geriatric medicine who devotes a substantial proportion [at least 25%] of patient contact time to the evaluation and care of adult acquired cognitive impairment or dementia.). The investigator and the managing physician may be but do not have to be the same person. Participants will be stratified by syndromic diagnosis (SCD-plus, MCI, or dementia) and then randomly assigned (1:1:1) to 1 of 3 arms:

- **Early Amyloid PET Arm:** Participants will undergo amyloid PET imaging within 4 weeks after the baseline visit. Amyloid PET will be carried out with high priority over other diagnostic exams except MRI in the early amyloid PET arm. The results of the scan must be communicated to the managing physician as soon as available. Early Amyloid PET arm participants will undergo an optional second amyloid PET scan within at 12 to 18 months window after the initial scan.
- **Late Amyloid PET Arm:** Participants will undergo amyloid PET imaging at 8 months (±8 weeks) after baseline.
  Free Choice Arm: The managing physician will decide whether the participant undergoes amyloid PET imaging. The imaging can be done at any time within 12 months after baseline. The reason for the choice will be collected.

In all Arms, between Baseline visit and Week 12 visit, each time a biomarker is available, the result must be communicated to the managing physician, and the etiological diagnosis as well as the diagnostic confidence must be collected immediately. This will provide a sequential collection of diagnosis and diagnostic confidence up to Week 12 visit.

Amyloid load will be defined on the basis of visual reads at all time points. Only for the secondary aim of the disease modelling, amyloid load will be defined quantitatively. The results of the optional second amyloid PET scans (early amyloid PET arm) will be provided to the managing physician and may be used for clinical purposes.

The goal will be to enrol 100 participants per stratum in each arm, for a total of 900 participants.

Sites will be using PET-CT or PET-MR depending on what technology is available at each site.

Participation in the study will not prevent participants from having any currently available diagnostic investigation, but rather will give participants the opportunity to have access to amyloid PET imaging, a diagnostic test approved by the European Medicines Agency but not yet reimbursed by most national health services.

The study will comprise the following clinical visits and timepoints:

- A baseline clinical visit (Visit 0 [V0] at Time 0 [T0]), within 14 days after screening
- A timepoint 12 weeks after baseline (T1); no clinic visit is required at that time
- A clinical visit at 6 months ±14 days (V1 or T2), and
- An optional clinical visit at 13 months ±4 weeks (V2 or T3)

Amyloid PET imaging (conducted at the local sites and read visually) will not be considered a separate clinical visit.

At T1, the investigator will review the result communicated by the managing physician (if the investigator and the managing physician are not the same person) and record the etiologic diagnosis made by the managing physician, rate the managing physician's diagnostic confidence (0% to 100%, visual analogue scale [VAS]) as well as the managing physician's estimate of the likelihood that the patient's symptoms are due to AD (0% to 100%, VAS), and record the effective date of the managing physician's etiologic diagnosis and management plan. This does not need to be a clinical visit.

At the baseline visit, all participants will receive a diary/questionnaire to collect information on the use of medical resources. Patients with dementia will be asked to provide a contact name for a caregiver who will be contacted in order to fill a part of the medical resources questionnaire. The caregiver session is requested only for patients with dementia. If there is no caregiver, that part of the questionnaire will be left blank.

The study design is outlined in Figure 1.

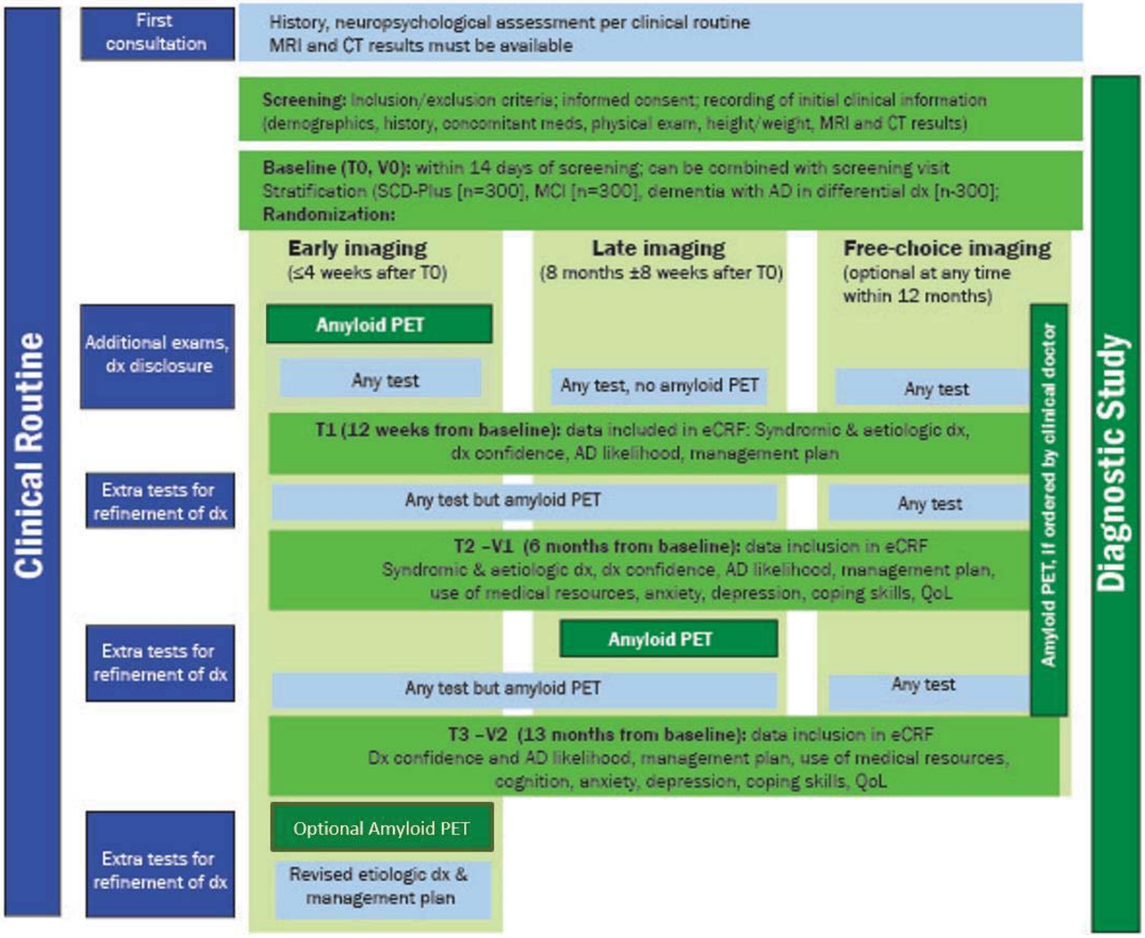

Figure 1 Study Diagram

6.1.2 Follow-up amyloid PET Image visit

Participants assigned to the Early Amyloid PET arm will have an optional second amyloid PET scan (performed 12 to 18 months after the initial scan) and an additional study visit (within 28 days after the optional second scan). The change in amyloid load between scans will be quantified.

AEs that occur within 48 hours of study drug administration and all SAEs occurring within 30 days of study drug administration will be recorded.

6.2 Study Rationale

The study will compare the impact of early vs late utilisation of amyloid PET imaging on the etiologic diagnosis, diagnostic confidence, and clinical management of possible cases of AD.

This study's safety monitoring plan is justifiable and adequate from a safety standpoint. In view of the positive safety profile of these imaging agents, including the minimal radiation applied by the tracer administration and transmission scanning, the foreseeable risks to the participants and negative impact on the participant's well-being will be minimal and, when considering other diagnostic procedures involving radiation, is considered low.

6.3 Study Timeframe

- **Expected start of the study (first participant's first visit):** the fourth quarter of 2017.
- **Expected end of study (last participant's last visit):** the fourth quarter of 2020.
- **Final clinical study report:** the third quarter of 2021.

6.4 Risks and Benefits to Participants

6.4.1 Potential risks

This Phase 4 study uses radiopharmaceutical products whose safety and efficacy were established in Phase 1 to Phase 3 studies.

The potential risk of amyloid PET imaging is exposure to radioactivity from the tracer ([^{18}F]florbetaben or [^{18}F]flutemetamol). However, the tracer doses planned in this study are very low, and only 300 out of 900 patients will receive a maximum of 2 doses within 12 to 18 months. Dosimetry data derived from animals and humans indicate that potential radiation risks associated with studies using [^{18}F]florbetaben or [^{18}F]flutemetamol are low and comparable to those of other radio tracers [NeuraCeq™ SmPC][Vizamyl SmPC]. Specifically for this study, a maximum radiation dose of 6.1mSv/year will be administered. This ionizing radiation exposure is acceptable for the included subjects. The reasoning behind this is that, according to ICRP guidelines, the relative detriment for adults over 50 years of age is one fifth or one tenth of the 6.1mSv, the latter being based on radiation exposure of the whole population. As such, the relative dose for detriment assessment (corrected to adult dose) for a subject is between 0.61 and 1.22mSv, which is category IIa to IIb risk level for the volunteer. According to ICRP guidelines this is justified if the level of societal benefit is intermediate to moderate, which is the case of this study.

The recommended activity for an adult is 300 MBq (±20%) for [^{18}F]florbetaben [NeuraCeq™ SmPC] and 185 MBq (+10%) for [^{18}F]flutemetamol [Vizamyl SmPC].

6.4.2 Potential benefits

We expect that amyloid PET imaging will help your clinician in having a higher confidence in his/her diagnosis regarding your condition, and in his/her prognosis and planning for your care management
The potential benefit to participants and treating physicians is knowledge about the participant's Aβ status in the setting of cognitive impairment when the participant is undergoing evaluation for cognitive impairment possibly due to AD (SCD Plus, MCI or dementia).

- Demonstrating the absence of Aβ pathology in participants with signs of cognitive impairment or dementia may facilitate a more accurate diagnosis and management, and a search for other, potentially reversible underlying aetiologies.
- On the other hand, knowledge of the presence of Aβ may allow specific counselling on treatment interventions, prognosis, and planning.

It will be clarified that participation in the study will not prevent any currently available diagnostic option, but rather will give the opportunity to have access to the amyloid PET imaging, a diagnostic test approved by the European Medicine Agency but not yet reimbursed by most national health services.

6.4.3 Risk/benefit analysis

Previous studies consistently reported that amyloid PET imaging facilitates early identification of AD pathology and significantly enhances the specificity of diagnosis [Nordberg 2010]. The apparent minimal risk of the use of these radiolabelled imaging agents must be weighed against the benefit that Aβ status has a significant impact on patient outcome and prognosis and allows targeted treatment intervention including a clinical management plan to be devised for an individual participant with better outcomes. The risks will be continuously monitored, assessed, and documented by the investigator.

Regarding potential benefits, individual patients can benefit from the study from a more accurate etiological diagnosis. In addition, participants can benefit whenever the diagnosis confidence after the PET scan increases, or when the etiological diagnosis is reached and/or management changes due to the results of the PET scan can be implemented. Moreover, future patients may benefit from this study as the appropriate use of amyloid PET in clinical routine to reach an etiological diagnosis might be expanded after this research is final.

If the risks will be found to outweigh the potential benefits or if there is conclusive proof of definitive outcomes, investigators will assess whether to continue, modify or immediately stop the study.

This information on risk and benefit will be conveyed to all participants (and their representative, if appropriate) verbally and in writing as part of the informed consent process prior to enrolment in the trial. In view of robust safety, tolerability, and minimal participant exposure of the drug substance and the clear medical need for tracers of this type, the low radiation risk appears justified.

7 SELECTION AND WITHDRAWAL OF PARTICIPANTS

7.1 Procedures for Enrolment

All consecutive patients coming to a participating Memory Clinic and their satellite centres with a request for diagnosis and appropriate age will be considered for the study. Some screening activities will be part of the clinical routine while others will be part of the clinical study. Written Informed Consent must be obtained before any study-specific screening procedures are performed.

7.2 Eligibility

Participants who fulfil all respective selection criteria and meet none of the exclusion criteria and satisfy the criteria for SCD Plus, MCI, or dementia where AD is in the differential diagnosis will be eligible for enrolment into the study. Written informed consent, which will be dated with time of consent noted, will be obtained from all participants. If necessary (i.e. in case of patient with dementia), written informed consent will be also obtained by the legal representative, and by a study partner, prior to study entry and prior to any protocol-specific procedures.

7.3 Inclusion Criteria

To be enrolled in the study, participants must meet all of the following criteria:

- The patient can be of any sex, gender, race, or ethnicity.
- SCD-Plus patients must be 60 to 85 years of age.
- Patients with MCI or with dementia must be 50 to 85 years of age.
- The patient must have a complaint (reported by the patient or by a caregiver) of cognitive problems that are considered by the managing physician to be possibly due to AD.
    - The patient must be entering a diagnostic assessment for the cognitive complaint.
    - The managing physician must feel that knowledge of the patient's brain amyloid status may increase diagnostic confidence and alter diagnosis and management.
    - Patients should not have known amyloid status prior to randomization.

- The patient must satisfy the diagnostic criteria for one of the following:
    - SCD-Plus (see Section 7.5.1)
    - MCI (see Section 7.5.2)
    - Dementia, where AD is in the differential diagnosis (see Section 7.5.3)
- The patient has undergone a dementia blood workup or will have one before amyloid PET.
- The patient has an MRI and/or CT scan (not older than 12 months) or will undergo one before amyloid PET.
- The patient can complete all clinical visits according to the protocol.
- The patient can tolerate a 20-minute amyloid PET scan.
- The patient (or, in case the patient is incapable of giving informed consent, next of kin or a legal representative as per national requirements) provides informed consent for study participation and data source verification. In case of a change in the capacity to consent, the local regulations will be applied. In case the patient is randomized to the Early Amyloid

PET arm, a new informed consent should be signed before the optional second imaging session.
- If the patient has dementia, a study partner is available for the duration of the protocol.
- In countries where patients unable of giving informed consent cannot be included in the study and an assessment of the patients’ capacity to consent is requested, a study physician will assess the capacity to consent in patients with verifiable cognitive impairment prior to enrolment and regularly in the course of the study. If there are any doubts as to the patients' capacity to consent, the capacity to consent will be conclusively assessed by a specialist. Patients who are unable of giving informed consent, are excluded from the study in these countries.

7.4 Exclusion Criteria

Patients must be excluded from participating in this study if they meet any of the following criteria:

- The patient has another confirmed condition that can fully account for the cognitive impairment, including but not limited to psychiatric disorders (schizophrenia, mood disorders, bipolar disorder and personality disorders); neuroinflammatory, neuroinfective, or neurodegenerative diseases; multiple sclerosis; genetic disorders; HIV; brain injuries; neurosurgery aftereffects, delirium). Patients with long-known, stabilized psychiatric or other brain conditions that cannot fully account for the cognitive impairment may be included in the study.
- The patient comes to observation for reasons other than diagnosis (disability assessment for social aids, cognitive assessment for driving license, etc.)
- The patient had a previous Aβ imaging scan and/or has had other AD biomarker workup (fluorodeoxyglucose [FDG]-PET and/or cerebrospinal fluid [CSF] analysis) prior to screening. In some centres, the patient may receive a diagnostic workup before screening. These patients can be enrolled if the investigator is blind to the results until after randomization.
- The patient has a life-threatening unstable medical disease or psychiatric condition that could lead to difficulty in complying with the protocol.
- The patient is currently receiving an investigational pharmaceutical product or has participated in a clinical trial with an investigational pharmaceutical product within 30 days prior to screening, and/or was administered a radiopharmaceutical within 10 radioactive half-lives prior to study drug administration in this study.
- The patient is a woman who is pregnant, planning to become pregnant, or lactating. Pregnancy status of a woman with childbearing potential will be carried out before the PET scan. A woman is considered of childbearing potential (WOCBP), i.e. fertile, following menarche and until becoming post-menopausal unless permanently sterile (www.hma.eu/ctfg.html, Recommendations related to contraception and pregnancy testing in clinical trials, September 2014).
- The patient is not affiliated to the Social security and does not receive the rights and corresponding coverage, in accordance with the Code of Public Health.

- The patient is employed at the research department or memory clinic, it is directly involved with the study, and it is a family relative from any department personnel (i.e. partner, older child, sibling, biological or legal representative).
- Any of the contraindications as registered for the study drug used is applicable to the subject. Any of the warnings or precautions as registered for the IMP used is applicable to the subject, unless a risk-benefit assessment is favorable as per the judgement of the sponsor.

7.5 Diagnostic Criteria

During screening, the following criteria will be used for giving patients a syndromic diagnosis of SCD Plus, MCI, and dementia where AD is in the differential diagnosis.

7.5.1 Subjective cognitive decline plus (SCD-Plus), modified from [Jessen et al, 2014]

Subjective cognitive decline (SCD) in individuals with unimpaired performance on cognitive test may represent the first symptomatic manifestation of Alzheimer's Disease (AD). The following list of features refers to SCD-Plus, in which the likelihood of the presence of preclinical AD is increased:

Inclusion criteria for SCD-Plus:

- The patient is ≥60 years old.
- The patient has perceived a decline in memory over time.
- The onset of the cognitive decline is within the previous 5 years and the duration is >6 months
- The Mini-Mental State Examination® (MMSE®) score is 27 to 30 out of 30.
- The clinical examination and neuropsychological assessment exclude MCI.
- Cognitive decline has been confirmed by an informant.
- The patient (or caregiver) has expressed concerns (worries) about the cognitive symptoms.

Exclusion criteria for SCD-Plus:

- Current or past psychiatric disorders according to ICD 10 (including major depression, anxiety disorder, substance-related disorders, schizophrenia, bipolar disorder, adult ADHD, post-traumatic stress disorder). However, a depressive episode, an anxiety disorder, or a substance-related disorder that occurred >5 years earlier and in no temporal association with the onset of SCD is not a criterion for exclusion.
- Current or past history of a neurologic disease with known potential impact on cognition.
- MRI lesions that would not be consistent with a diagnosis of AD.
- Current use of medication with known effect on cognition, including sedatives and drugs with anticholinergic effect, if the clinician believes that the use of those drugs is the cause of cognitive impairment.

7.5.2 Mild cognitive impairment (MCI) [Albert et al, 2011]

Diagnostic criteria for MCI are based on the National Institute on Aging and Alzheimer's Association (NIA-AA) Core Clinical Criteria:

- Concern regarding a change in cognition, as expressed by the patient, a proxy, or a physician.
- Impairment in one or more cognitive domains, as defined by neuropsychological test scores ≥1.5 SD below the age- and education-specific mean.
- Preservation of independence in functional abilities.
- No dementia.

7.5.3 Dementia where AD is in the differential diagnosis:

7.5.3.1 Probable AD dementia

The diagnostic criteria for probable AD dementia are based on the NIA-AA core clinical criteria in addition to the criteria for dementia [McKhann et al, 2011]

- Insidious onset
- Clear-cut history of worsening of cognition by report or observation
- The initial and most prominent cognitive deficits are evident on history and examination
- Presentation can be amnestic presentation or nonamnestic (language, visuospatial, executive function, etc)
- The diagnosis of probable AD dementia should not apply when there is evidence of substantial concomitant cerebrovascular disease, dementia with Lewy bodies, behavioural variant of frontotemporal dementia, semantic variant primary progressive aphasia or non-fluent/agrammatic variant primary progressive aphasia, evidence for another concurrent, active neurological disease, or non-neurological medical comorbidity or use of medication that could have a substantial effect on cognition.

7.5.3.2 Possible AD dementia

The diagnostic criteria for possible AD dementia are based on the NIA-AA core clinical criteria in addition to the criteria for dementia [McKhann et al, 2011]:

- Atypical course (either a sudden onset of cognitive impairment or insufficient historical details or objective cognitive documentation of progressive decline.)

7.6 Withdrawal and Termination Criteria

7.6.1 Participant withdrawal

There are no formal withdrawal criteria for this study. During the conduct of the study, the Sponsor will review the safety data for trends and signals that would indicate the need for withdrawal of a participant.

In accordance with the Declaration of Helsinki, each participant is free to withdraw from the study at any time. Investigator(s) also have the right to withdraw participants from the study in the event of illness, adverse events (AEs), or other reasons concerning the health or well-being of the participant, or in the case of lack of co-operation.

Should a participant decide to withdraw after administration of the IMP(s), or should the investigator(s) decide to withdraw the participant, all efforts will be made to complete and report the observations up to the time of withdrawal as thoroughly as possible. A complete final evaluation at the time of the participant's withdrawal should be made and an explanation given of why the participant is withdrawing or being withdrawn from the study. The reason for withdrawal, if the participant will give one, must be noted in the electronic Case Report Form (eCRF). If the reason for withdrawal is a clinical AE, monitoring will continue until the outcome is evident. The specific event or test result(s) must be recorded in the eCRF.

7.6.2 Replacement of participants withdrawn from the study

Participants who are withdrawn will not be replaced.

7.6.3 Premature termination of study or site

There are no formal withdrawal or termination criteria for this study.

7.6.3.1 Study termination

7.6.3.2 In case of premature termination or suspension of the study, the Sponsor will promptly inform the investigator, regulatory authorities, and independent ethics committees (IECs)/institutional review boards (IRBs) of the termination or suspension and the reason that this was deemed necessary. Site termination

The study may be terminated at an individual centre if:

- The centre cannot comply with the requirements of the protocol.
- It is not possible for the centre to comply with Good Clinical Practice (GCP) standards.

8 TREATMENT OF PARTICIPANTS

8.1 Investigational Medicinal Products

The investigational products will be supplied by Piramal and GE Healthcare. Provision of Florbetaben and Flutemetamol will be under the responsibility of the drugs' contract manufacturers (on behalf of Piramal and GEHC), which will supply free doses for the study, as stated in the AMYPAD Grant Agreement (Description of Action).

8.1.1 NeuraCeq™ ([^{18}F]florbetaben)

INN: Florbetaben (^{18}F)

Chemical name: [^{18}F] Florbetaben [trans-4-(N-methyl-amino)-4′-{2-[2-(2-[^{18}F]-fluoro-ethoxy)-ethoxy]-ethoxy}-stilbene]

Chemical formula: $C_{21}H_{26}{}^{18}FNO_3$

Molecular weight: 358.45 [g/mole]

Physical, chemical, and pharmaceutical properties:

Appearance: clear solution

NeuraCeq™ is a radiopharmaceutical indicated for positron emission tomography (PET) imaging of Aβ neuritic plaque density in the brains of adult patients with cognitive impairment who are being evaluated for Alzheimer's disease (AD) and other causes of cognitive impairment.

The active pharmaceutical ingredient florbetaben for PET imaging is manufactured under current Good Manufacturing Practice (GMP) and labelled with the radioactive fluoride isotope ^{18}F, which has an average half-life of 110 minutes. The radioactive drug substance and the formulated final drug product injectable doses are produced by Piramal Imaging approved contract manufacturer near the clinical study site according to the radiolabelling and purification procedure [NeuraCeq™ SmPC].

Each batch of Florbetaben (^{18}F) produced must meet criteria listed in the specification for identity, purity, concentration, specific activity, and pH before being released. Manufacturing and quality control testing will be checked by the responsible quality person of the manufacturer and will be documented accordingly. Sterility tests will be conducted as control of the validated production process after release of the product according to the established procedures.

The final product will be formulated as a sterile solution for IV injection. The radioactivity of the final product will be verified before injection by using a suitable counter, as established on site.

NeuroCeq™ Florbetaben (^{18}F) Injection will be provided by the manufacturer in multidose vial, containing the specified radioactive dose and total quantity of the investigational product. The syringe will be shielded by secondary lead containers for radiation protection purposes.

Further details can be found in the approved Summary of Product Characteristics, which contains comprehensive information on the study drug [NeuraCeq™ SmPC].

8.1.2 Vizamyl™ ([^{18}F]flutemetamol)

INN: Flutemetamol (^{18}F)

Chemical name: 2-[3-[^{18}F]fluoro-4-(methylamino) phenyl]-6-benzothiazolol

Chemical formula: $C_{14}H_{11}{}^{18}FN_2OS$

Molecular weight: 273.32 [g/mole]

Physical, chemical, and pharmaceutical properties

Appearance: clear, colourless to slightly yellow, solution

pH: between 6.0 and 8.5

Vizamyl™ is a radiopharmaceutical indicated for PET imaging of Aβ neuritic plaque density in the brains of adult patients with cognitive impairment who are being evaluated for AD and other causes of cognitive impairment.

The active pharmaceutical ingredient flutemetamol for PET imaging is manufactured under current GMP and labelled with the radioactive fluoride isotope fluorine-18, which has an average half-life of 110 minutes. The radioactive drug substance and the formulated final drug product injectable doses are produced by GE Healthcare-approved manufacturing site based near the clinical study site according to the radiolabelling and purification procedure [Vizamyl SmPC].

Each batch of Vizamyl or Flutemetamol (^{18}F) Injection produced must meet criteria listed in the specification for identity, purity, concentration, specific activity, and pH before being released. Manufacturing and quality control testing will be checked by the responsible quality person of the manufacturer and will be documented accordingly. Sterility tests will be conducted as control of the validated production process after release of the product according to the established procedures.

The final product will be formulated as a sterile solution for IV injection. Flutemetamol (^{18}F) Injection should be stored at 2 to 30 °C in a shielded container. However, temperature excursions in the range up to 50 °C during transport are acceptable. The radioactivity of the final product will be verified before injection using a suitable counter, as established on site.

Further details can be found in the approved Summary of Product Characteristics and Investigators Brochure, which contain comprehensive information on Vizamyl and Flutemetamol (^{18}F) Injection, respectively [Vizamyl SmPC].

Flutemetamol (^{18}F) Injection is supplied with a radioactive content of 150 MBq/mL at the reference date and time as specified on the label. However, Vizamyl is supplied with a radioactive content of 400 MBq/mL at the reference date and time. This difference is because the manufacture of Vizamyl, as required per the marketing authorisation from the European Medicines Agency (EMA), involves an additional step of dilution at end of synthesis to give a fixed strength product. The recommended dose for an adult is 185 MBq (± 10%), regardless of the formulation used.

8.1.3 Packaging and labelling

Florbetaben (^{18}F) will be provided by the manufacturer in multi-dose vials, containing the specified radioactive dose(s) and total quantity of the investigational product. The vial will be shielded by secondary lead containers for radiation protection purposes.

Flutemetamol (^{18}F) Injection will be provided by the manufacturer in a ready-to-use injection syringe or in a glass vial, containing the specified radioactive dose and total quantity of the investigational product. The vial or syringe will be shielded by secondary lead containers for radiation protection purposes.

For all study products, a system of medication numbering (batch numbers) in accordance with all requirements of GMP will be used. This will ensure that for each participant, any dose of study drug can be identified and traced back to the production run.

8.1.4 Drug supplies, logistics, and accountability

8.1.4.1 Study supplies

The nuclear medicine physician or designee is responsible for ensuring that deliveries of study drug from the manufacturing sites are correctly received, recorded, handled, and destroyed safely and properly in accordance with all applicable regulatory guidelines, and used in accordance with this protocol.

Any residual content of the vial/syringe will be disposed of at the study site by the end of the PET scan. Receipt, distribution, and disposal of the study drug must be properly documented. Study drug vial/syringe must be destroyed on-site after use per local standard operating procedures. The manufacturing site will arrange collection of the transport containers.

The current approved product label will be supplied to the investigative centres using commercial product, and the Investigator brochure will be supplied to the centres using Flutemetamol (^{18}F) Injection.

8.1.5 Drug logistics

NeuraCeq™ and Vizamyl™ will be manufactured and handled according to the applicable GMP at a qualified manufacturing sites as approved. All manufacturing, quality control testing,

and documentation will be controlled and documented by the responsible person at the production site.

A complete record of batch numbers and expiry dates of the radiopharmaceutical products used in this study will be maintained in the Trial Master File (TMF). The production sites will allocate individual batch numbers when producing study drug. A complete batch documentation in which manufacturing, quality control, analytical results, and the batch release for human use are detailed will be generated according to locally established standards. These documents will be archived at the respective production site.

Appropriate documentation (for order/receipt, dosage instructions and release) will be provided with each delivery of study drug which will contain the batch number, time of preparation, and radioactive concentration of injection (MBq/mL) at the time of calibration. The final quality control processes for the study drug will occur and be documented before the substance is administered to the participant at the imaging clinic. The qualified person at the manufacturing site will ensure that the product quality meets the defined criteria and will declare this in a document for release, to be received by the investigator (or designated personnel) at the imaging clinic. The study drug will not be administered to the participant without release for human use.

8.2 Selection of Doses and Timing

The recommended doses are those recommended in the approved product Summary of Product Characteristics [NeuraCeq™ SmPC][Vizamyl SmPC].

Doses outside of the approved range will be considered as protocol deviations (see Section 13.5).

8.2.1 Drug accountability

The nuclear medicine physician (or designated personnel) will confirm receipt of the study drug in writing and will use the study drug only within the framework of this clinical study and in accordance with this study protocol. For each participant, the nuclear medicine physician or designee will keep a record of the study drug dispensed. These documents are to be filed in the investigator site file.

Receipt, distribution, and disposal (if applicable) of the study drug must be properly documented on the forms provided by the ligand manufacturer, giving the following information: study protocol number, sender, receiver, date, quantity, batch number, expiration date, and retest date (if applicable).

8.2.2 Dosage and administration of study drug

Study participants will be administered NeuraCeq™ or Vizamyl™ under the direct supervision of a Nuclear Medicine physician or designee. For the dose and administration see the respective summary of product characteristics (SmPC) for NeuraCeq™ [NeuraCeq™ SmPC] and Vizamyl™ [Vizamyl SmPC] and the Investigator Brochure for flutemetamol [[18F Flutemetamol].

8.2.3 Measurement and documentation of the injected radioactivity:

The precise administered fluorine-18 net radioactivity must be calculated from the difference of radioactivity measured in the syringe/injection system prior to and after injection. For radioactivity measurement, a suitable counter system must be used.

The following data will be recorded in the CRF:

- Batch number
- Date and time of injection
- Duration of injection (seconds)
- Volume of injection (mL)
- Radioactivity in syringe prior to injection (mCi, measured) and time of measurement
- Radioactivity in syringe and injection system after injection (mCi, measured) and time of measurement
- Radioactivity injected (mCi), calculated as difference of the radioactivity of the syringe measured prior to and the syringe and injection system measured after the injection.

8.2.4 Investigational Medicinal Product (IMP) accountability

For all study products, a system of medication numbering (batch numbers) in accordance with all requirements of GMP will be used. This will ensure that for each participant, any dose of study drug can be identified and traced back to the production run.

The Nuclear Medicine Physicians (or designated personnel) will document receipt of the study drug and will use the study drug only within the framework of this clinical study and in accordance with this study protocol. For each participant, he/she will keep a record of the study drug dispensed. These documents are to be filed in the investigator site file.

The Nuclear Medicine physicians (or designated personnel) are responsible for ensuring that deliveries of IMP(s) are correctly received, recorded, handled, and stored safely and properly in accordance with all applicable regulatory guidelines, and used in accordance with this protocol.

8.2.5 Registration of investigational medicinal product(s) complaints

In the event of an IMP complaint (e.g., breakage, leakage, particulate matter, discoloration), the nuclear medicine physician or recipient of the IMP is requested to report the problem on the IMP shipping documentation (e.g., ‘Delivery Note for Product,’ Drug Shipping and Receiving Form, or equivalent form). This should be promptly forwarded to the person indicated on the shipping documentation. Once received, the Clinical Supplies Manager will register the complaint and determine if the complaint is minor or significant according to Sponsor procedures. All complaints will be followed-up and the appropriate action will be implemented according to Piramal and GE procedures.

8.3 Method of Numbering Participants and Assigning Participants to Treatment Groups

8.3.1 Participant identification numbers (PIDs)

At the screening visit, upon signing of the informed consent form, each participant will be assigned a unique 6-digit participant identification number (PID) by the site for unambiguous identification. Once allocated, the participant's PID will identify the subject throughout the study, and will be entered into the Site Enrolment Log and on the eCRF. The PID will be constructed as follows:

First 2 digits: centre code (e.g., 01, 02….)

Next 4 digits: unique participant code (e.g., 0001, 0002, 0003….)

For example, the first participant at the third site will have PID of 030001 and the second participant will be 030002.

8.3.2 Randomisation

This is a phase 4, open label, randomised study.

Patients will be randomised in a 1:1:1 ratio into 1 of the following 3 groups:

- **Early Amyloid PET arm:** These participants will undergo amyloid PET imaging within 4 weeks after baseline. The results of the PET imaging must be communicated to the managing physician as soon as they are available. Amyloid PET will be carried out with high priority for early amyloid PET arm patients. For participants in this arm, an optional second amyloid PET scan will be performed 12 to18 months (±28 days) after the initial scan.
- **Late Amyloid PET arm:** These patients will undergo amyloid PET imaging at 8 months ±8 weeks after baseline.
- **Free Choice arm**: The managing physician will choose whether and when each participant will have amyloid PET imaging at any time between baseline and 12 months after baseline.

Randomization will be performed by using permuted blocks assuring equal numbers to all arms. Randomization will be stratified by centre and baseline syndromic diagnosis. Participants will be randomly assigned (1:1:1) to 1 of 3 arms and randomization will continue until 300 SCD plus, 300 MCI, and 300 dementia where AD is a differential diagnosis have been included.

8.4 Selection of Doses and Timing

In each imaging session, a single intravenous (IV) dose of amyloid PET tracer ([^{18}F]florbetaben or [^{18}F]flutemetamol) will be used:

- For [^{18}F]florbetaben, the recommended activity is 300 MBq (±20%).
- For [^{18}F]flutemetamol, the recommended activity is 185 MBq (±10%).

The recommended doses are those in the approved product Summary of Product Characteristics [NeuraCeq™ SmPC][Vizamyl SmPC].

Doses outside of the approved range will be considered as protocol deviations (see Section 13.5).

8.5 Blinding

This is an open-label study. Thus, there will be no blinding with regard to treatment assignments.

8.6 Prior and Concurrent Therapy

Prior medication refers to medication taken within 4 weeks before injection of the study drug. Concomitant medication refers to medication received by the participant from the time of injection of the study drug.

In this study, there are no restrictions on prior and concomitant medications.

Any medications taken by the participant within 4 weeks before and up to the end of the observation period will be recorded in the eCRF along with the indication and dosage. Either the generic or the trade name may be recorded. The Sponsor/CRO will encode all therapy and medication according to a current well-recognised dictionary of medical codes.

8.7 Food Restrictions

There are no food or drink restrictions in this study.

8.8 Treatment of Study Drug Overdose

The investigational product will be administered as a single IV injection under the direct supervision of a Nuclear Medicine physician or designee, which reduces the likelihood of an overdose occurring.

8.9 Treatment Compliance

The study treatments will be administered as a single IV injection under the direct supervision of a nuclear medicine physician or designee. Thus, compliance is expected to be 100%. However, any departures from the intended regimen will be recorded.

Each administration volume and total radioactivity injected will be checked and the vial code and volume per administration will be recorded in each participant's eCRF. Doses administered outside of specific dose requirements or defined range must be reported as protocol deviations (see Section 13.5).

9 STUDY PROCEDURES

All efficacy and safety measurements obtained during the course of the main study are summarised in the Study Schedule of Events (Table 1).

Table 1. Study Schedule of Events

**Event/Visit**		**Baseline (within 14 d after screening)**	**≤4 w after T0**	**12 w after T0**	**6 m (±14 d) after T0**	**8 m (±8 w) after T0**	**13 m (±14 d) after T0 - optional**	**12 to 18 m after 1st scan[a]**	**≤28 d after 2nd scan[a]**
**Visit**	**Screening**	**V0**			**V1**		**V2**		**V3**
**Time points**		**T0**		**T1**	**T2**		**T3**		**T4**
Visit type	Clinic	Clinic			Clinic		Clinic		
Explain study to participants	X								
Obtain informed consent	X								
Demographics	X								
History (family & medical)	X								
Review of current medications	X		X [b]		X [b]	X [b]	X	X [b]	
Physical examination	X								
Review inclusion/exclusion criteria including pregnancy test if applicable	X								
Vital signs	X								
Height/weight	X								
Cognition/functioning (episodic memory, verbal memory, attention, verbal fluency, visual/spatial function, executive function)	X				X		X		
Dementia blood workup	X								
MRI [c]	X								
CT [c]	X								
MMSE®		X			X		X		
EQ5D-5L		X			X		X		
ICECAP-O		X			X		X		
Brief COPE		X			X		X		
Hospital Anxiety & Depression Scale		X			X		X		
Use of medical resources as collected in the patient diary/questionnaire				X	X		X		
Managing physician's syndromic diagnosis	X	X		X	X		X		

Managing physician's etiologic diagnosis		X		X	X		X		X
Managing physician's management plan				X	X		X		X
Managing physician's diagnostic confidence		X		X	X		X		
Randomisation [d]		X							
Amyloid PET imaging [e]			X [f]			X [g]		X	
Managing physician's estimate of the likelihood that the symptoms are due to AD	X	X		X	X		X		
Adverse events [h]			X	X	X	X	X		X
Amyloid PET scan results [i]		X [i]		X [i]	X [i]	X [i]	X [i]		X
FDG-PET		(X)		(X)	(X)		(X)		
SPECT		(X)		(X)	(X)		(X)		
Lumbar puncture		(X)		(X)	(X)		(X)		
Optional blood tests (eg, genetic testing)		(X)		(X)	(X)		(X)		
Optional neuropsychologic testing		(X)		(X)	(X)		(X)		
Other		(X)		(X)	(X)		(X)		

Brief COPE = Brief Coping Orientation to the Problems Experienced; CT = computed tomography; EQ5D-5L= EuroQoL 5-dimension questionnaire; MRI = magnetic resonance imaging;
Items in *italics* denote the clinical routine activities. (X) indicates diagnostic tests that are not mandatory and may be ordered at the managing physician's discretion; the results will be reviewed with the managing physician and patient at any following visit.

a Only the participants who are randomized to the Early Amyloid PET arm will undergo an optional second amyloid PET imaging scan 12 to 18 months after the initial scan and attend the T4 visit.
b Concomitant medications are recorded on the day of amyloid PET imaging.
c In order to be enrolled in the study, the participant must have had an imaging study (MRI or CT), not older than 12 months, to exclude intracranial mass or other lesions that might explain the cognitive impairment. Results must be available before enrolment.
d Randomisation will occur after all screening assessments are completed
e For the Early Amyloid PET arm, amyloid PET imaging will be done ≤4 weeks after T0 and again 12 to 18 months after the first imaging session. For the Late Amyloid PET arm, amyloid PET imaging will be done at 8 months (±8 weeks) after T0. If the managing physician chooses to order amyloid PET imaging for a participant in the Free-Choice Imaging arm, the imaging can take place at any time from T0 to 12 months after T0.
f Early Imaging arm,
g Late Imaging arm.
h Treatment-emergent adverse events that occur within 48 hours of study drug administration (amyloid PET imaging) will be collected. Serious adverse events that occur within 30 days of study drug administration will be collected.
i Amyloid PET imaging will be performed with high priority within 4 weeks after the baseline visitfor participants in the Early Amyloid PET arm and at 8 months ± 8 weeks for participants in the Late Amyloid PET arm. Participants in the Early Amyloid PET arm will receive an optional second amyloid PET scan 12 to 18 months after the first scan. If amyloid PET imaging is done for a participant in the Free Choice arm, the amyloid PET imaging can be done at any time between baseline and 12 months. The results of the amyloid PET scan will be recorded in the eCRF as soon as they are available. The results of the amyloid PET scan will be reviewed with the managing physician and patient and communicated to the investigator (if the managing physician and the investigator are not the same person).

9.1 Screening Period

All consecutive patients who are of the appropriate age and who come to a participating memory clinic will be considered for the study. Some of the screening activities will be part of the clinical routine; others will be specific to this study.

During the Screening period, the investigator (or the managing physician, if the investigator and the managing physician are the same person) will provide a written description of the study and explain the study to the potential participants and their caregivers. The patient (and study partner, and/or the legal representative if applicable) will be informed that participation in the study will not prevent any currently available diagnostic option. Rather, it will give the patient the opportunity to have access to amyloid PET imaging, a diagnostic test approved by the European Medicines Agency but not yet reimbursed by most national health services.

Patients will be also informed that because of the randomisation:

- Participants assigned to the Early Amyloid PET arm will undergo amyloid PET imaging within 4 weeks after baseline and again 12 to 18 months (±28 days) after the first scan.
- Participants assigned to the Late Amyloid PET arm will undergo amyloid PET imaging at 8 months (±8 weeks) after baseline.
- For participants assigned to the Free Choice arm, the managing physician decides whether and when to order amyloid PET imaging; this imaging can be done at any time within 12 months after baseline.

During the screening visit, the investigator must ensure that all participants satisfy all the inclusion criteria listed in Section 7.3 and none of the exclusion criteria listed in Section 7.4. Waivers or protocol exceptions will not be granted prospectively by the Sponsor under any circumstances. Any exceptions to protocol-specified requirements will be considered as protocol deviations.

Signed and dated informed consent must be obtained from all participants prior to their entering the study. After the Informed Consent form is signed, the investigator will record in the participant's eCRF the results of the exams and the assessments performed by the managing physician in clinical routine since the request for diagnosis. (The investigator and the managing physician may be, but do not have to be, the same person.)

The following information will be recorded for all participants during the screening period:

- Demographics
- Personal and family medical history
- Review of current medications
- Physical examination
- Vital signs
- Height and weight
- Dementia blood workup, consisting in:
    - – Complete blood cell count

  - Serum electrolytes
  - Glucose
  - BUN/creatinine
  - Serum B12 levels
  - Thyroid function tests
  - Liver function tests
- Evaluation of cognition/functioning (episodic memory, verbal memory, attention, verbal fluency, visual/spatial function, executive function)
- Results of brain magnetic resonance imaging (MRI) or computed tomography (CT) not older than 12 months before screening, showing the absence of an intracranial mass or other lesion(s) that might explain the cognitive impairment/complaint. Participants are eligible for the study only if these results are available.
- Managing physician's syndromic diagnosis
- The managing physician's estimate of the likelihood that the patient's symptoms are due to AD

9.2 Baseline Visit (V0 at T0)

The baseline visit must occur within 14 days after the screening visit. The screening visit can be combined with the baseline visit.

At the Baseline Visit (Visit 0 [V0] at Time 0 [T0]), the participants/study partners will receive a diary/questionnaire. At the Baseline visit, the results of the following will be recorded for all participants:

- Mini-Mental State Examination (MMSE®)
- EQ5D-5L (EuroQoL 5-dimension questionnaire)
- ICECAP-O (ICEpop CAPability measure for Older people)
- Brief COPE – Brief Coping Orientation to the Problems Experienced)
- Hospital Anxiety and Depression Scale (HADS)
- Managing physician's syndromic diagnosis (SCD-Plus, MCI, or dementia where Alzheimer's Disease is in the differential diagnosis)
- Maximum 4 hypotheses of etiologic diagnosis made by the managing physician, each with a diagnostic confidence (only if the diagnostic confidence >90% is not achieved for one etiologic diagnosis hypothesis)
- Diagnostic confidence (the managing physician's confidence that the diagnosis is correct) for each hypothesis.
- The managing physician's estimate of the likelihood that the patient's symptoms are due to AD.

Results of an already available neuropsychological assessment can be used at Baseline visit only if the results are not older than 6 months and based on the managing physician clinical judgement that there are no reasons to expect a change in that period.

The participants will be stratified according to the syndromic diagnosis made by the managing physician and then randomised, as described in Section 8.3.2.

For participants in the Early Amyloid PET arm, amyloid PET imaging will be scheduled to take place within 4 weeks after baseline. Amyloid PET will be carried out with high priority for early amyloid PET arm patients.

For participants in the Late Amyloid PET and Free Choice arm, if results of any of the following tests are available at this visit, the results will be reviewed with the participant/study partner and recorded in the eCRF:

- FDG-PET
- Lumbar puncture
- Optional blood tests
- Single photon emission computed tomography (SPECT) (specify test/s)
- Optional neuropsychiatric testing
- Other tests

9.3 Amyloid PET Imaging for Participants in Early Amyloid PET Arm (≤4 Weeks After Baseline)

All female participants of childbearing potential must have a negative pregnancy test before the imaging study is done.

The participants in the Early Amyloid PET arm of the study will undergo amyloid PET imaging within 4 weeks after baseline.

If the managing physician chooses to order amyloid PET imaging for the participants in the Free Choice arm, those participants may undergo the imaging at any time from baseline to 12 months after baseline.

The following will be recorded for all participants who have undergone amyloid PET imaging:

- Amyloid PET scan results
- All AEs that occur within 48 hours of study drug administration
- All SAEs occurring within 30 days of study drug administration
- Review of concurrent medications

Amyloid PET will be carried out with high priority for early amyloid PET arm patients.

.

9.4 T1, 12 Weeks After Baseline

At T1, which is defined as 12 weeks after baseline, the following will be recorded for all participants (a clinic visit is not strictly necessary at this timepoint):

- Use of medical resources, as collected in the patient questionnaire
- Syndromic diagnosis made by the managing physician

- Between Baseline and Week 12, each time a biomarker is available, the result must be communicated to the managing physician, and the etiological diagnosis as well as the diagnostic confidence must be collected immediately. This will provide a sequential collection of diagnosis and diagnostic confidence up to Week 12 visit, and all will be recorded at the Week 12 eCRF.
- Management plan made by the managing physician.
- The managing physician's estimate of the likelihood that the patient's symptoms are due to AD.

Note that amyloid PET imaging was to be performed within 4 weeks of baseline for the members of the Early Amyloid PET arm. If the managing physician chooses to order amyloid PET imaging for a member of the Free Choice arm, the imaging can be performed at any time from baseline to 12 months after baseline. At T1, the following will be recorded:

- Amyloid PET scan results
- All AEs that occurred within 48 hours of IMP administration
- All SAEs that occurred within 30 days of IMP administration

If results of any of the following tests are available at this visit, the results will be reviewed with the participant/caregiver and recorded in the eCRF:

- FDG-PET
- Lumbar puncture
- Optional blood tests
- Single photon emission computed tomography (SPECT)
- Optional neuropsychiatric testing
- Other tests

9.5 V1 at T2, 6 Months (±14 days) After Baseline

A clinic visit (V1) is scheduled for all participants at T2, which is defined as 6 months (±14 days) after baseline. If the managing physician chooses to order amyloid PET imaging for a member of the Free Choice arm, the imaging may be performed at any time from baseline to 12 months after baseline.

The following will be recorded for all participants who have undergone amyloid PET imaging:

- Amyloid PET scan results
- All AEs that occurred within 48 hours of IMP administration
- All SAEs that occurred within 30 days of IMP administration

At the clinic visit at 6 months (±14 days) after baseline, the following will be recorded for all participants:

- A review of current medications
- Cognition/functioning (episodic memory, verbal memory, visual/spatial function, executive function)

- MMSE®
- EQ5D-5L
- ICECAP-O
- Brief COPE – Brief Coping Orientation to the Problems Experienced
- Hospital Anxiety and Depression (HAD) scale
- Use of medical resources, as collected in the patient questionnaire
- Syndromic diagnosis made by the managing physician
- Maximum 4 hypotheses of etiologic diagnosis made by the managing physician, each with a diagnostic confidence (only if the diagnostic confidence >90% is not achieved for one etiologic diagnosis hypothesis)
- Diagnostic confidence (the managing physician's confidence that the diagnosis is correct) for each hypothesis.
- Management plan made by the managing physician
- The managing physician's estimate of the likelihood that the patient's symptoms are due to AD.

If results of any of the following tests are available at this visit, the results will be reviewed with the participant/caregiver and recorded in the eCRF:

- FDG-PET
- Lumbar puncture
- Optional blood tests
- Single photon emission computed tomography (SPECT)
- Optional neuropsychiatric testing
- Other tests

9.6 Amyloid PET for Late Amyloid PET Arm, 8 Months (±8 Weeks After Baseline)

All female participants of childbearing potential must have a negative pregnancy test before the imagine study is done. The participants in the Late Amyloid PET arm of the study will undergo amyloid PET imaging at 8 months (±8 weeks) after baseline. Amyloid PET scan must take place after the T2 visit at 6 months +/- 14 days.

If the managing physician chooses to order amyloid PET imaging for the participants in the Free Choice arm, those participants may undergo the imaging at any time from baseline to 12 months after baseline.

The following will be recorded for all participants who have undergone amyloid PET imaging:

- Review of current medications
- Amyloid PET scan results
- All AEs that occurred within 48 hours of study drug administration
- All SAEs occurring within 30 days of study drug administration

9.7 Optional V2 at T3, 13 Months (±4 weeks) After Baseline

If the managing physician orders amyloid PET imaging for a member of the Free Choice arm, the imaging can be performed at any time from baseline to 12 months after baseline. At the optional V2 visit at T3, which is defined as 13 months (±4 weeks) after baseline, the following will be recorded for all participants who have undergone amyloid PET imaging:

- Amyloid PET scan results
- All AEs that occurred within 48 hours of IMP administration
- All SAEs that occurred within 30 days of IMP administration

At the clinic visit at T3, the following will be recorded for all participants:

- A review of current medications
- Cognition/functioning (episodic memory, verbal memory, visual/spatial function, executive function)
- MMSE
- EQ5D-5L
- ICECAP-O
- Brief COPE – Brief Coping Orientation to the Problems Experienced
- Hospital Anxiety and Depression (HAD) scale
- Use of medical resources, as collected in the patient questionnaire
- Syndromic diagnosis made by the managing physician
- Maximum 4 hypotheses of etiologic diagnosis made by the managing physician, each with a diagnostic confidence (only if the diagnostic confidence >90% is not achieved for one etiologic diagnosis hypothesis)
- Diagnostic confidence (the managing physician's confidence that the diagnosis is correct) for each hypothesis
- Management plan made by the managing physician
- The managing physician's estimate of the likelihood that the patient's symptoms are due to AD.

If results of any of the following tests are available at this visit, the results will be reviewed with the participant/caregiver and recorded in the eCRF:

- FDG-PET
- Lumbar puncture
- Optional blood tests
- Single photon emission computed tomography (SPECT)
- Optional neuropsychiatric testing
- Other tests

9.8 Study Completion

Visit 2 must be completed within 13 months (±4 weeks) of the baseline visit. Participants who do not complete visit 2 within this period will not be replaced.

9.9 T4: Follow-up Visit for Participants in Early Amyloid PET Arm

In a follow-up assessment, the participants who were randomized to the Early Amyloid PET arm will receive an optional second amyloid PET imaging scan 12 to 18 months after the first scan. All female participants of childbearing potential must have a negative pregnancy test before the imaging study is done. Concurrent medications will be reviewed before imaging.

The following will be recorded within 28 days after the optional second scan, for all participants who have undergone the second amyloid PET scan:

- Amyloid PET scan results
- All AEs that occur within 48 hours of study drug administration
- All SAEs occurring within 30 days of study drug administration

The following will be recorded for all participants in the Early Amyloid PET arm:

- Managing physician's etiologic diagnosis after review of amyloid PET imaging results
- Managing physician's management plan after review of amyloid PET imaging results

10 EFFICACY, SAFETY, AND OTHER VARIABLES

10.1 Efficacy Assessments

10.1.1 Primary endpoint

The difference, at 12 weeks after baseline, between the Early Amyloid PET arm and the Late Amyloid PET arm in the proportion of patients for whom the managing physician has made an etiologic diagnosis with very high confidence (≥90%). Diagnostic confidence, which is the managing physician's confidence that the diagnosis is correct, will be measured with a VAS from 0% (no confidence) to 100% (complete confidence).

10.1.2 Secondary endpoints

The timepoints are defined as follows:

- T0 = baseline,
- T1 = 12 weeks after T0,
- T2 = 6 months (±14 days) after T0,
- T3 = 13 months (±4 weeks) after T0;
- T4 = ≤28 days after the optional second scan, which will be 12 to 18 months after T0.

10.1.2.1 Diagnosis and confidence

- The difference between the Early Amyloid PET arm and Late Amyloid PET arm in the time to communicate to the patient an etiologic diagnosis with very high confidence (≥ 90%).
- The changes in the managing physician's etiologic diagnosis at T3 vs T2 vs T1 in each arm.
- The changes in the managing physician's diagnostic confidence at T3 vs T2 vs T1 vs T0 in the Early Amyloid PET vs Late Amyloid PET arms.
- The managing physician's estimate of the likelihood that the patient's symptoms are due to AD at T3 vs T2 vs T1 vs T0 in the Early Amyloid PET arm vs the Late Amyloid PET arm.
- Changes over calendar time in the placement of amyloid PET imaging in the patient workup for participants in the Free Choice arm.

10.1.2.2 Patient management

- The difference between arms (Early Amyloid PET arm, the Late Amyloid PET arm, or the Free Choice arm) in the number of patients randomised to disease-modifying drug (DMD) or any other AD clinical trial at T2.
- The difference between the Early Amyloid PET arm and the Late Amyloid PET arm in number of participants with changes in the management plan (changes in or start of a new program or pharmacologic treatment) at T1 vs T2 vs T3.

10.1.2.3 Health economics

- The impact on patient-related outcomes (cognition, anxiety, depression, coping skills, and quality of life) at T3 vs T2 vs T0 in each arm

- The difference in the cost of diagnostic workup to the etiologic diagnosis with very high confidence (≥90%) in the Early Amyloid PET Imaging arm vs the Late Amyloid PET Imaging arm. (In those centres where the subjects in the Early Amyloid PET Imaging arm receive a diagnostic workup in addition to clinical evaluation before baseline, the relative costs will not be considered if the results of those exams are not known by the managing physician before the managing physician makes the etiologic diagnosis.)
- Differences in the use of medical resources (not limited to diagnostic procedures, tests, visits, and hospitalisations) and programs between Early Amyloid PET and Late Amyloid PET arms.
- The number of patients who withdraw from the study and the reasons for withdrawal.

10.1.2.4 Quantitative imaging results assessment

In addition to the local visual read of the PET image used for the primary and secondary (diagnosis and confidence) endpoints, PET images will be analysed centrally by the Sponsor for quantitative imaging endpoints, as follows.

For all subjects (initial amyloid PET scan only):

- The distributions and mean standardised uptake value ratio (SUVR) of the composite cortical brain regions, and converted to the centiloid scale, across amyloid PET tracers and by diagnostic subgroup.
- The distributions and mean SUVR of defined brain regions across amyloid PET tracers and by diagnostic subgroup.
- For each of the amyloid PET tracers, the composite cortical quantitative uptake (SUVR and SUVR converted to the centiloid scale) versus the local site visual reading interpretation and classification confidence.

For subjects in the Early Amyloid PET Imaging arm who have a second amyloid PET scan:

- The distributions and mean change in standardised uptake value ratio (SUVR) of the composite cortical brain regions, and converted to the centiloid scale, across amyloid PET tracers and by diagnostic subgroup.
- The distributions and mean change in SUVR of defined brain regions across amyloid PET tracers and by diagnostic subgroup.

10.1.3 Image acquisition

The instructions for acquiring the amyloid PET images are provided in the Imaging Manual.

NeuraCeq, Vizamyl and flutemetamol (^{18}F) images should be acquired starting at 90 minutes after intravenous injection. The scan duration should typically be 20 minutes.

10.1.4 Image storage

The contract research organization IXICO will be hosting image storage (NeuraCeq, Vizamyl and flutemetamol [^{18}F] images) on their Trial Tracker platform for this study.

10.1.5 Image interpretation

The local site interpretation of the amyloid PET images will be performed by readers who have undergone the tracer-specific reader training provided by Piramal (for NeuraCeq™) and GEHC (for Vizamyl™) and training in the eCRF completion.

For information about image interpretation, see the accompanying Imaging manual.

10.1.6 PET data reporting

Electronic case report forms that are specific to the reading methodology for the given tracer will be used by the investigator to record the results of the amyloid PET scan, classification as positive or negative and a rating of classification certainty.

10.2 Safety Assessments

The investigator(s) and the Sponsor will review the safety data. The following will be recorded:

- Conditions that started before the informed consent form was signed will be recorded as medical/surgical history.
- Adverse events (AEs) that occur within 48 hours of study drug administration will be recorded.
- SAEs occurring within 30 days of study drug administration will be recorded.

10.2.1 Treatment-emergent adverse events

AE Definition: An AE is defined as any untoward medical occurrence in a patient or clinical investigation participant administered a pharmaceutical product and which does not necessarily have to have a causal relationship with this treatment. An AE can therefore be any unfavourable and unintended sign (including an abnormal laboratory finding), symptom, or disease temporally associated with the use of an IMP, whether or not considered related to that product. Only symptoms/signs that begin or worsen in severity and/or frequency after IMP administration/use will be recorded as AEs in the eCRF.

The participants will be closely observed and questioned for any kind of AE during the study procedures and at follow-up appointments throughout the study period with non-leading questioning (e.g., “How do you feel?”). The participants will be instructed to immediately report any symptoms and signs to the study staff (i.e., between formal observations).

All AEs that occur within 48 hours of study drug administration will be recorded.

Both the investigator(s) and Sponsor will perform a causality assessment on any AE, to assess whether or not there is a reasonable possibility (evidence to suggest) that the IMP caused the event.

Adverse Reaction: An AE that is caused by the IMP.

Suspected Adverse Reaction: A reasonable possibility exists for causality between the IMP and the AE

10.2.1.1 Expected adverse events

The reference safety information for the determination of adverse drug reactions will be the SmPC for florbetaben (^{18}F) [NeuraCeq™ SmPC], the SmPC for Vizamyl™ [Vizamyl SmPC], and the Investigator Brochure for Flutemetamol (^{18}F) Injection [[18F Flutemetamol].

10.2.1.2 Expected conduct-related AEs

The use of an indwelling venous cannula for the administration of study drug may be accompanied by mild bruising and also, in rare cases, by transient inflammation of the vessel wall. After initial irritation, the presence of an indwelling cannula is usually painless and hardly noticeable. The same applies to single vein punctures for blood sampling.

10.2.1.3 Expected adverse drug reactions

Please see the SmPC for the possible side effects of NeuraCeq™ [NeuraCeq™ SmPC] and Vizamyl™ [Vizamyl SmPC] and the Investigator Brochure for Flutemetamol (^{18}F) Injection [[18F Flutemetamol].

These radiopharmaceuticals will deliver low amounts of ionizing radiation associated with the least risk of cancer and hereditary abnormalities.

However, AEs from the radioactive dose are not expected, since the applied radiation doses are far below doses that can cause acute effects in human tissues.

10.2.1.4 Precautionary measures

Special precautionary measures are not considered to be necessary for this study. In case of emergency, standard emergency procedures will be employed.

10.2.1.5 Unexpected adverse drug reactions

An unexpected adverse drug reaction is defined as an adverse reaction in the nature and severity which is not consistent with the applicable product information (e.g., in the SmPC or in the IB). Any adverse experience that is not listed in the current SmPC or IB with regard to the specificity or severity shall be regarded as unexpected. “Unexpected” as used in this definition refers to an adverse drug experience that has not been previously observed and included in the product information, rather than from the perspective of such experience not being anticipated from the pharmacological properties of the investigational product.

10.2.2 Serious adverse events

An SAE is defined as any AE that:

- Results in death.
- Is life threatening.
- Requires in-patient hospitalisation or prolongation of existing hospitalisation.

- Results in persistent or significant disability or incapacity.
- Is a congenital anomaly or birth defect.
- Is another important medical event.*

(*Other important medical events are those that may not result in death, be life threatening, or require hospitalisation, but may be considered an SAE when, based upon appropriate medical judgment, they may jeopardise the participant and may require medical intervention to prevent one of the outcomes listed above.

All SAEs occurring within 30 days of study drug administration will be recorded.

10.2.3 Adverse event and serious adverse event reporting

All AEs should be recorded using acceptable diagnoses, if possible. If an AE has already been reported it is not necessary to report each individual sign and symptom of that AE as a separate AE. For example, if myocardial infarction is reported as an AE, there is no need to report elevated creatine kinase and abnormal ECG, or other related signs, symptoms, or laboratory values as separate AEs. However, if both occurred in isolation and myocardial infarction was not diagnosed, then each event would be reported as an AE.

The intensity of all AEs will be graded as mild, moderate, or severe using the following definitions:

Mild:	Tolerable.
Moderate:	Interferes with normal activity.
Severe:	Incapacitating (causes inability to perform usual activity or work).

The investigator will be instructed to closely monitor each participant who experiences an AE (whether ascribed to the IMP or not) until the outcome of the AE has been determined.

In addition to the investigator's own description of the AEs, each AE will be encoded by the Sponsor/CRO according to a well-recognised dictionary of medical codes.

SAEs will be recorded in the eCRF if they occurred as follows:

- After a participant first received an IMP and throughout the participant's follow-up period*, whether or not considered related to the IMP, and
- After the participant's follow-up period, and for which a causal relationship to the IMP cannot be ruled out.

(*Follow-up period is defined as the protocol-stipulated period or, for participants prematurely withdrawn from a study, the duration of a participant's participation.)

All serious and non-serious AEs must be followed for a final outcome until the end of the follow-up period. An outcome of "unknown" is not considered to be an acceptable final outcome. An outcome of "not yet resolved" is an acceptable final outcome for non-serious AEs at the end of a participant's participation in a study, and for SAEs at database lock.

The investigator should take appropriate diagnostic and therapeutic measures to minimise the risk to the participant. Where appropriate he/she should take diagnostic measures to collect evidence for clarification of the relationship between the SAE and the study drug.

The investigator must submit a complete “Serious Adverse Event Report” for all SAEs, regardless of a possible causal relationship. All reports of SAEs (initial and follow-up) must be reported to drug safety manager of the Sponsor (see Investigator’s Trial File and described in detail in the safety management plan for the trial) immediately (at the latest within 24 hours of having gained knowledge of the event).

The initial report should be as complete as possible; subsequent minor corrections or additions may be reported by re-submitting the amended initial SAE report. If relevant further information or information about the outcome becomes available, "Follow-up Reports" must be completed and sent to the Sponsor’s drug safety department. For all SAEs, the investigator is required to document in full the course of the SAE and any therapy given, including any relevant findings/records in the report.

All SAEs must also be recorded on the ‘Adverse Event Form’ of the eCRF.

Participants enrolled will be provided with a Clinical Trial Participant card at the time of IMP administration. This card will list contact details for the investigator.

10.2.4 Urgent safety measures

In accordance with the principles of GCP as laid out in ICH E6, the investigator(s) has/have primary responsibility for assuring participant safety throughout the performance of study procedures. An urgent safety measure is defined as any measure which an investigator may need to implement which is a deviation from, or a change in, the protocol to eliminate an immediate hazard(s) to trial participants without prior IEC/IRB approval/favourable opinion.

The investigator may take appropriate urgent safety measures to protect the participants of a clinical trial against any immediate hazards to their health or safety. However, the investigator must inform the Sponsor/CRO within 24 hours of having taken such measures.

The Sponsor shall immediately, and in any event, no later than 3 days from the date the measures are taken, give written notice to the licensing authority and the relevant IEC/IRB of the measures taken and the circumstances giving rise to those measures.

All urgent safety measures must be reported to the Sponsor/CRO by using the SAE contact numbers provided in the Investigator’s Trial File within 24 hours of having to take such a measure(s). Such reports can be initiated by telephone but must be officially documented by the investigator (by email or fax) and must include details of what measures were taken and the circumstances giving rise to those measures.

10.2.5 Pregnancy reporting

This process is aimed at ensuring the appropriate monitoring of the potential risk related to IMP exposure of pregnant women and/or foetuses as well as the risks associated with exposure

of a father, regarding congenital abnormalities or birth defects in their offspring. It also ensures compliance with applicable international and local regulations.

The requirements are applicable to all participants following exposure to IMP.

Female trial participants: The trial participant must be advised by the investigator to inform the investigator immediately if the participant suspects she may be pregnant. Any pregnancy that occurs within 72 hours after administration of the IMP should be reported to the investigator.

Male trial participants: The trial participant must be advised by the investigator to inform the investigator immediately if the participant suspects that his partner became pregnant after the he was administered with IMP. Any pregnancy that occurs in a partner of the male participant within 72 hours after administration of the IMP should be reported to the investigator.

When a trial participant reports a pregnancy (post-IMP administration) to the investigator, a pregnancy test should be arranged for the trial participant (or his partner) by the investigator within 7 days of the pregnancy being reported.

The investigator must inform the Sponsor within 24 hours of receiving positive pregnancy test results by using either a copy of the relevant CRF page (demography or AE) or via email. The investigator should include an estimated date of conception when communicating with the Sponsor/CRO.

10.3 Other Variables

10.3.1 Demographic data

The following demographic data will be recorded during the baseline visit:
- Age
- Height
- Weight
- Sex
- Race

10.3.2 Medical and surgical history

The medical and surgical history will be recorded during the screening visit.

10.3.3 Pre-treatment events

Events that occur before administration of IMP will be recorded as medical or surgical history in the eCRF.

The following information on pre-treatment events will also be recorded in the eCRF:

- The onset time
- Action taken

- Status
- Intensity

10.3.4 Prior and concurrent medication

Prior and concurrent medications will be recorded during the screening visit, at the time of imaging, at T2, and at T3.

10.3.5 Vital signs

Vital signs (blood pressure, heart rate, respiratory rate, temperature, respiratory rate) will be monitored at the screening visit (see Table 1).

10.3.6 Physical examination

The managing physician will conduct physical examinations during the screening visit. The physical examination and neurologic examination will include recording an assessment as needed for determination of the inclusion/exclusion criteria.

10.3.7 Cognition/functioning

The investigator can use the neuropsychological tests that are normally used in each centre. Data on each participant's cognition/functioning (episodic memory, verbal memory, attention, verbal fluency, visual/spatial function, executive function) will be recorded at T0, T2, and T3.

10.3.8 MRI and CT results

As part of the inclusion criteria, the participant must have undergone MRI and CT imaging up to 12 months before the screening visit, to rule out intracranial masses or other lesions as causes of their symptoms. The results of this MRI and CT imaging will be recorded in the eCRF at T0, after the Informed Consent form has been signed.

10.3.9 Mini-Mental State Examination

The score of the Mini-Mental State Examination at T0, T2, and T3 will be recorded in the eCRF.

10.3.10 ICECAP-O (ICEpop CAPability measure for Older people)

The score of the ICECAP-O at T0, T2, and T3 will be recorded in the eCRF.

10.3.11 Brief-COPE

The score of the Brief-COPE at T0, T2, and T3 will be recorded in the eCRF.

10.3.12 EQ-5D-5L (5 level-EQ-5D)

The score of the EQ-5D-5L at T0, T2, and T3 will be recorded in the eCRF.

10.3.13 Hospital Anxiety and Depression (HAD)

The score of the HAD scale at T0, T2, and T3 will be recorded in the eCRF.

10.3.14 Patient questionnaire (use of medical resources)

Data from the patient questionnaire on the use of medical resources will be collected at T0, T1, T2, and T3 and recorded in the eCRF. (T1 does not require a clinic visit.)

In those centres where the participants in the Early Amyloid PET arm receive a diagnostic workup in addition to clinical evaluation before baseline, the relative costs will not be considered if the results of those exams are not known by the managing physician before the managing physician makes the etiologic diagnosis.

10.3.15 Syndromic diagnosis

The syndromic diagnosis made by the managing physician (see section 7.5) at T0, T1, T2, and T3 will be recorded at in the eCRF.

10.3.16 Etiologic diagnosis

The etiologic diagnosis made by the managing physician at T1, T2, and T3 will be recorded in the eCRF. Earlier on in the workup, when diagnostic hypotheses are made, up to 4 diagnostic hypotheses will be recorded.

10.3.17 Management plan

The management plan (pharmacologic and nonpharmacologic) made by the managing physician at T1, T2, and T3 will be recorded in the eCRF. Pharmacologic management may include cognitive enhancers (acetylcholinesterase inhibitors, memantine, ginkgo biloba, and others [including nutraceuticals]), selective serotonin reuptake inhibitors, benzodiazepines, hypnotics, and neuroleptics (typical and/or atypical). Nonpharmacologic management may include cognitive rehabilitation, validation therapy, psychoeducation of family members, nutritional intervention, physical activity programmes, occupational therapy, and others.

10.3.18 Diagnostic confidence

The managing physician's diagnostic confidence, as measured by a Visual Analogue Scale (0% meaning zero confidence in the diagnosis and 100% meaning complete confidence that the diagnosis is correct) at T0, T1, T2, and T3 will be recorded in the eCRF.

10.3.19 The managing physician's estimate of the likelihood that the patient's symptoms are due to AD

The managing physician will be asked to evaluate, in percentage (0% to 100%), the likelihood that the patient's symptoms are due to AD.

10.3.20 Amyloid PET scan results

The results of the amyloid PET scan will be recorded in the eCRF after the scan. The results of the visual reading will be recorded as positive or negative for the overall read. The timing of the scan is different for the different arms of the main study.

- **Early Amyloid PET arm:** participants will undergo amyloid PET scanning within 4 weeks of baseline. The participants in this arm will undergo an optional second scan 12 to 18 months after the first scan.
- **Late Amyloid PET arm:** participants will undergo amyloid PET scanning at 8 months ±8 weeks.
- **Free Choice arm:** participants may or may not undergo amyloid PET scanning, according to the managing physician's decision. If they undergo scanning, the scan may take place at any time within 12 months after baseline.

10.3.21 FDG-PET

FDG-PET imaging is not required under this protocol. If a participant undergoes FDG-PET scanning during this study, the results will be recorded in the eCRF.

10.3.22 SPECT imaging

SPECT imaging is not required under this protocol. If a participant undergoes SPECT imaging of the brain during this study, the results will be recorded in the eCRF.

10.3.23 Cerebrospinal fluid (CSF) analysis

Analysis of CSF is not required under this protocol. If such analysis is performed during this study, the results will be recorded in the eCRF.

10.3.24 Blood tests

No blood testing is required under this protocol. The results of any blood testing (including the dementia blood workup performed before participation in this study) that is performed for participants during this study will be recorded in the eCRF.

10.3.25 Optional neuropsychological testing

During this study, participants may undergo neuropsychological testing that is not required under this protocol. The results of any such testing will be recorded in the eCRF.

10.3.26 Other data

Other data that is collected from the participants during this study may be recorded in the eCRF.

10.4 Appropriateness of Measurements

All clinical assessments and measurements are appropriate and generally regarded as standard medical practice.

11 DATA HANDLING AND QUALITY ASSURANCE

11.1 Data Recording

Data required per this protocol are to be entered into the eCRFs (provided by the Sponsor) as soon as possible; it is expected that will be within 5 days after the information becomes available.

11.2 Electronic Case Report Forms

eCRFs will be used for collecting all data generated during the study, including the diagnostic findings from the PET read.

All data found in the eCRF will be transcribed from source documentation maintained by the Principal Investigator or designee. All data will be entered into the eCRF by trained site personnel, with reasons given for any missing data. Any errors should be corrected within the electronic system. The audit trail will record all changes made, the date and time of the correction, and the person correcting the error.

11.3 Clinical Data Management

The Sponsor or a qualified delegate (ie, a contract research organization [CRO]) will be responsible for the processing and quality control of the data. Data management will be carried out by the Sponsor or CRO. The handling of data, including data quality control, will comply with all applicable regulatory guidelines.

11.4 Data Processing

Study data documentation will be maintained specifying all relevant aspects of data processing for the study (including data validation, cleaning, correcting, releasing). This documentation will be stored in the Trial Master File (TMF).

For data coding, internationally recognised and accepted dictionaries will be used. These and the processes used for coding will be specified in the SAP.

11.5 Archiving

Essential documents will be archived safely and securely in such a way that ensures that they are readily available upon Regulatory Authorities' request. Clinical study documentation must be maintained for at least 15 years after the end of the study or its anticipated stop, or longer if this is anticipated by a contract between the Sponsor and investigator.

The investigator/institution must notify the Sponsor if the archival arrangements change (e.g., relocation or transfer of ownership).

The investigator site file is not to be destroyed without the approval of the Sponsor.

11.6 Data Pseudonymization

Data on the eCRF will be coded and each patient will be identified by PID (see Section 8.3.1).

A patient log (i.e a file that includes the correspondence between the PID and patients' name and Date of Birth) will be maintained for all the duration of the study, and for at least 15 years after the end of the study, as specified in national legal regulations.

The access to and the maintenance of this file will be under the responsibility of the PI.

12 STATISTICAL METHODS AND PLANNED ANALYSIS

The data will be analysed by the Sponsor and/or designated CRO. Any data analysis carried out independently by the investigator should be submitted to the Sponsor before publication or presentation.

Data from participating centres in this protocol will be combined so that an adequate number of participants will be available for analysis. The data will be summarised with respect to demographic and baseline characteristics, efficacy observations and measurements, and safety observations and measurements.

12.1 General Statistical Considerations

A separate Statistical Analysis Plan (SAP) will be prepared before the start of the statistical analysis to provide further details on the statistical analysis and formalise data outputs from this clinical study.

Tabulations of summary statistics, graphical presentations, and statistical analyses will be performed with SAS® software, Version 9.2 or higher. Descriptive statistics for continuous data in summary tables will include the number of participants in the analysis (n), mean, standard deviation, median, and range (minimum, maximum). Descriptive statistics for categorical data in summary tables will include counts and percentages.

All data obtained on the eCRF and entered into the database will be provided in separate data listings showing individual participant values. All summary tables and data listings will be separated by treatment groups. The planning and reporting of statistical analysis will be carried out as described in the Sponsor or CRO's standard operating procedures (SOPs) governing clinical studies.

12.2 Populations for Analysis

Safety Analysis Set (SAS): All participants receiving any amount of either [^{18}F]florbetaben or [^{18}F]flutemetamol are included in the SAS.

Full Analysis Set (FAS): All participants randomized to the Early Amyloid PET, Late Amyloid PET, or Free Choice arm.

Per Protocol Set (PPS): All participants randomized to the Early Amyloid PET, Late Amyloid PET, or Free Choice arm with no major protocol deviations that would make them ineligible for the primary efficacy variable. Participants who are lost to follow-up will be excluded from the PPS.

The primary efficacy analysis will be based on the PPS population, and the secondary efficacy analysis will be based on the FAS population.

12.3 Participant Demographics/Other Baseline Characteristics

A table will be provided with the following information:

- Number of participants enrolled.
- Number of participants included in the Full Analysis Set (FAS).
- Number of participants included in the Safety Analysis Set (SAS)
- Number of participants included in the Per Protocol Set (PPS)
- Number of participants withdrawn from the study and the reason for withdrawal.

For SAS, FAS, and PPS, demographic information (age, height, weight, and body mass index) will be summarised with descriptive statistics. Gender and race will be summarised by counts and percentages. Medical histories will be summarised by counts and percentages. Concurrent medications will be recorded and coded by using a standard classification system and grouped by primary and secondary classes, if applicable. The primary efficacy analysis will be based on the PPS population, and the secondary efficacy analysis will be based on the FAS population.

12.3.1 Physical examination findings

A physical examination will be performed during screening.

12.3.2 Vital signs

Vital signs will be recorded at screening.

12.4 Study Treatments

12.5 Primary Analysis

12.5.1 Primary Efficacy Variables

The primary variable will be the difference, at 12 weeks after baseline, between the Early Amyloid PET arm and the Late Amyloid PET arm in the proportion of patients for whom the managing physician has made an etiologic diagnosis with very high confidence (≥90%).

Diagnostic confidence, which is the managing physician's confidence that the diagnosis is correct, will be measured with a visual analogue scale (VAS) from 0% (no confidence) to 100% (full confidence).

12.5.2 Statistical hypothesis, model, and method of analysis

The difference between the Early Amyloid PET and Late Amyloid PET arms in the proportion of patients with an etiologic diagnosis in which the managing physician has ≥90% confidence at 12 weeks (the primary endpoint) will be evaluated with a chi-squared test with significance level of .05.

For each of the three strata (SCD Plus, MCI, and dementia) separately, the difference between the Early Amyloid PET arm and the Late Amyloid PET arm in the proportion of patients with an etiologic diagnosis with a confidence ≥90% at 12 weeks will be evaluated with a chi-squared test with an overall significance level of .05. The Bonferroni correction will be applied to control the family-wise type I error rate.

12.5.3 Handling of missing values/censoring/discontinuations

The reasons for incomplete data are many and include major protocol deviations, dropout because of study withdrawal or death, non-attendance at clinical visits, missing questionnaire items, uninterpretable images, etc.

The primary efficacy analysis will be conducted on the PPS population, which is based on complete cases.

Secondary efficacy analyses will be conducted on the FAS population. In time-to-event analyses, dropout and major protocol deviations are accounted for by censoring at the time of dropout or protocol deviation. In cross-sectional analyses and analyses of change between 2 timepoints, only complete cases will be included. Cases with a major protocol deviation before the first timepoint in the cross-sectional or change analysis will be excluded. The longitudinal mixed-effect analyses will be based on the observed cases. Data will be censored after a major protocol deviation. The underlying assumption in all analyses is that missing data are missing at random and not related to the primary outcome.

The proportion of missing data at each time point will be computed for the Early Amyloid PET, Late Amyloid PET, and Free Choice arms separately, and stratified for SCD Plus, MCI, and dementia.

12.5.4 Supportive analyses

Heterogeneity with respect to the effect on the primary variable between centres will be evaluated with the Breslow-Day test of homogeneity. If this effect varies across centres, centre-specific effects will be calculated.

12.6 Secondary Analyses

12.6.1 Secondary efficacy variables

In the analyses of the secondary efficacy variables, a cross-sectional difference between arms in a secondary endpoint at T1, T2, T3, or T4 (see Table 1) will be carried out by a chi-squared test if the endpoint is dichotomous, a 2-sided *t*-test if the endpoint is normal, and Mann-Whitney test if the endpoint is categorical or continuous and non-normal. The effect of baseline characteristics such as age, gender, MMSE, etc will be studied by a linear or logistic regression model.

A difference between study arms in the longitudinal change of a secondary endpoint will be tested by means of logistic or linear regression where the first measurement is a covariate (ANCOVA analysis) and the second measurement is the outcome. Differences in longitudinal

trends between arms will also be examined by means of the mixed-effects regression model. The effects of baseline characteristics such as age, gender, MMSE, etc. will be studied by including them in the regression models.

All tests will be 2-sided and the significance level will be set at 5%. Bonferroni corrections for multiple testing will be applied when appropriate. Point estimates will be presented together with 95% Wald confidence intervals. A 95% non-parametric confidence interval will be constructed if the estimate is non-normal.

12.6.1.1 Diagnosis and Confidence

- A difference between the Early Amyloid PET arm and the Late Amyloid PET arm in the time to communicate an etiologic diagnosis with a confidence ≥90% will be tested by the log-rank test. The cumulative incidence of diagnoses will be estimated with the Kaplan-Meier method.
- Changes in the managing physician's etiologic diagnosis at T3 vs T2 vs T1 in each arm
- The changes in the managing physician's diagnostic confidence at T3 vs T2 vs T1 vs T0, for participants in the Early Amyloid PET arm vs participants in the Late Amyloid PET arm
- The managing physician's estimate of the likelihood that the patient's symptoms are due to AD at T3 vs T2 vs T1 vs T0 in the Early Amyloid PET arm vs the Late Amyloid PET arm.
- Changes over calendar time in the placement of amyloid PET imaging in the patient workup for participants in the Free Choice arm. The Free Choice arm will be divided into 3 equal-sized groups, according to the date of randomisation. For the Free Choice arm, a change in the placement of amyloid PET imaging over time in the diagnostic workup will be tested by a log-rank test.

12.6.1.2 Patient Management

- The difference between arms (Early Amyloid PET arm, the Late Amyloid PET arm, or the Free Choice arm) in the number of patients randomised to disease-modifying drug (DMD) or any other AD clinical trial at T2.
- The difference between the Early Amyloid PET arm and the Late Amyloid PET arm in number of participants with changes in the management plan (changes in or start of a new program or pharmacologic treatment) at T1 vs T2 vs T3.

12.6.1.3 Health Economics Objectives

- The impact on patient-reported outcomes (cognition, anxiety, depression, coping skills, and quality of life) at T3 vs T2 vs T0 in each arm
- The difference in the cost of diagnostic workup to the etiologic diagnosis with very high confidence (≥90%) in the Early Amyloid PET arm vs the Late Amyloid PET arm. (In those centres where the participants in the Early Amyloid PET arm receive a diagnostic workup in addition to clinical evaluation before baseline, the relative costs will not be considered if the results of those exams are not known by the managing physician before the managing physician makes the etiologic diagnosis.)
- The difference between arms (Early Amyloid PET vs Late Amyloid PET vs Free Choice) in the use of medical resources (not limited to diagnostic procedures, tests, visits, and hospitalisations) and programs.

- The number of patients who withdrew from the study, and the reasons for withdrawal.

12.6.1.4 **Quantitative Imaging Results Assessment**

PET images will be analysed centrally for quantitative imaging endpoints, as follows:

- Descriptive analysis of local visual assessment results
- The distributions and mean standardised uptake value ratio (SUVR) of the composite cortical brain regions, and converted to the centiloid scale, across amyloid PET tracers and by diagnostic subgroup.
- The distributions and mean SUVR of defined brain regions across amyloid PET tracers and by diagnostic subgroup.
- For each of the amyloid PET tracers, the composite cortical quantitative uptake (SUVR and SUVR converted to the centiloid scale) versus the local site visual reading interpretation and classification confidence.

For participants in the Early Amyloid PET Imaging arm who have a second amyloid PET scan:

- The distributions and mean change in standardised uptake value ratio (SUVR) of the composite cortical brain regions, and converted to the centiloid scale, across amyloid PET tracers and by diagnostic subgroup.
- The distributions and mean change in SUVR of defined brain regions across amyloid PET tracers and by diagnostic subgroup.
- The difference in amyloid load between the first and the second amyloid PET scan, as indicated by quantitative image assessments, will be tested with a paired data *t*-test. Data will be log-transformed if non-normal.

A SCD-specific sub-study aiming at investigating the impact of PET-amyloid result disclosure will be performed on the SCD participants. This specific protocol is submitted separately as an AMYPAD DPMS sub-study.

12.6.2 Safety variables and analyses

12.6.2.1 Adverse events

- The number and percentage of participants with 1 or more AEs within 48 hours of drug administration will be summarised for the Safety Analysis Set and separately by gender, age, study arms, and strata (SCD plus, MCI, and dementia) and by IMP ([^{18}F]florbetaben vs [^{18}F]flutemetamol).
- A difference among the 3 study arms and between Early and Late Amyloid PET arms in number of AEs at 48 hours after IMP administration will be tested by means of a chi-squared test with significance level of .05. The analysis will be repeated for the safety end-point SAEs at 30 days after IMP administration.

12.6.2.2 Serious adverse events

The number and percentage of participants with 1 or more SAEs will be summarised for the Safety Analysis Set.

12.7 Sample Size Calculation

For the primary efficacy analysis, a sample size of 300 per arm yields more than 99% power when a difference in proportion of 25% is assumed and 10% of the participants are assumed to withdraw before the end-point is reached. For difference in proportion of values of 20%, 15%, and 10%, the power is 99.6%, 93%, and 61%, respectively. For each of the three strata (SCD Plus, MCI, and dementia), a stratum sample size of 100 per arm yields 80% power when the difference is 25% and 10% of the patients withdraw before the end-point is reached.

12.8 Procedures for Missing, Unused and Spurious Data

Missing values will not be substituted by estimated values, but treated as missing in the statistical evaluation. All data from all participants dosed in the study will be included in all listings, plots, summary tables, and statistical analyses when appropriate.

12.9 Interim Analysis

No Interim Analysis is planned for this study.

12.10 Rules for Excluding Participants from Analysis

All randomized participants will be included in the analyses unless otherwise specified. The Sponsor will make any decisions regarding whether any participants or any individual values belonging to a participant will be excluded from the evaluations when the protocol violation is considered to have a negative impact on the scientific aspects and interpretation of the study results. Such judgments should be made in a blinded fashion before database lock and before any analyses have been performed. If the participant has received any IMP, all available safety data will be used. The reason(s) for any exclusion will be described in the report.

12.11 Procedures for Reporting Deviations from Original Statistical Plan

Any deviations from the statistical analysis outlined in this protocol will be described, and reasons for the deviations listed, in the final Clinical Study Report.

13 SPECIAL REQUIREMENTS AND PROCEDURES

13.1 Regulatory, Institutional and Ethical Review

Before starting this study, the protocol (authorised by the Sponsor) will be submitted to the regulatory bodies/local health authorities (in accordance with local regulations) and to the IEC/IRB for evaluation. The protocol will also be signed by the principal investigator before submission to the IEC/IRB. The study will not start before the IEC/IRB gives written approval or a favourable opinion in accordance with ICH E6-GCP and all applicable regulatory bodies/local health authorities give approval or a favourable opinion as required.

No changes from the final approved (authorised) protocol will be initiated without the IEC's/IRB's prior written approval or favourable opinion of a written amendment, except when necessary to eliminate immediate hazards to the participants or when the change involves only logistics or administration. The Sponsor will authorise and the principal investigator(s) will sign the protocol amendment prior to submission to the IEC/IRB. Protocol amendments should be submitted to the IEC/IRB without delay.

13.2 Ethical Considerations

To better evaluate the benefit of an early beta amyloid PET imaging in patients with SCD Plus, MCI, and dementia, the managing physician will be blinded to the other diagnostic test results for the Early Amyloid PET arm until their amyloid PET imaging results are available.

13.3 Investigator's Responsibilities

13.3.1 Overall responsibilities

The investigator(s) is/are responsible for conducting the study in full accordance with the Protocol and the Declaration of Helsinki, the *Good Clinical Practice: Consolidated Guideline*, approved by the ICH, and any applicable national and local laws and regulations. Information regarding any investigational centres participating in this study that cannot comply with these standards will be documented.

By signing the Investigator Signature Page of this protocol, the Investigator confirms in writing that he/she has read, understands, and will strictly adhere to the study protocol and will conduct the study in accordance with ICH Harmonised Guidelines for GCP and applicable regulatory requirements.

The investigator may not modify or alter the procedures described in this protocol. If protocol modifications are necessary, all alterations that are not solely of an administrative nature require a formal protocol amendment (see Section 13.4.1 for the involvement of IRB(s)/IEC(s).

If an investigator has deviated from the protocol in order to eliminate an immediate hazard to participants or for other inevitable medical reasons, the investigator shall document all such deviations, including the reasons thereof, and submit the document to the Sponsor and the head of the medical institution as applicable.

13.4 Ethical and Legal Aspects

13.4.1 Ethical and legal conduct of the study

The planning and conduct of this clinical study are subject to national laws. Only when all of the requirements of the appropriate regulatory authority have been fulfilled will the study begin. The study will be conducted in accordance with the ethical principles that have their origin in the Declaration of Helsinki and ICH GCP guidelines.

The study will commence only after the protocol has been approved by the appropriate IRB or IEC and written notification of the approval has been received by the Sponsor. The initiation and termination dates will therefore be informed to the local IEC/IRB prior to commencing the study. The investigator may not modify or alter this protocol without first obtaining the written agreement of the Sponsor. All alterations that are not only of administrative nature require a formal protocol amendment and must be approved by the appropriate IRB/IEC prior to implementation, except where immediate implementation in order to eliminate an imminent hazard to the study participant is necessary. All protocol amendments that are agreed upon must be signed and dated by both the Sponsor and the investigator.

Criteria for the premature termination of the entire study or part of it are given in Section 7.6.3.1.

13.4.2 Participant informed consent

Enrolment and consent of participants into the study will be performed according to the national laws and regulations.

The enrolment of participants will be based on the participant's or representative's ability to consent to participate in the study.

All relevant information on the study will be summarised in the study specific participant informed consent form (ICF). A sample study ICF is provided as a document separate to this protocol.

The investigator will explain all relevant aspects of the study to each study participant/legal representative, before his/her entry into the study (i.e., before examinations and procedures associated with selection for the study are performed).

Patients will be also informed that because of the randomisation:

- Participants assigned to the Early Amyloid PET arm will undergo amyloid PET imaging within 4 weeks after baseline. Participants in the Early Amyloid PET arm will undergo amyloid PET imaging again 12 to18 months after the first scan. For participants in the Early Amyloid PET arm who will receive a second scan, a second informed consent has to be provided, and it will need to be signed by the patient or by her/his legally acceptable representative.
- Participants assigned to the Late Amyloid PET arm will undergo amyloid PET imaging at 8 months (±8 weeks) after baseline

- For participants assigned to the Free Choice Amyloid PET arm, the managing physician decides whether and when to order amyloid PET imaging; this imaging can be done at any time within 12 months after baseline.

The investigator will also mention that written approval of the IRB/IEC has been obtained.

Each study participant/legal representative will have ample time and opportunity to ask questions and will be informed about the right to withdraw from the study at any time without any disadvantage and without having to provide reasons for this decision.

Once the participant ICF has been signed both by the participant and/or legal representative and the investigator or his/her designee, a copy of the signed and dated consent document will be provided to the participant and the original will remain in the investigator's study files.

The investigator will document on the eCRF the time and date of obtaining informed consent. In the event that informed consent is obtained on the date that screening study procedures are performed, the study record or study participant's clinical record must clearly show that informed consent was obtained prior to these procedures.

13.4.3 Direct access to source data/documents

The monitor(s), auditor(s), authorised personnel of the Sponsor/CRO, health authority inspector(s) or their agents, and authorised members of IECs/IRBs will be given direct access to source data and documentation (e.g., medical charts/records, laboratory results, printouts, videotapes, etc.) for source data verification, if participant confidentiality is maintained in accordance with local requirements.

13.4.4 Confidentiality regarding study participants

All records identifying the participant will be kept confidential and, to the extent permitted by the applicable laws and/or regulations, will not be made publicly available.

Participant names will not be supplied to the Sponsor. Only the PID number will be recorded in the eCRF, and if the participant name appears on any other document (e.g., pathologist's report), it must be obliterated before a copy of the document is supplied to the Sponsor. Study findings stored on a computer will be stored in accordance with local data protection laws. The participants will be informed in writing that representatives of the Sponsor, IRB/IEC, or regulatory authorities may inspect their medical records to verify the information collected, and that all personal information made available for inspection will be handled in strictest confidence and in accordance with local data protection laws.

If the results of the study are published, the participant's identity will remain confidential.

The investigator will maintain a list of PID numbers to enable participants to be identified, but copies of this list will not be collected by the Sponsor.

Personal medical information may be scrutinised for verifying data recorded in the CRF. This may be done by the monitor(s), properly authorised persons on behalf of the Sponsor, the

quality assurance unit, or regulatory authorities. Personal medical information will always be treated as confidential.

13.5 Protocol Deviations

Any deviation from the protocol when no approved amendment exists must be documented as a protocol deviation and reported per local requirements. If appropriate, corrective and preventative action must be implemented to avoid repetition. Protocol deviations and any potential impact on the study results will be discussed during the reporting of the study.

Waivers or protocol exceptions will not be granted retrospectively by the Sponsor under any circumstances.

13.6 Study Monitoring

This study will be monitored regularly by an appropriately qualified representative from the Sponsor. Monitoring procedures may include one or more visits or calls designed to clarify all prerequisites before the study starts. Interim monitoring visits will take place on a regular basis per a schedule fixed by mutual agreement. During these visits, the representative will check for completion of the entries on the eCRFs, their compliance with the protocol and with GCP, and will compare the eCRF entries with the source data.

The monitor will verify the correct use of the IMPs. IMP will not be supplied to the investigator site prior to a favourable opinion from the Institutional Review Board/Independent Ethics Committee (IRB/IEC) and if applicable the regulatory authority and from the radiation protection authorities.

In addition, the monitor will determine whether all AEs and SAEs have been appropriately reported (including adherence to the time periods required for SAEs).

In addition, yearly progress reports will be sent to the local IECs/IRBs.

13.7 Audit and Inspection

To ensure compliance with GCP and regulatory requirements, a member of the Sponsor’s (or a designated CRO’s) quality assurance unit may arrange to conduct an audit to assess the performance of the study at the investigational site and of the study documents originating there. The investigator/institution will be informed of the audit outcome.

In addition, inspections by regulatory health authority representatives and IRB(s)/IEC(s) are possible. The investigator should notify the Sponsor immediately of any such inspection.

The investigator/institution agree to allow the auditor or inspector direct access to all relevant documents and allocate his / her time and the time of his/her staff to the auditor/inspector to discuss findings and any issues. Audits and inspections may occur at any time during or after completion of the study.

13.8 Financial Disclosure

Each investigator (including principal and any sub-investigators, as well as their spouses and dependent children) who is directly involved in the treatment or evaluation of research participants has to provide a financial disclosure according to all applicable legal requirements. All relevant documentation will be filed in the TMF and/or investigator site file, as appropriate.

13.9 Compensation for Health Damage of Participants / Insurance

The Sponsor maintains clinical trial insurance coverage for this study.

13.10 Publication Policy

The Sponsor is interested in the publication of the results of this study.

Subgroup data and single-centre data shall not be published until the results of the complete study have been published.

All relevant aspects regarding publication will be part of the contract between the Sponsor and the other memory centres. In short, each centre agrees not to individually publish or present the results it obtains before publication of the full study results. The site may, however, upon written notice to the Sponsor participate in a joint, multicentre publication of the study results with other third party principal investigators and/or institutions, provided that the proposed publication is first reviewed by the sponsor. Moreover, the sponsor shall complete its review within thirty (30) days after receipt of any proposed publication (individual or multicenter) from the site. If the sponsor believes that any proposed publication contains any information relating to patentable items, the disclosure of such proposed publication to any third party shall be delayed for up to sixty (60) days to permit the filing of a patent application. Further detail is described in the Sponsor-Investigator agreement to be signed between the Sponsor and each participating centre/site.

The Sponsor has committed on disclosure of information about clinical trials. The information regarding the study protocol is made publicly available on the internet at www.clinicaltrials.gov. This derives from the standards that international medical journal editors have established requiring protocol registration at the outset of the study as a prerequisite of consideration for publication.

14 REFERENCES

[Albert et al, 2011]
Albert MS, DeKosky ST, Dickson D, Dubois B, Feldman HH, Fox NC, et al. The diagnosis of mild cognitive impairment due to Alzheimer's disease: Recommendations from the National Institute on Aging-Alzheimer's Association workgroups on diagnostic guidelines for Alzheimer's disease. Alzheimers Dement. 2011 May;7(3):270-279.

[Beach et al, 2012]
Beach TG, Monsell SE, Phillips LE, Kukull W. Accuracy of the clinical diagnosis of Alzheimer disease at National Institute on Aging Alzheimer Disease Centers, 2005-2010. J Neuropathol Exp Neurol. 2012 Apr;71(4):266-273.

[Curtis et al, 2015]
Curtis C, Gamez JE, Singh U, et al. Phase 3 trial of flutemetamol labeled with radioactive fluorine 18 imaging and neuritic plaque density JAMA Neurol. 2015 Mar;72(3):287-294.

[[^{18}F] Florbetaben IB]
Investigator's Brochure [^{18}F] Florbetaben February 2017

[[^{18}F Flutemetamol]
Investigator's Brochure [^{18}F] Flutemetamol Edition 10, March 2017

[Frisoni et al 2017]
Frisoni GB, Boccardi M, Barkhof F, Blennow K, Cappa S, Chiotis K, et al. Strategic roadmap for an early diagnosis of Alzheimer's disease based on biomarkers. Lancet Neurol. 2017 Aug;16(8):661-676.

[Hornberger et al, 2015]
Hornberger J, Michalopoulos S, Dai M, Andrade P, Dilla T, Happich M. Cost-effectiveness of Florbetapir-PET in Alzheimer's Disease: A Spanish Societal Perspective. J Ment Health Policy Econ. 2015 Jun; 18(2):63-73.

[Jack et al, 2010]
Jack CR, Knopman DS, Jagust WJ, Shaw LM, Aisen PS, Weiner MW, et al. Hypothetical model of dynamic biomarkers of the Alzheimer's pathological cascade. Lancet Neurol. 2010;9(1):119.

[Jack et al, 2013]
Jack CR, Knopman DS, Jagust WJ, et al. Update on hypothetical model of Alzheimer's disease biomarkers. Lancet Neurol. 2013; 12(2):207-216.

[Jessen et al, 2014]
Jessen F, Amariglio RE, van Boxtel M, Breteler M, Ceccaldi M, Chételat G, et al. A conceptual framework for research on subjective cognitive decline in preclinical Alzheimer's disease Alzheimers Dement. 2014 Nov;10(6):844–852.

[Johnson et al, 2013]
Johnson K, Minoshima S, Bohnen N, Donohoe K, Foster N, Herscovitch P, et al. Update on appropriate use criteria for amyloid PET imaging: dementia experts, mild cognitive impairment, and education J Nucl Med 2013 Vol. 54 (7): 1011-1013.

[Morris, 2005]
Morris JC. Early-stage and preclinical Alzheimer disease. Alzheimer Dis Assoc Disord. 2005 Jul-Sept;19(3):163–165.

[McKhann et al, 2011]
McKhann GM, Knopman DS, Chertkow H, Hyman BT, Jack CR Jr, Kawas CH, et al. The diagnosis of dementia due to Alzheimer's disease: Recommendations from the National Institute on Aging and the Alzheimers's Association workgroup. Alzheimers Dement. 2011:1-7

[NeuraCeq™ SmPC]
Neuraceq Summary of Product Characteristics November 2016

[Nordberg 2010]
Nordberg Amyloid Imaging in Early-Detection of Alzheimer's Disease. Neurodegenerative Diseases March 2010 7(1-3):136-138

[Ong et al, 2013]
Ong K, Villemagne VL, Bahar-Fuchs A, Lamb F, Chételat G, Raniga P, et al. ^{18}F-Florbetaben Aß imaging in mild cognitive impairment. Alzheimers Res Ther. 2013;5:4.

[Prince et al, 2015]
Prince M, Wimo A, Guerchet M, Ali GC, Wu YT, Prina M. World Alzheimer Report 2015 The Global Impact of Dementia An Analysis of Prevalence, Incidence, Cost and Trends. London, UK: Alzheimer's Disease International; 2015.

[Rowe and Villemagne, 2013]
Rowe CC, Villemagne VL. Brain amyloid imaging. J Nucl Med Technol. 2013 Mar;41(1):11-8.

[Sabri et al, 2015]
Sabri O, Sabbagh MN, Seibyl J, Barthel H, Akatsu H, Ouchi Y, et al. Florbetaben PET imaging to detect amyloid beta plaques in Alzheimer's disease: Phase 3 study. Florbetaben Phase 3 Study Group. Alzheimers Dement. 2015 Aug;11(8):964-974.

[Serrano-Pozo et al, 2011]
Serrano-Pozo A, Frosch MP, Masliah E, Hyman BT. Neuropathological alterations in Alzheimer disease. Cold Spring Harb Perspect Med. 2011 Sep;1(1):a006189.

[Silverman et al, 2004]
Silverman DH. Brain ^{18}F-FDG PET in the diagnosis of neurodegenerative dementias: Comparison with perfusion SPECT and with clinical evaluations lacking nuclear imaging. J Nucl Med. 2004;45:594-607.

[Sperling et al, 2011]
Sperling RA, Aisen PS, Beckett LA, Bennett DA, Craft S, Fagan AM, et al. Toward defining the preclinical stages of Alzheimer's disease: Recommendations from the National Institute on Aging-Alzheimer's Association workgroups on diagnostic guidelines for Alzheimer's disease. Alzheimers Dement. 2011;7(3):280-292.

[Vlassenko et al, 2011]
Vlassenko AG, Mintun MA, Xiong C, Sheline YI, Goate AM, Genzinger TL, Morris JC. Amyloid-beta plaque growth in cognitively normal adults: Longitudinal [^{11}C]Pittsburgh compound data. Ann Neurol. 2011;70(5): 857–861.

[Villemagne et al, 2011]
Villemagne VL, Ong K, Mulligan RS, Holl G, Pejoska S, Jones G, et al. Amyloid imaging with ^{18}F-florbetaben in Alzheimer disease and other dementias. J Nucl Med. 2011;52(8):1210-1217.

[Vizamyl SmPC]
Vizamyl Summary of Product Characteristics 21 February 2017.
