# Supplementary material for: Clinical Effect of Early vs Late Amyloid Positron Emission Tomography in Memory Clinic Patients: The AMYPAD-DPMS Randomized Clinical Trial
Source: JAMA Neurol. 2023 May 8;80(6):548–57. doi: 10.1001/jamaneurol.2023.0997 (PMC10167601; doi:10.1001/jamaneurol.2023.0997)
Supplement: Supplement 4. — Data sharing statement [file jamaneurol-e230997-s004.pdf]

Data Sharing Statement

Altomare. Clinical Effect of Early vs Late Amyloid Positron Emission Tomography in Memory Clinic Patients. *JAMA Neurol*. Published May 08, 2023. doi:10.1001/jamaneurol.2023.0997

Data
Data available: Yes
Data types: Deidentified participant data, Data dictionary
How to access data: Anonymized data collected for the study and additional documents (e.g. study protocol, informed consent form) may be made available to others upon request and after the approval of a proposal by the AMYPAD consortium.
When available: beginning date: 10-01-2022

Supporting Documents
Document types: None

Additional Information
Who can access the data: Data will be made available to researchers whose proposed use of the data has been approved.
Types of analyses: Data will be made available for any purpose.
Mechanisms of data availability: Data will be made available after approval of a proposal.
